# Unraveling the Pathobiological Role of the Fungal KEOPS Complex in Cryptococcus neoformans

**DOI:** 10.1128/mbio.02944-22

**Published:** 2022-11-15

**Authors:** Yeseul Choi, Eunji Jeong, Dong-Gi Lee, Jae-Hyung Jin, Yee-Seul So, Seong-Ryong Yu, Kyung-Jo Lee, Yoonjie Ha, Chi-Jan Lin, Ying-Lien Chen, Jun Bae Park, Hyun-Soo Cho, Anna F. Averette, Joseph Heitman, Kyu-Ho Lee, Kangseok Lee, Yong-Sun Bahn

**Affiliations:** a Department of Biotechnology, College of Life Science and Biotechnology, Yonsei University, Seoul, South Korea; b Department of Life Science, College of Natural Science, Sogang University, Seoul, South Korea; c Department of Life Science, Chung-Ang University, Seoul, South Korea; d Department of Plant Pathology and Microbiology, National Taiwan University, Taipei, Taiwan; e Department of Systems Biology, College of Life Science and Biotechnology, Yonsei University, Seoul, South Korea; f Department of Molecular Genetics and Microbiology, Duke University Medical Center, Durham, North Carolina, USA; g Department of Medicine, Duke University Medical Center, Durham, North Carolina, USA; h Department of Pharmacology and Cancer Biology, Duke University Medical Center, Durham, North Carolina, USA; Universidade de Sao Paulo

**Keywords:** Bud32, Cgi121, Kae1, Pcc1

## Abstract

The KEOPS (kinase, putative endopeptidase, and other proteins of small size) complex has critical functions in eukaryotes; however, its role in fungal pathogens remains elusive. Herein, we comprehensively analyzed the pathobiological functions of the fungal KEOPS complex in Cryptococcus neoformans (Cn), which causes fatal meningoencephalitis in humans. We identified four CnKEOPS components: Pcc1, Kae1, Bud32, and Cgi121. Deletion of *PCC1*, *KAE1*, or *BUD32* caused severe defects in vegetative growth, cell cycle control, sexual development, general stress responses, and virulence factor production, whereas deletion of *CGI121* led to similar but less severe defects. This suggests that Pcc1, Kae1, and Bud32 are the core KEOPS components, and Cgi121 may play auxiliary roles. Nevertheless, all KEOPS components were essential for C. neoformans pathogenicity. Although the CnKEOPS complex appeared to have a conserved linear arrangement of Pcc1-Kae1-Bud32-Cgi121, as supported by physical interaction between Pcc1-Kae1 and Kae1-Bud32, CnBud32 was found to have a unique extended loop region that was critical for the KEOPS functions. Interestingly, CnBud32 exhibited both kinase activity-dependent and -independent functions. Supporting its pleiotropic roles, the CnKEOPS complex not only played conserved roles in t^6^A modification of ANN codon-recognizing tRNAs but also acted as a major transcriptional regulator, thus controlling hundreds of genes involved in various cellular processes, particularly ergosterol biosynthesis. In conclusion, the KEOPS complex plays both evolutionarily conserved and divergent roles in controlling the pathobiological features of C. neoformans and could be an anticryptococcal drug target.

## INTRODUCTION

Kinase, putative endopeptidase, and other proteins of small size (KEOPS), also known as an endopeptidase-like kinase chromatin-associated (EKC) complex, is an evolutionarily conserved protein complex that has critical cellular functions in both prokaryotes and eukaryotes (1). The KEOPS complex was first functionally characterized in Saccharomyces cerevisiae (2, 3). The yeast KEOPS complex is involved in telomere regulation (2), and functions as the transcription and chromatin complex that governs cell cycle progression and pheromone responses (3). The KEOPS complex is also involved in tRNA modification by catalyzing the *N*^6^-threonylcarbamoyladenosine (t^6^A) modification at position 37 of tRNAs, which recognizes ANN-codons (N; any nucleotide), using threonylcarbamoyladenylate (TCA) synthesized by the universal Sua5 enzyme as the substrate with ATP, threonine, and bicarbonate. This t^6^A modification strengthens the A-U codon-anticodon base pair, and an impaired t^6^A modification leads to increased frameshift mutations and abnormal start codon recognition (4, 5). This evolutionarily conserved event is observed in some bacteria, archaea, and eukaryotes.

In the KEOPS complex, which was first named by Downey et al. in 2006 (2), Cgi121 (the homolog of human CGI-121) was identified using a synthetic genetic array as a suppressor of *cdc13-1*, an allele of the telomere-capping protein Cdc13. Like the human CGI-121, which physically interacts with p53-related protein kinase (PRPK) (6), the yeast Cgi121 also interacts with the yeast PRPK ortholog Bud32 (bud-site selection 32) (2). Subsequently, two additional subunits, Gon7 (a small protein of unknown function, also known as Pcc2) and Kae1 (kinase-associated endopeptidase 1), form a complex with Bud32 and Cgi121 (2). These yeast KEOPS components and Pcc1 (polarized growth chromatin-associated controller 1) were independently identified as subunits of the EKC complex that was named by Kisseleva-Romanova et al. (3). Interactions between Pcc1, Kae1, Bud32, Gon7, and Cgi121 were further supported by a large-scale analysis of yeast complexes (7, 8), and yeast two-hybrid analyses (9). Recent structural analysis revealed that the yeast KEOPS complex has a pentameric structure, with a linear arrangement of subunits in the order: Gon7-Pcc1-Kae1-Bud32-Cgi121 (10). In the pentameric KEOPS complex, Bud32, Pcc1, and Kae1 contribute to tRNA binding, and Kae1 transfers the threonylcarbamoyl moiety from TCA to tRNAs (10).

Pcc1, Kae1, and Bud32 are central KEOPS components universally conserved from archaea to humans. Notably, Kae1 and Bud32 often fuse to form a single bifunctional protein in several archaea (9), implying that they are structurally and functionally correlated. Kae1 has a putative Zn-binding endopeptidase activity. Bud32 and its homologs belong to the RIO-type family of atypical small kinases lacking the kinase activation loop (11). They are considered an ancestor of eukaryotic protein kinases and exist in eukaryotes and archaea but not bacteria (12). Unlike most Ser/Thr protein kinases that recognize phosphoacceptor sites specified by basic and/or prolyl residues, Bud32 homologs phosphorylate acidic proteins and peptides. Deletion of *BUD32* causes severe growth and sporulation defects (13, 14). Human PRPK cannot fully complement a yeast *BUD32* deletion mutant (15), indicating that Bud32 orthologs have functional divergence among eukaryotes. PRPK is markedly expressed in testicular tissues, weakly expressed in heart, kidney, and spleen tissues, and not normally expressed in other human tissues. However, PRPK is highly expressed in pancreatic, breast, and prostatic cancer cell lines. Unlike yeast Bud32, which only contains the protein kinase motif, human PRPK additionally contains the bipartite nuclear-localization signal motif (15). In the nucleus, PRPK binds to and phosphorylates p53. In *Drosophila*, PRPK serves as a signaling transducer of the PI3K/TOR pathway and controls bulk endocytosis and autophagy (16).

Despite its importance in eukaryotes, little is known about the function and regulatory mechanism of the KEOPS complex in fungal pathogens. In Cryptococcus neoformans, which causes fatal fungal meningoencephalitis and is responsible for more than 220,000 infections and 180,000 deaths globally each year (17), we first characterized the KEOPS subunit Bud32 as part of a global protein kinase functional survey (18). We focused on the atypical protein kinase due to its pleiotropic roles in C. neoformans. Bud32 is required but is not essential for the growth of C. neoformans and the deletion of *BUD32* causes severe defects in the production of virulence factors (capsule, melanin, and urease), sexual differentiation, sterol biosynthesis, and pathogenicity (18). A Bud32 ortholog in fungal pathogens was also characterized in the filamentous fungus, Aspergillus fumigatus, wherein the ortholog, which was named PipA (PtkA-interacting protein A), is a binding partner for the cyclin-dependent kinase PtkA, a Cdk9 homolog, which functions in conidiophore development (19). Unlike the nonessential S. cerevisiae Bud32, PipA is essential in A. fumigatus (19), but its function has not been elucidated yet. In Fusarium graminearum, a plant fungal pathogen that causes head blight disease, the Bud32 ortholog, FgBud32, is required for aerial hyphal growth and branching, development of the conidium and perithecia, and pathogenesis (20). Nevertheless, the functions of the KEOPS complex in fungal pathogens have not been investigated mechanistically.

In this study, we comprehensively analyzed the pathobiological functions of the KEOPS complex components, Pcc1, Kae1, Bud32, and Cgi121, and its complex downstream signaling network in C. neoformans. We demonstrate that the KEOPS complex is not only important for growth and general stress response and adaptation but also critical for sterol biosynthesis, tRNA modification, transcriptional regulation, and pathogenicity of C. neoformans.

## RESULTS

### Identification of the KEOPS components and their roles in the growth and development of C. neoformans.

In S. cerevisiae, five KEOPS complex components, including Gon7, Pcc1, Kae1, Bud32, and Cgi121, have been identified (10). Among these, we previously identified the Bud32 kinase (CNAG_02712) in C. neoformans (18). To further analyze the function of the CnKEOPS complex, we attempted to identify the remaining components of the KEOPS complex, including Kae1, Pcc1, Cgi121, and Gon7 orthologs. We performed a BLAST search (blastp) with the S. cerevisiae Kae1 (YKR038C), Cgi121 (YML036W), Pcc1 (YKR095W-A), and Gon7 (YJL184W) protein sequence in the C. neoformans H99 strain genome database of the fungiDB (http://fungidb.org/fungidb/) and identified C. neoformans Kae1 (CNAG_00364), Pcc1 (CNAG_04667), and Cgi121 (CNAG_01507) orthologs, but not Gon7 (see Fig. S1A–C in the supplemental material).

10.1128/mbio.02944-22.1 FIG S1 Phylogenetic and genotypic analyses of *PCC1*, *KAE1*, and *CGI121*. Phylogenetic analysis of KEOPS component orthologs in fungal species and human. (A–C) Phylogenetic trees for Kae1 (A), Pcc1 (B), and Cgi121 (C) orthologs. Each protein sequence was obtained from FungiDB (https://fungidb.org). (D–F) Genotypic analyses of *pcc1*Δ, *kae1*Δ, and *cgi121*Δ mutants. Gene disruption strategies are illustrated in the left panel. Each gene was disrupted using a deletion cassette with the *NAT*-resistance marker. Results from diagnostic PCR for checking 5′-end recombination, internal deletion, and 3′-end recombination of each target gene are shown in the middle panel. For Southern blot analysis (right panel), genomic DNA isolated from the wild-type (H99) and *pcc1*Δ mutants (YSB4930 and YSB4931) was digested with PvuI and ApaLI (D). Genomic DNA isolated from the wild-type (H99) and the *kae1*Δ mutants (YSB4863 and YSB4864) was digested with NdeI (E). Genomic DNA isolated from the wild-type (H99) and *cgi121*Δ strains (YSB5037 and YSB5038) was digested with BanI (F). Download FIG S1, PDF file, 0.3 MB. Copyright © 2022 Choi et al. 2022 Choi et al. https://creativecommons.org/licenses/by/4.0/ This content is distributed under the terms of the Creative Commons Attribution 4.0 International license.

**FIG 1:**
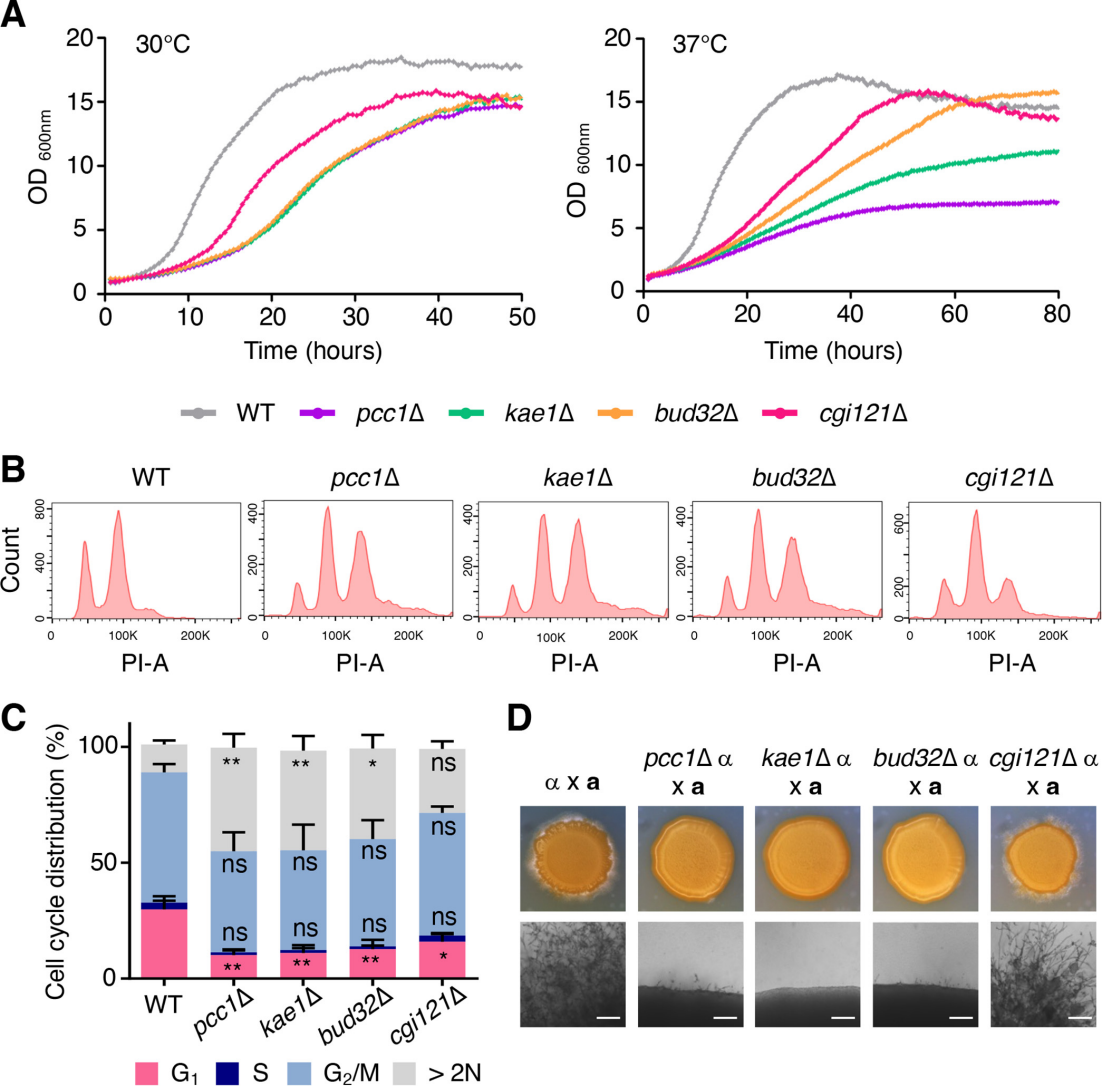
The roles of the KEOPS complex in the growth and sexual differentiation of C. neoformans. (A) The growth rates of wild type (WT) and mutants *pcc1*Δ (YSB4930), *kae1*Δ (YSB4863), *bud32*Δ (YSB1968), and *cgi121*Δ (YSB5037) were quantitatively measured at 30°C or 37°C using a multichannel bioreactor. (B, C) Cell cycle phases of propidium iodide (PI)-stained cells were quantitatively measured using flow cytometry. The flow cytometry graphs show representative data of three biological replicates. (B) The cell populations were represented by interphase (G_1_ peak and S phase) and mitotic phase (G_2_/M peak). The proportion of cell populations from the same experiment was quantified and is shown in the graph. (C) One-way ANOVA was used to determine the statistically significant differences between the wild type and mutants. Error bars indicate SEM. (ns, not significant; *, *P* = 0.01–0.05, **, *P* = 0.001–0.01). (D) Filamentation and mating efficiency of the KEOPS mutants. The following *MAT*α and *MAT***a** strains were cocultured on V8 medium (pH 5.0) for 10 days at room temperature in the dark: α (H99) × **a** (KN99**a**), α *pcc1*Δ × **a**, α *kae1*Δ × **a**, α *bud32*Δ × **a**, and α *cgi121*Δ × **a**. One representative data from two independent experiments (see Fig. S2D and E) is shown here.

To analyze the function of the KEOPS complex in C. neoformans, we constructed two independent deletion mutants for the *KAE1*, *PCC1*, and *CGI121* genes in the *MAT*α C. neoformans H99 strain background (see Fig. S1D–F) and analyzed their phenotypic traits in comparison with those of the *bud32*Δ mutants. The *bud32*Δ, *kae1*Δ, and *pcc1*Δ mutants exhibited similar severe growth defects even at 30°C compared with the wild-type strain, whereas the *cgi121*Δ mutant showed moderate growth defects (Fig. 1A and Fig. S2A–B). At the host physiological temperature (37°C), the *pcc1*Δ mutant showed the most evident growth defects, followed by *kae1*Δ, *bud32*Δ, and *cgi121*Δ mutants (Fig. 1A). Complementation with each mCherry-tagged wild-type allele successfully restored the normal growth in the KEOPS mutants (see Fig. S3). Supporting this, fluorescence-activated cell sorting (FACS) analysis of the wild-type and KEOPS mutant strains grown to the early logarithmic phase revealed that all the KEOPS complex mutants showed significantly decreased G_1_ phase (Fig. 1B and C; Fig. S2C). The *pcc1*Δ, *kae1*Δ, and *bud32*Δ mutants exhibited additional peaks indicating more genetic material than 2N (Fig. 1B and C), suggesting that the CnKEOPS complex is involved in cell cycle control.

10.1128/mbio.02944-22.2 FIG S2 The role of the KEOPS complex in the growth, cell cycle control, and sexual differentiation of C. neoformans. (A, B) Each strain (WT [H99S], *bud32*Δ [YSB1968], *kae1*Δ [YSB4863], *pcc1*Δ [YSB4930], and *cgi121*Δ [YSB5037]) was incubated and monitored using the bioreactor at 30°C (A) or 37°C (B) for 50 to 80 h. (C) The cell cycle of KEOPS mutants was monitored using flow cytometry. Each cell synchronized to OD_600_ 0.8 was stained with propidium iodide and then 30,000 of them were counted. From left to right, the percentages of cell cycle distribution in each graph represent the G_1_, S, G_2_/M, and the amount of > 2N DNA content. (D, E) Filamentation and mating efficiency of the KEOPS mutants. The following *MAT*α and *MAT***a** strains were cocultured on V8 medium (pH 5.0) for 10 days at room temperature in the dark: α (H99) × **a** (KN99**a**), α *pcc1*Δ × **a**, α *kae1*Δ × **a**, α *bud32*Δ × **a**, and α *cgi121*Δ × **a**. Two independent mutants of each KEOPS component (*pcc1*Δ (YSB4930 and YSB4931), *kae1*Δ (YSB4863 and YSB4864), *bud32*Δ (YSB1968 and YSB1969), and *cgi121*Δ (YSB5037 and YSB5038)) were tested. (D) shows the representative data. Download FIG S2, PDF file, 1.2 MB. Copyright © 2022 Choi et al. 2022 Choi et al. https://creativecommons.org/licenses/by/4.0/ This content is distributed under the terms of the Creative Commons Attribution 4.0 International license.

10.1128/mbio.02944-22.3 FIG S3 Phenotypic analysis of KEOPS mutants and their complemented strains. Each strain (mutants: *pcc1*Δ [YSB4930], *kae1*Δ [YSB4863], *bud32*Δ [YSB1968], and *cgi121*Δ [YSB5037] and complemented strains: *pcc1*Δ::*PCC1-mCherry* [YSB10003], *kae1*Δ::*KAE1-mCherry* [YSB7754], *bud32*Δ::*BUD32-mCherry* [YSB6353], and *cgi121*Δ::*CGI121-mCherry* [YSB7897]) was cultured in YPD broth at 30°C overnight, serially diluted 10-fold (dilution factor: 1 to 10^4^), and spotted onto YP or YPD solid media containing the following chemicals: 3 μg/mL fludioxonil (FDX), 600 μg/mL flucytosine (5FC), 0.8 μg/mL amphotericin B (AmB), 18 μg/mL fluconazole (FCZ), 2 mM diamide (DA), 0.03 mM menadione (MD), 3 mM hydrogen peroxide (H_2_O_2_), 120 mM hydroxyurea (HU), 16 mM dithiothreitol (DTT), 0.03% sodium dodecyl sulfate (SDS), 1% Congo red (CR), 5 mg/mL calcofluor white (CFW), 1 − 1.5 M KCl, 1 − 1.5 M NaCl, or 2 M sorbitol. Spotted plates were further incubated at 30°C for 4 days, and the representative images show the plates after 3 days. To determine the thermotolerance, the plates were incubated at 25°C, 30°C, 37°C, and 39°C. Download FIG S3, PDF file, 0.8 MB. Copyright © 2022 Choi et al. 2022 Choi et al. https://creativecommons.org/licenses/by/4.0/ This content is distributed under the terms of the Creative Commons Attribution 4.0 International license.

Cryptococcus neoformans has two mating types, *MAT*α and *MAT***a**, and undergoes sexual differentiation to produce infectious basidiospores. When mated with the *MAT***a** wild-type strain (KN99**a**), *MAT*α *bud32*Δ, *kae1*Δ, and *pcc1*Δ mutants did not generate any filamentous growth (Fig. 1D). In contrast, the *cgi121*Δ mutant produced intermediate levels of filamentation (Fig. 1D). Collectively, the CnKEOPS complex consists of Pcc1, Kae1, Bud32, and Cgi121 that play critical roles in the growth and development of C. neoformans.

### The role of the KEOPS complex in stress responses and virulence of C. neoformans.

**FIG 2:**
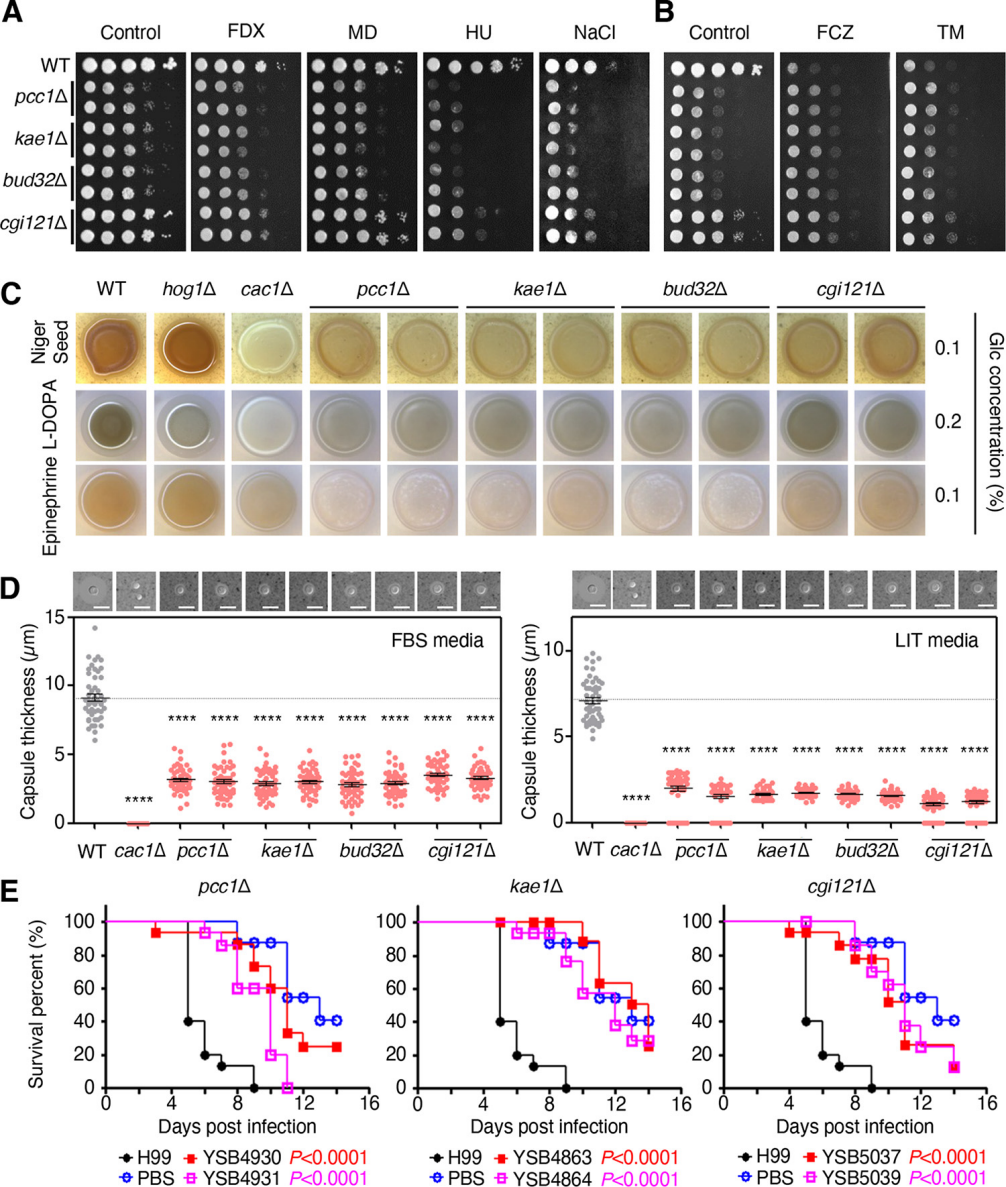
The roles of the KEOPS complex in stress responses, virulence factor production, and the pathogenicity of C. neoformans. (A, B) Stress sensitivity of the wild type (H99S) and KEOPS complex mutants (*pcc1*Δ [YSB4930 and YSB4931], *kae1*Δ [YSB4863 and YSB4864], *bud32*Δ [YSB1968 and YSB1969], and *cgi121*Δ [YSB5037 and YSB5038]). Each strain was cultured overnight in YPD broth at 30°C, serially diluted 10-fold, and then spotted onto YPD medium containing the following chemicals: 1 μg/mL fludioxonil (FDX), 16 μg/mL fluconazole (FCZ), 0.02 mM menadione (MD), 100 mM hydroxyurea (HU), 1 M NaCl, or 4 μg/mL tunicamycin (TM). The plates were incubated at 30°C for 4 to 6 days. (C) Melanin production assay. Indicated strains were spotted onto Niger seed, L-DOPA, and epinephrine agar medium containing 0.1% or 0.2% glucose, incubated at 37°C, and photographed after 1 to 2 days. *hog1*Δ and *cac1*Δ were used as control strains and showed enhanced and reduced melanin production, respectively. Representative images from three independent experiments are shown here. (D) Capsule production assay. Indicated strains were cultured in capsule-inducing media (Fetal bovine serum [FBS] and Littman’s [LIT] medium) at 37°C for 2 days. *cac1*Δ is an acapsular mutant. The cells were scraped and observed using India ink staining. Capsule thickness (total diameter − cell body diameter) was measured for 50 WT and mutant cells (*n *= 50). Statistical significance was calculated using one-way ANOVA analysis with Bonferroni’s multiple-comparison test. Data are presented as mean values ± SEM (****, *P* < 0.0001). One representative graph is shown. (E) Insect-killing assay for measuring the virulence of KEOPS mutants. *P* values were calculated using the log-rank (Mantel–Cox) test to measure statistical differences between WT strain (H99S) and each mutant strain.

Next, we addressed the role of the KEOPS complex in stress response and adaptation and production of two major virulence factors, melanin pigment and polysaccharide capsule, which contribute to the pathogenicity of C. neoformans. The *bud32*Δ, *kae1*Δ, and *pcc1*Δ mutants exhibited increased susceptibility to a phenylpyrrole class of fungicide fludioxonil, a reducing agent dithiothreitol, oxidative stress agents (diamide and *tert-*butyl hydroperoxide), genotoxic agents (methyl methanesulfonate and hydroxyurea), and osmotic stresses (NaCl, KCl, sorbitol) but displayed increased resistance to fluconazole and an ER stress agent tunicamycin (Fig. 2A–B; Fig. S3 and S4A). All three mutants also exhibited highly reduced production of melanin (Fig. 2C; see Fig. S4B) and capsule (Fig. 2D; Fig. S4C). In contrast, the *cgi121*Δ mutant exhibited generally similar but milder phenotypic alterations than the other KEOPS mutants (Fig. 2A–D). Collectively, these results indicate that Pcc1, Bud32, and Kae1 play major roles in stress and antifungal drug responses and virulence factor production in C. neoformans, whereas Cgi121 has less pronounced but still evident roles.

10.1128/mbio.02944-22.4 FIG S4 The roles of the KEOPS complex in stress responses and virulence factor production in C. neoformans. (A) Stress sensitivity of wild-type (H99S) and KEOPS complex mutants *pcc1*Δ (YSB4930 and YSB4931), *kae1*Δ (YSB4863 and YSB4864), *bud32*Δ (YSB1968 and YSB1969), and *cgi121*Δ (YSB5037 and YSB5038). Each strain was cultured overnight in YPD broth at 30°C, serially diluted 10-fold, and then spotted onto YPD medium containing the indicated stress inducing agent. The plates were incubated at 30°C for 4 days. (B) Melanin production assay. Indicated strains were spotted onto Niger seed, L-DOPA, or epinephrine agar media containing 0.1% or 0.2% glucose, incubated at 37°C, and photographed after 1 to 2 days. Two representative images from three independent experiments are shown here. (C) Capsule production assay. Indicated strains were cultured in capsule-inducing media (FBS and LIT media) at 37°C for 2 days. As an acapsular strain control, the *cac1*Δ mutant was used. The cells were scraped and observed using India ink staining. Capsule thickness (total diameter − cell body diameter) was measured for 50 cells of WT and mutants (*n *= 50). Statistical significance was calculated using one-way ANOVA analysis with Bonferroni’s multiple-comparison test. Data are presented as mean values ± SEM (****, *P* < 0.0001). Two representative graphs from three independent experiments are shown here. Download FIG S4, PDF file, 0.5 MB. Copyright © 2022 Choi et al. 2022 Choi et al. https://creativecommons.org/licenses/by/4.0/ This content is distributed under the terms of the Creative Commons Attribution 4.0 International license.

Next, we examined the role of KEOPS components in the virulence of C. neoformans using the insect-killing assay. The *kae1*Δ and *pcc1*Δ mutants exhibited highly reduced virulence like the *bud32*Δ mutant (18) (Fig. 2E). Notably, the *cgi121*Δ mutants, which exhibited intermediate *in vitro* virulence-related phenotypes compared with other KEOPS mutants, also showed highly reduced virulence (Fig. 2E). Collectively, these results indicate that all KEOPS components are critical for the pathogenicity of C. neoformans.

### Cellular localization and physical interaction of KEOPS components in C. neoformans.

**FIG 3:**
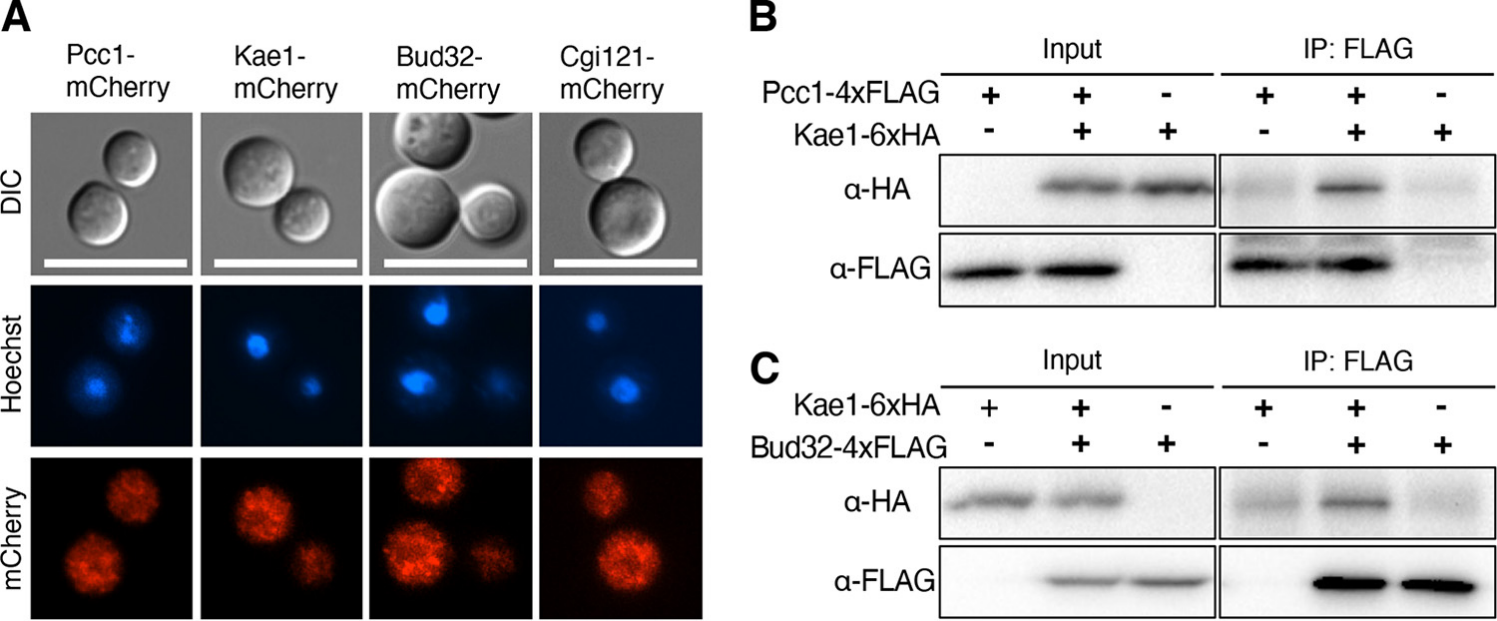
Cellular localization and *in vivo* interaction of KEOPS components. (A) Cellular localization of KEOPS components. The mCherry-tagged strains (*pcc1*Δ*::PCC1-mCherry* [YSB10003], *kae1*Δ*::KAE1-mCherry* [YSB7754], *bud32*Δ*::BUD32-mCherry* [YSB6353], and *cgi121*Δ*::CGI121-mCherry* [YSB7897]) were fixed and stained with Hoechst dye to visualize the nucleus. Scale bar = 10 μm. (B, C) *In vivo* interaction between KEOPS components. The 4xFLAG-tagged proteins in the whole lysate extracts from each strain (*bud32*Δ*::BUD32-4xFLAG* [YSB5302], *KAE1-6xHA-HYG* [YSB4716], *bud32*Δ*::BUD32-4xFLAG KAE1-6xHA-HYG* [YSB5176], *PCC1-4xFLAG-NAT* [YSB7829], and *KAE1-6xHA-HYG PCC1-4xFLAG-NAT* [YSB7832]) were immunoprecipitated with anti-FLAG antibody (IP; α-FLAG). Subsequently the 6xHA-tagged proteins were detected using western blot with anti-HA antibody (IB; α-HA).

Despite the role of S. cerevisiae KEOPS components in transcription and chromatin regulation during the cell cycle (3), Gon7, Pcc1, Kae1, Bud32, and Cgi121 localize in both the cytoplasm and nucleus during cell cycle progression (21). Therefore, we next addressed the cellular localization of the fungal KEOPS complex components in C. neoformans. We constructed the *pcc1*Δ*::PCC1-mCherry*, *kae1*Δ*::KAE1-mCherry*, *bud32*Δ*::BUD32-mCherry*, and *cgi121*Δ*::CGI121-mCherry* strains, where the mCherry protein was tagged at the C-terminal region of each allele (Fig. S5A–D). Reintegration of *PCC1-mCherry*, *KAE1-mCherry*, *BUD32-mCherry*, and *CGI121-mCherry* alleles successfully restored normal phenotypes in the *pcc1*Δ, *kae1*Δ, *bud32*Δ, and *cgi121*Δ mutants, respectively, indicating that each mCherry-tagged allele was functional (Fig. S3). Like in S. cerevisiae, we found that all KEOPS complex components localized in the cytoplasm and nucleus of C. neoformans (Fig. 3A).

10.1128/mbio.02944-22.5 FIG S5 Construction of KEOPS complemented and chromosomal tagged strains used in this study. To construct the mCherry tagged strain, each DNA sequence containing the promoter and ORF of *PCC1* (A), *KAE1* (B), *BUD32* (C), and *CGI121* (D) was subcloned into the pNEO_mCherry vector. The constructed plasmids were linearized with restriction enzymes described in Materials and Methods and then biolistically introduced into each deletion mutant (*pcc1*Δ [YSB4930], *kae1*Δ [YSB4863], *bud32*Δ [YSB1968], and *cgi121*Δ [YSB5037]). The targeted integration of each plasmid was confirmed with diagnostic PCR with indicated primer pairs (Dataset S1). To examine the *in vivo* interaction between KEOPS subunits, 4xFLAG or 6xHA were integrated into the C terminus of each KEOPS component. (E) The C terminus of Kae1 was tagged with 6xHA in both the wild-type and *bud32*Δ::*BUD32-4xFLAG* (YSB5176) strains and was confirmed using Southern blot analysis. (F) The C terminus of Pcc1 was tagged with 6xHA in both wild-type and *KAE1-6xHA-HYG* (YSB4716) strain and was confirmed using Southern blot analysis. (G) Wild-type (H99S), *pcc1*Δ (YSB4930), *kae1*Δ (YSB4863), *bud32*Δ (YSB1968), *PCC1-4xFLAG-NAT* (YSB7829), *KAE1-6xHA-HYG PCC1-4xFLAG-NAT* (YSB7832), *KAE1-6xHA-HYG* (YSB4716), *bud32*Δ::*BUD32-4xFLAG KAE1-6xHA-HYG* (YSB5176), and *bud32*Δ::*BUD32-4xFLAG* (YSB5302) strains were cultured overnight at 30°C in YPD broth, serially diluted 10-fold, and spotted onto YPD medium containing the following stress inducers: 1 μg/mL fludioxonil (FDX), 0.4 μg/mL amphotericin B (AmB), 18 μg/mL fluconazole (FCZ), 2 mM diamide (DA), 0.03 mM menadione (MD), 0.7 mM *tert*-butyl hydroperoxide (TBH), 120 mM hydroxyurea (HU), 0.03% sodium dodecyl sulfate (SDS), 5 mg/mL calcofluor white (CFW), 1 − 1.5 M KCl, 1 − 1.5 M NaCl, and 2 M sorbitol. (H) The *bud32*Δ::*BUD32-4xFLAG* strain was constructed by biolistically introducing the linearized pNEO_BUD32-4xFLAG into the WT strain. The targeted insertion of the plasmid was confirmed with diagnostic PCR. (I) The N terminus of Cgi121 was tagged with 6xHA by introducing the DNA product containing the 5′ UTR of *CGI121*, *HYR^r^* marker, promoter of *CGI121*, and 6xHA sequence. The correct integration was confirmed using Southern blot analysis. The 6xHA tag was integrated into the C terminus of Cgi121 was confirmed using Southern blot analysis. (J) Each strain, including WT (H99), *bud32*Δ (YSB1968), *cgi121*Δ (YSB5037), *bud32*Δ::*BUD32-4xFLAG* (YSB5302), *CGI121-6xHA-HYG* (YSB7726), *bud32*Δ::*BUD32-4xFLAG CGI121-6xHA-HYG* (YSB8177), *HYG*-*6xHA*-*CGI121* (YSB9483), and *bud32*Δ::*BUD32-4xFLAG HYG-6xHA*:*CGI121* (YSB9482), was cultured overnight at 30°C in YPD broth, diluted 10-fold, and spotted onto the YPD media containing the following stress inducers: 1 μg/mL FDX, 0.4 μg/mL AmB, 20 μg/mL FCZ, 3 mM DA, 3 mM H_2_O_2_, 16 mM DTT, 100 mM of HU, 0.02% of SDS, 1% Congo red (CR), and 1 − 1.5 M KCl, 1 − 1.5 M NaCl, or 2 M sorbitol. The plates were incubated at 30°C for 4 days. (K, L) Coimmunoprecipitation analysis was used to confirm the *in vivo* interaction between Pcc1 and Kae1 (K) or Kae1 and Bud32 (L). Two independent data are shown here. Download FIG S5, PDF file, 1.5 MB. Copyright © 2022 Choi et al. 2022 Choi et al. https://creativecommons.org/licenses/by/4.0/ This content is distributed under the terms of the Creative Commons Attribution 4.0 International license.

The fact that Bud32, Kae1, Pcc1, and Cgi121 play redundant functions suggests that the four components may form a complex in C. neoformans. In S. cerevisiae, Gon7-Pcc1-Kae1-Bud32-Cgi121 has a linear arrangement (10, 22). To verify the linear arrangement of the KEOPS components in C. neoformans, first we examined whether Pcc1 and Kae1 interact with each other using a coimmunoprecipitation (co-IP) experiment. We constructed the *KAE1:KAE1-6xHA*, where the 6xHA was tagged at the C terminus of Kae1 (see Fig. S5E). Subsequently, we tagged the C-terminal region of the *PCC1* allele with 4xFLAG in both wild-type and the *KAE1:KAE1-6xHA* strains (see Fig. S5F). Chromosomal tagging of Pcc1 and Kae1 with 4xFLAG and 6xHA, respectively, did not cause growth defects in C. neoformans (Fig. S5G), indicating that these alleles were functional. Co-IP analysis revealed that Pcc1 physically interacted with Kae1 in C. neoformans (Fig. 3B).

We next examined whether Bud32 interacts with Kae1 in C. neoformans. We constructed the *bud32*Δ*::BUD32:4xFLAG* strain, where the 4xFLAG was tagged at the C terminus of Bud32 (Fig. S5H). Reintegration of the *BUD32:4xFLAG* allele successfully restored normal phenotypes in the *bud32*Δ mutant (Fig. S5G), indicating that the Bud32-4xFLAG allele was functional. Subsequently, we tagged the C-terminal region of the *KAE1* allele with 6xHA in the *bud32*Δ*::BUD32:4xFLAG* strain (Fig. S5E). Co-IP analysis revealed that Kae1 physically interacted with Bud32 in C. neoformans (Fig. 3C).

Lastly, we addressed whether Bud32 interacts with Cgi121 by tagging the N- or C-terminal region of the *CGI121* allele with 6xHA in the wild-type and *bud32*Δ::*BUD32:4xFLAG* strains (Fig. S5I). The N-terminal chromosomal tagging of Cgi121 with 6xHA (6xHA-Cgi121) did not restore normal phenotypes in the *cgi121*Δ mutant (Fig. S5J), indicating that 6xHA-Cgi121 was not functional. In contrast, the C-terminal chromosomal tagging of Cgi121 with 6xHA (Cgi121-6xHA) restored normal phenotypes in the *cgi121*Δ mutant (Fig. S5J), indicating that Cgi121-6xHA was functional. However, cells having both Bud32-4xFLAG and Cgi121-6xHA alleles exhibited minor growth defects and increased stress susceptibility (Fig. S5J), implying that these two tagged Bud32 and Cgi121 proteins may have a problem in normal interaction. We did not find any interaction between Bud32 and Cgi121 through co-IP analysis (data not shown). Altogether, the KEOPS complex in C. neoformans appears to be composed of a linear arrangement of Pcc1-Kae1-Bud32, and Cgi121 may transiently bind, if at all, to Bud32.

### The essential role of the extended loop region of Bud32 for KEOPS complex functionality in C. neoformans.

**FIG 4:**
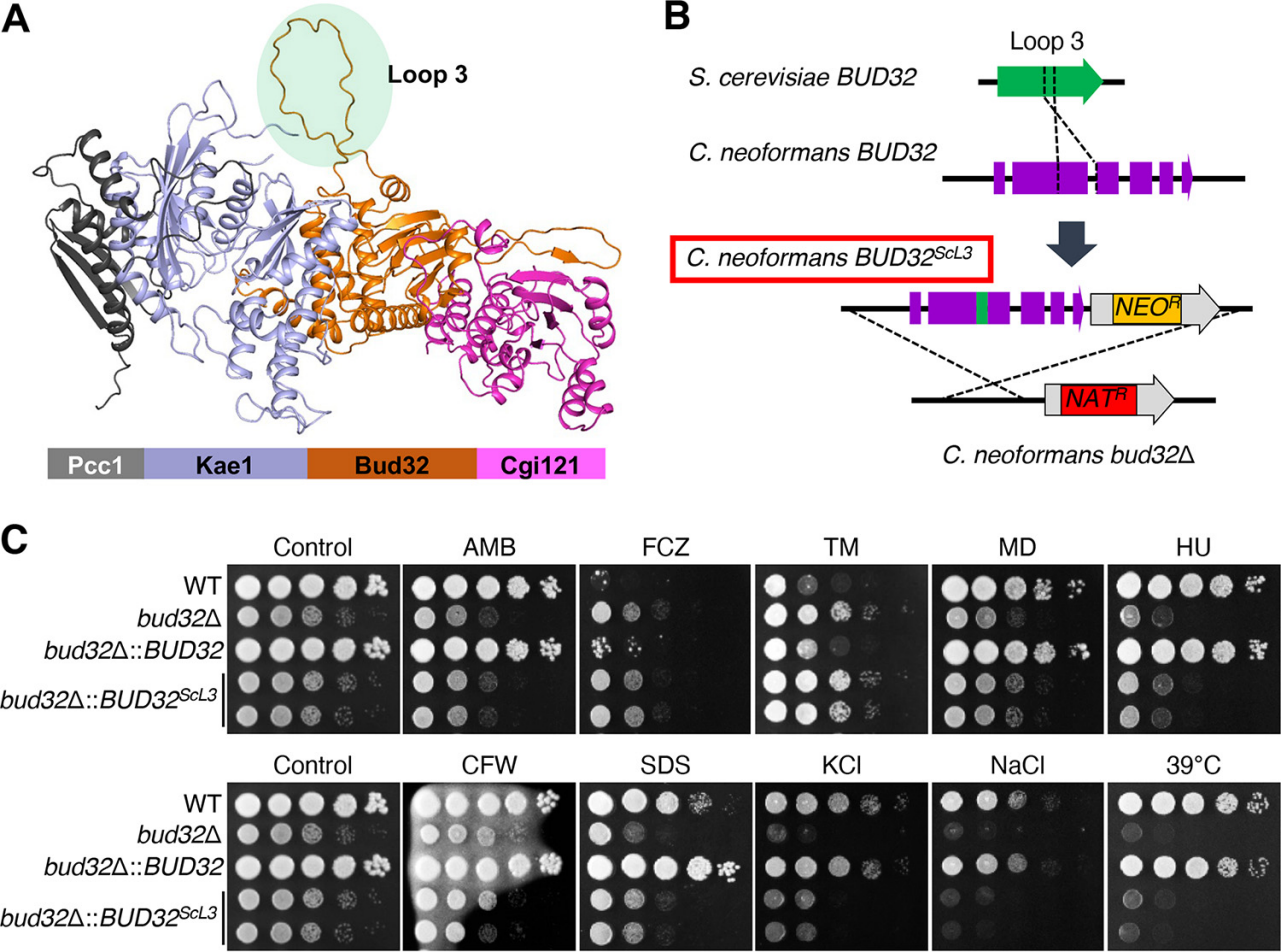
The role of the unique extended loop 3 region of cryptococcal Bud32. (A) Predicted cryptococcal KEOPS complex structure. The tetra-heterocomplex structure of C. neoformans Pcc1, Kae1, Bud32, and Cgi121 was predicted using AlphaFold2 multimer. Query sequence was ordered, and the MSA option and Advanced settings were used as default. The extended loop 3 region, characteristic of cryptococcal Bud32, is marked with a green circle. (B) The unique extended loop 3 region of cryptococcal Bud32 was replaced with the short loop 3 region of S. cerevisiae. The yeast Bud32 sequence of L113 to T140 was introduced between S114 and H172 of cryptococcal Bud32. (C) Each strain (WT, *bud32*Δ [YSB1968], *bud32*Δ::*BUD32* [YSB4662], and *bud32*Δ::*BUD32^Loop3^* [YSB6211 and YSB6209]) was cultured overnight in liquid YPD medium at 30°C, serially diluted 10-fold, spotted onto YPD medium containing 0.8 μg/mL amphotericin (AMB), 22 μg/mL FCZ, 0.4 μg/mL TM, 0.03 mM MD, 120 mM HU, 5 mg/mL CFW, 0.02% sodium dodecyl sulfate (SDS), 1.5 M KCl, or 1.5 M NaCl, and incubated at 30°C for 3 days. For the high-temperature sensitivity assay, 10-fold serially diluted cells were spotted on YPD medium and then incubated at 39°C for 3 days.

Based on physical interaction data between Pcc1, Kae1, and Bud32, we attempted to predict the CnKEOPS complex structure using AlphaFold2 multimer (23). The CnKEOPS complex appears to have a linear arrangement in the order Pcc1-Kae1-Bud32-Cgi1 that is highly similar to that of the yeast KEOPS complex (22) (Fig. 4A). However, comparing cryptococcal and yeast KEOPS complex structures reveals that CnBud32 contains an internal extended loop structure (Loop 3) not observed in the yeast Bud32 (Fig. 4A). The loop 3 is composed of 59 amino acids (V114–M172), including 16 glutamate residues conferring it a high negative charge. To examine whether the extended loop 3 in CnBud32 is important for Bud32 function, we replaced it with the corresponding short loop 3 region of yeast Bud32 (*BUD32^ScL3^*) and constructed the *bud32*Δ::*BUD32^ScL3^* strain using complementation (Fig. 4B; Fig. S6A–B). Notably, reintegration of the *BUD32^ScL3^* allele failed to restore the phenotypes of the *bud32*Δ mutant (Fig. 4C), indicating that the extended loop 3 region is critical for the function of CnBud32. Collectively, cryptococcal Bud32 has a structurally unique feature compared with yeast Bud32.

10.1128/mbio.02944-22.6 FIG S6 The unique extended loop 3 region of Cryptococcal Bud32 and its functions. (A) Sequence alignment of fungal Bud32 orthologs. The fungal Bud32 sequences were obtained from Candida albicans (C5_02440C_A), Candida glabrata (CAGL0F08415g), Saccharomyces cerevisiae (YGR262C), Neurospora crassa (NCU04595), Cryptococcus neoformans (CNAG_02712), Cryptococcus deneoformans (CNK01970), and Cryptococcus *deuteogattii* (CNBG_3082). The alignment was created using Bioedit program. The extended loop 3 region of Bud32, observed only in Cryptococcus species, is shown here. (B) The extended loop 3 of C. neoformans Bud32 was replaced by the short loop of S. cerevisiae Bud32. The pNEO_Bud32^ScL3^ was linearized and integrated into *bud32*Δ (YSB1968). The targeted integration was confirmed with diagnostic PCR. (C) Purified recombinant Bud32 proteins were confirmed through SDS-polyacrylamide electrophoresis. Purified proteins were subjected to the gel with control samples and TEV-untreated proteins. TEV-treated proteins were detected at 31.7 kDa, molecular weight of Bud32. TEV untreated proteins were detected above 40 kDa, because rBud32 is still tagged with Trx. In the right-most lane, ‘2^nd^ NB’ lane, purified proteins were rBud32, seen at the 31.7 kDa. (D) To construct the Bud32 kinase-dead mutant, pNEO_Bud32^K54A^ was linearized and integrated into *bud32*Δ (YSB1968). The targeted integration of gene was confirmed using diagnostic PCR. (E, F) The growth rate of wild-type (H99), *bud32*Δ (YSB1968), *bud32*Δ::*BUD32* (YSB4662), and *bud32*Δ::*BUD32^K54A^* (YSB5874) was measured using bioreactor at 30°C (E) and 37°C (F). After the OD_600_ of strains was adjusted to 0.8, the strains were incubated in bioreactor for 50 − 80 h. Results for the two of three biological replicates are shown here. (G, H) The role of the Bud32 kinase activity in melanin and capsule production. The melanin pigments in each strain (WT (H99S), *bud32*Δ (YSB1968), *bud32*Δ::*BUD32* (YSB4662), and *bud32*Δ::*BUD32^K54A^* (YSB5874)) were induced at 37°C using Niger seed, epinephrine, or L-DOPA media containing 0.1% or 0.2% glucose, and photographed after 2 to 3 days (G). For capsule production assay, each strain was spotted on FBS agar medium and incubated at 37°C for 2 days (H). Statistical significance was calculated using one-way ANOVA analysis with Bonferroni’s multiple-comparison test. Data are presented as mean values ± SEM (****, *P* < 0.0001; NS, not significant). Results for the two of three biological replicates are shown here. (J) Mating assay. The following *MAT*α and *MAT***a** strains were cocultured on V8 medium (pH 5.0) for 10 to 12 days at room temperature in the dark: α (H99) × **a** (KN99**a**), α *bud32*Δ × **a**, α *bud32*Δ::*BUD32 *× **a**, and α *bud32*Δ::*BUD32^K54A^* × **a**. Download FIG S6, PDF file, 1.0 MB. Copyright © 2022 Choi et al. 2022 Choi et al. https://creativecommons.org/licenses/by/4.0/ This content is distributed under the terms of the Creative Commons Attribution 4.0 International license.

### Kinase activity-dependent and -independent roles of Bud32 in C. neoformans.

**FIG 5:**
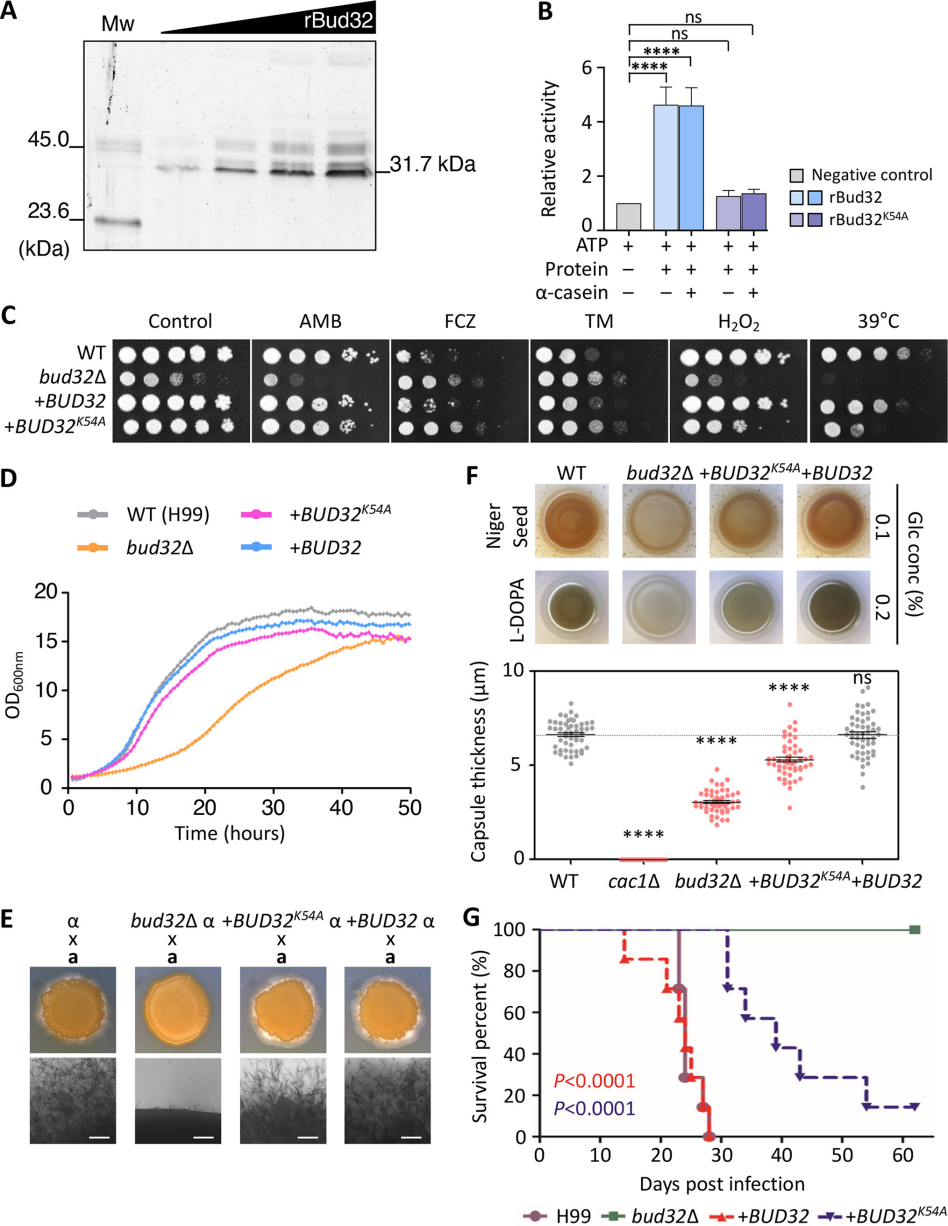
The kinase activity-dependent and -independent roles of cryptococcal Bud32. (A) Autophosphorylation of Bud32 shown on SDS-polyacrylamide gel where the increasing amount of recombinant Bud32 (rBud32) protein (0.2, 0.5, 1.0, or 2.0 μg) incubated in the presence of 50 μM ATP was loaded. *M*_w_, PeppermintStick phosphoprotein molecular weight standards. (B) Determination of Bud32 ATPase/kinase activity. The kinase activities of rBud32 and recombinant kinase-dead Bud32 (rBud32^K54A^) proteins are represented in blue and purple, respectively. ATPase (without substrate) or kinase activity (with substrate, 2 μg α-casein) was measured using 4 μg rBud32 or rBud32^K54A^ and 100 μM ATP. (C) The stress sensitivity of the Bud32 kinase-dead mutant. Each strain (WT [H99S], *bud32*Δ [YSB1968], *bud32*Δ::*BUD32* [YSB4662], and *bud32*Δ::*BUD32^K54A^* [YSB5874]) was cultured, diluted, and spotted as described in Fig. 2A onto YPD medium containing 1.4 μg/mL AMB, 22 μg/mL fluconazole, 0.5 μg/mL TM, or 0.02 mM MD, and then incubated at 30°C for 3 days. For the high-temperature sensitivity assay, 10-fold serially diluted cells were spotted on YPD medium and then incubated at 39°C for 3 days. (D) The growth rate of each indicated strain was quantitatively measured at 30°C for 50 h using a multichannel bioreactor. One representative graph from two independent experiments is shown here. (E) Mating assay. The following *MAT*α and *MAT***a** strains were cocultured on V8 medium (pH 5.0) for 10 days at room temperature in the dark: α (H99) × **a** (KN99**a**), α *bud32*Δ × **a**, α *bud32*Δ::*BUD32 *× **a**, and α *bud32*Δ::*BUD32^K54A^* × **a**. (F) The role of the Bud32 kinase activity in melanin and capsule production. The melanin pigments in each strain were induced at 37°C using Niger seed or L-DOPA medium containing 0.1% or 0.2% glucose, respectively, and photographed after 2 to 3 days. For capsule production assay, each strain was spotted on FBS agar medium and incubated at 37°C for 2 days. Statistical significance was calculated using one-way ANOVA analysis with Bonferroni’s multiple-comparison test. Data are presented as mean values ± SEM (****, *P* < 0.0001; NS, not significant). (G) The role of the Bud32 kinase activity in C. neoformans virulence. The survival rates of mice intranasally infected with the indicated strains were monitored for 63 days. Seven mice per strain were used.

Next, we checked whether the kinase activity of Bud32 is required for the function of the KEOPS complex in C. neoformans. We first examined whether Bud32 has kinase activity at all in C. neoformans. For this purpose, we expressed recombinant Bud32 protein in Escherichia coli (see Fig. S6C). After expressing and purifying Bud32 protein, we measured its kinase activity. Interestingly, we found that Bud32 undergoes autophosphorylation (Fig. 5A and B). The presence of a kinase substrate (α-casein) did not increase kinase activity in Bud32 (Fig. 5B).

We then questioned whether the kinase activity is essential for the pleiotropic function of Bud32. We constructed the kinase-dead allele of *BUD32* (*BUD32^K54A^*) through the site-directed mutation of K54A that is required for ATP binding (equivalent to S. cerevisiae K52 residue) and reintegrated it into the native *BUD32* locus (Fig. S6D). Surprisingly, reintegration of the *BUD32^K54A^* allele completely restored the growth of C. neoformans at 30°C, amphotericin B resistance, and mating efficiency like the wild-type *BUD32* allele (Fig. 5C–E). In contrast, the *bud32*Δ::*BUD32^K54A^* complemented strain had partially restored resistance to hydrogen peroxide and high temperature (39°C) and melanin and capsule production (Fig. 5F). However, the reintegration of the *BUD32^K54A^* allele failed to recover the susceptibility to fluconazole and tunicamycin (Fig. 5C).

As the kinase activity differentially contributed to the functions of Bud32, we checked whether Bud32 kinase activity is required for the virulence of C. neoformans using a murine model of systemic cryptococcosis (Fig. 5G). As expected from the previous signature-tagged mutagenesis (STM)-based murine infectivity and insect-killing assays (18), the *bud32*Δ mutant was avirulent upon intranasal infection of mice (Fig. 5G). In contrast, the *bud32*Δ::*BUD32* complemented strain was as virulent as the wild-type H99 strain, but the *bud32*Δ::*BUD32^K54A^* complemented strain showed an intermediate level of virulence (Fig. 5G), indicating that the Bud32 kinase activity partially contributes to the virulence of C. neoformans. Collectively, all these results suggest that Bud32 plays pleiotropic roles in C. neoformans in both kinase activity-dependent and -independent manners.

### The role of the KEOPS complex in the tRNA modification of C. neoformans.

**FIG 6:**
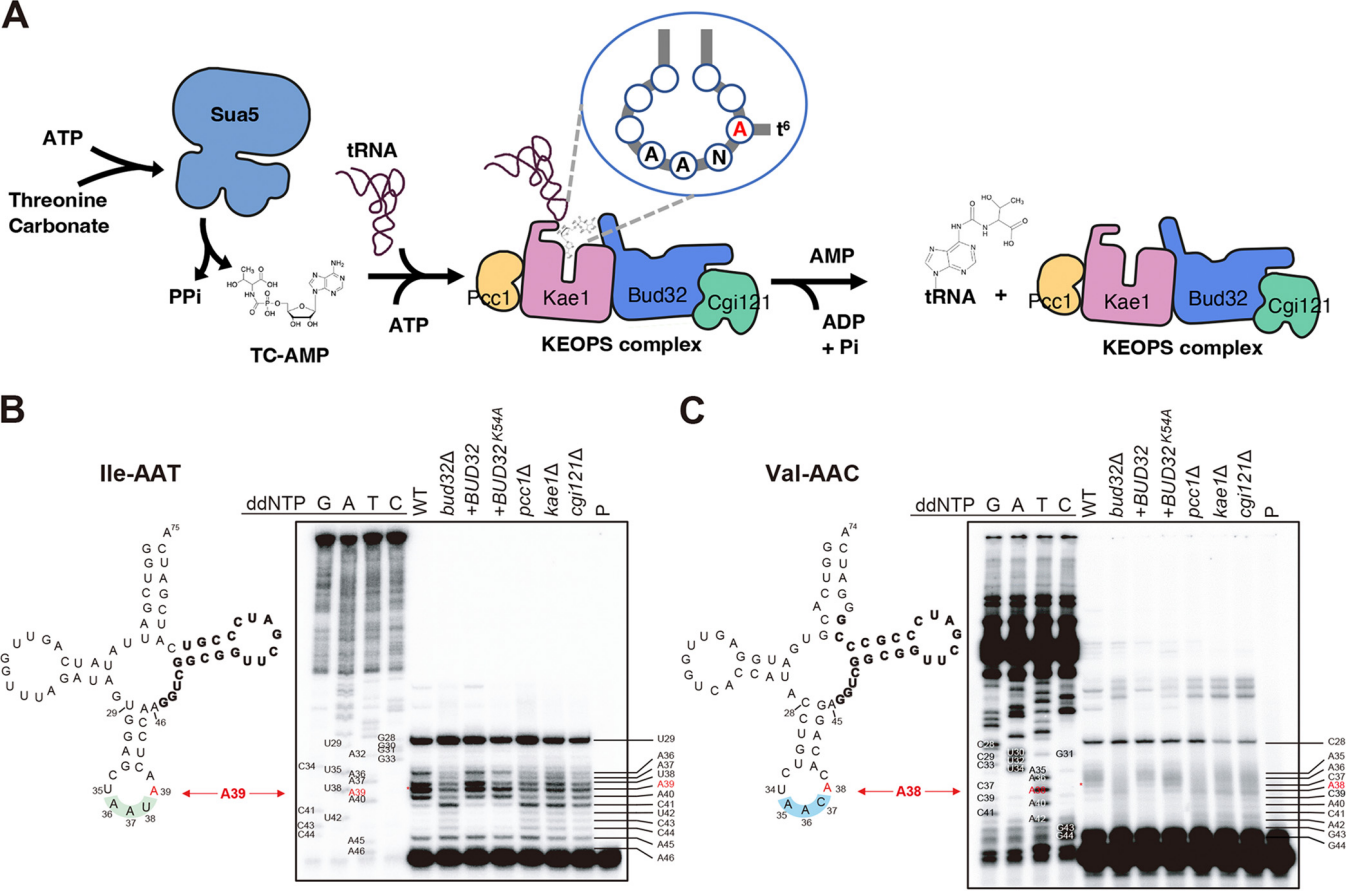
The role of the cryptococcal KEOPS complex in tRNA modification. (A) Schematic image of t^6^A modification by the KEOPS complex and Sua5. Sua5 catalyzes the production of the L-threonyl-carbamoyl-AMP (TC-AMP) intermediate using bicarbonate, threonine, and ATP. The KEOPS complex recognizes and binds the TC-AMP and tRNA. TC is transferred to the A position of tRNA with release of AMP by Kae1. Then, the KEOPS complex undergoes conformational change, driven by Bud32-mediated ATP hydrolysis, and releases the t^6^A-modified tRNA. (B) Primer extension analysis of tRNA Ile (AAU). Total RNAs isolated from WT, *bud32*Δ (YSB1968), *bud32*Δ::*BUD32* (YSB4662), *bud32*Δ::*BUD32^K54A^* (YSB5874), *pcc1*Δ (YSB4930), *kae1*Δ (YSB4863), and *cgi121*Δ (YSB5037) mutant strains were hybridized with a 5′-end labeled primer (Cn_Ile-AAT-R) and extended. Synthetic oligonucleotide (Cn_Ile-AAT-1-1) was used as the template for PCR. Cloverleaf structures of the Ile tRNA is shown. The position of the t^6^A-modified base (A39) in the Ile tRNA, marked by horizontal arrows, was confirmed through sequence ladders. The asterisk (*) indicates an A39 band. (C) Primer extension analysis of tRNA Val (AAC). Total RNAs isolated from strains described in (B) were hybridized with a 5′-end labeled primer (Cn_Val-AAC-R) and extended. Synthetic oligonucleotide (Cn_Val-AAC-1-1) was used as the template for PCR. Cloverleaf structures of the Val tRNA is shown. The asterisk (*) indicates a A38 band. (B, C) The specific primers used are complementary to the sequence shown in boldface in the cloverleaf structures. Sequencing ladders were produced using the same primer used in cDNA synthesis. The lane P was loaded with primer only.

Next, we addressed whether the CnKEOPS complex plays an evolutionarily conserved role in tRNA modification by catalyzing the t^6^A modification of tRNAs (summarized in Fig. 6A). For this purpose, we performed a primer extension analysis of a t^6^A-containing tRNA (Ile AAU) isolated from wild type, and *bud32*Δ, *bud32*Δ::*BUD32*, *bud32*Δ::*BUD32^K54A^*, *pcc1*Δ, *kae1*Δ, and *cgi121*Δ mutants. The primer extension profile of the *bud32*Δ mutant was markedly different at position 39, equivalent to position 37 of yeast tRNA (Ile AAU), from that of the wild type and *bud32*Δ::*BUD32* complemented strain (Fig. 6B; Fig. S7A). The primer extension profiles of the *pcc1*Δ, *kae1*Δ, and *cgi121*Δ mutants were similar to those of the *bud32*Δ mutant (Fig. 6B; Fig. S7A). The *bud32*Δ::*BUD32^K54A^* strain exhibited an intermediate primer extension profile between the wild type and *bud32*Δ mutant (Fig. 6B; Fig. S7A), suggesting that the kinase activity of Bud32 is required, but not essential, for the t^6^A modification. When we performed the primer extension analysis of a non-ANN codon-recognizing tRNA (Val AAC) isolated from the same set of strains, all KEOPS mutant strains exhibited very similar primer extension profiles (Fig. 6C and S7B). All these results indicate that the CnKEOPS components play a conserved role in t^6^A modification of ANN codon-recognizing tRNAs.

10.1128/mbio.02944-22.7 FIG S7 The role of the cryptococcal KEOPS complex in tRNA modification. (A) Primer extension analysis of tRNA Ile (AAU). Total RNAs isolated from WT, *bud32*Δ (YSB1968), *bud32*Δ::*BUD32* (YSB4662), *bud32*Δ::*BUD32^K54A^* (YSB5874), *pcc1*Δ (YSB4930), *kae1*Δ (YSB4863), and *cgi121*Δ (YSB5037) mutant strains were hybridized with a 5′-end labeled primer (Cn_Ile-AAT-R) and extended. Synthetic oligonucleotide (Cn_Ile-AAT-1-1) was used as the template for PCR. Cloverleaf structures of the Ile tRNA is shown. The position of the t^6^A-modified base (A39) in the Ile tRNA, marked by horizontal arrows, was confirmed using sequence ladders. The asterisk (*) indicates a A39 band. (B) Primer extension analysis of tRNA Val (AAC). Total RNAs isolated from strains described in (A) were hybridized with a 5′-end labeled primer (Cn_Val-AAC-R) and extended. Synthetic oligonucleotide (Cn_Val-AAC-1-1) was used as the template for PCR. Cloverleaf structures of the Val tRNA is shown. The asterisk (*) indicates a A38 band. (A, B) The specific primers used are complementary to the sequence shown in boldface in the cloverleaf structures. Sequencing ladders were produced using the same primer used in cDNA synthesis. The lane P was loaded with primer only. Download FIG S7, PDF file, 0.3 MB. Copyright © 2022 Choi et al. 2022 Choi et al. https://creativecommons.org/licenses/by/4.0/ This content is distributed under the terms of the Creative Commons Attribution 4.0 International license.

### The role of the cryptococcal KEOPS complex as a transcriptional regulator.

**FIG 7:**
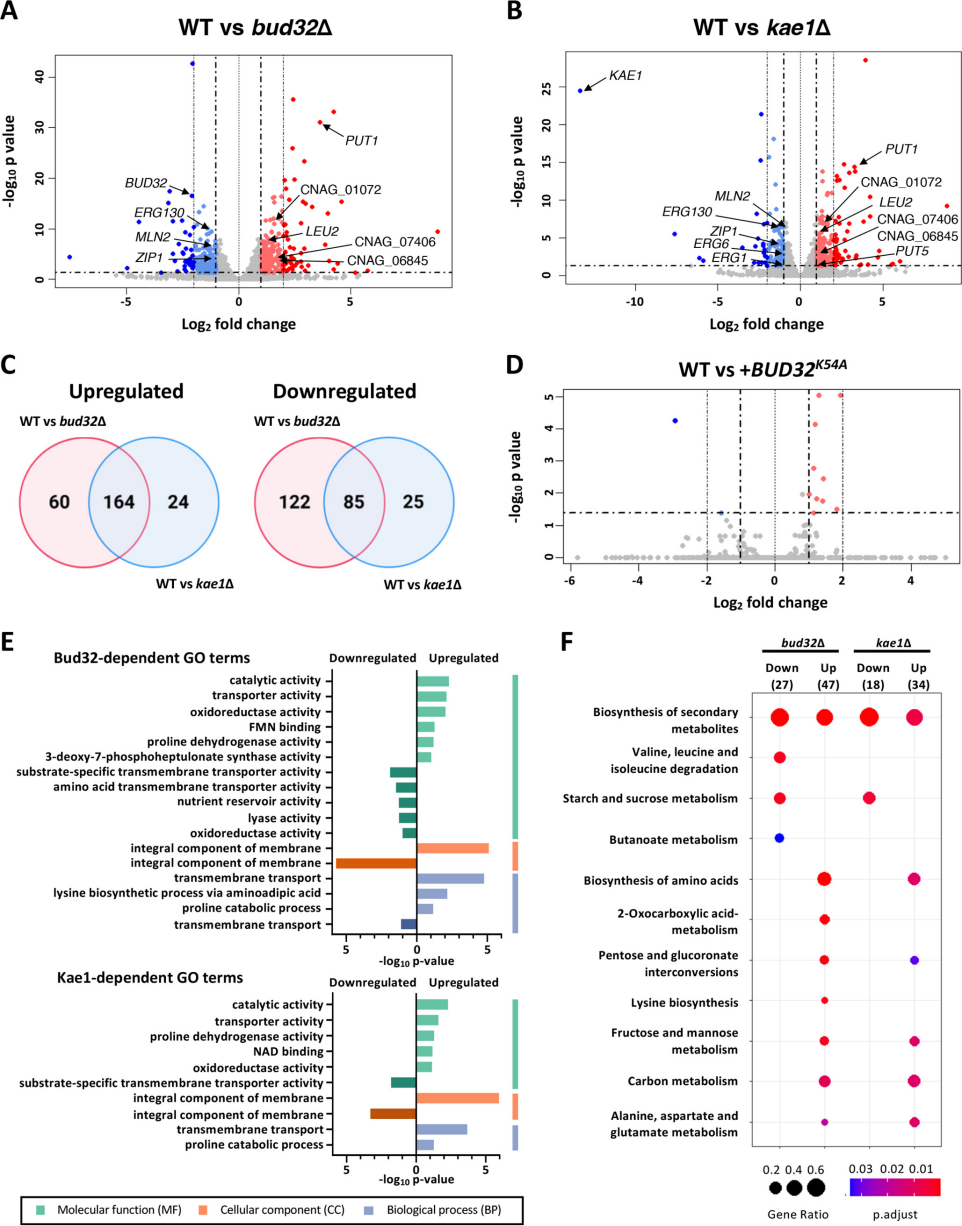
Transcriptome profiles governed by Bud32 and Kae1. (A, B, and D) Volcano plots showing genes whose expressions were significantly upregulated (red-colored) and downregulated (blue-colored) by at least 2-fold in *bud32*Δ (YSB1968) (A), *kae1*Δ (YSB4863) (B), or *bud32*Δ::*BUD32^K54A^* (YSB5874) (D) mutants compared with WT. (C) Venn diagrams showing the number of genes whose expression was significantly upregulated or downregulated by at least 2-fold in the *bud32*Δ and/or *kae1*Δ mutants compared with the WT. (E) DAVID analysis of each functional category in the indicated deletion mutants. Green, orange, and blue bars indicate molecular function (MF), cellular component (CC), and biological process (BP) categories, respectively. (F) Comparative Kyoto Encyclopedia of Genes and Genomes (KEGG) analyses of *bud32*Δ and *kae1*Δ mutants compared with WT. The KEGG analyses were performed using enrichKEGG analysis in Clusterprofiler (https://www.genome.jp/kegg, Clusterprofiler 3.6.0). Gene ratios are indicated by circle sizes, and different colors ranging from blue to red indicates high to low *P*_adjust_ values, respectively.

The yeast KEOPS complex also serves as a transcription factor by interacting with chromatin *in vivo* and can regulate gene expression by recruiting transcriptional coactivators such as SAGA and Mediator (3). To investigate the roles of the CnKEOPS complex as a transcription regulator, we analyzed the transcriptome profiles of wild-type, *bud32*Δ, *bud32*Δ::*BUD32^K54A^*, and *kae1*Δ mutants using RNA-sequencing (RNA-seq) (Fig. 7). The principal-component analysis of the RNA-seq data revealed that the transcriptome profiles of *bud32*Δ and *kae1*Δ mutants were clustered together, but distinct from those of wild type and *bud32*Δ::*BUD32^K54A^* mutant (see Fig. S8A). Deletion of *BUD32* significantly altered the basal expression levels of 431 genes (>2-fold cutoff; *P* < 0.05; 207 downregulated and 224 upregulated genes) (Fig. 7A). Similarly, deletion of *KAE1* significantly changed the basal expression levels of 298 genes (>2-fold cutoff; *P* < 0.05; 110 downregulated and 188 upregulated genes) (Fig. 7B). Notably, the transcriptome profile of the *bud32*Δ mutant was highly similar to that of the *kae1*Δ mutant (Fig. 7C; Fig. S8B and Data Set S2), indicating that Bud32 and Kae1 constitutes a transcriptional regulator complex. In contrast, a lack of the *BUD32* kinase activity affected only a small number of genes (2 downregulated and 10 upregulated genes) (Fig. 7D), suggesting that the Bud32 kinase activity is not critical for the transcriptional regulator activity.

10.1128/mbio.02944-22.8 FIG S8 Transcriptome analysis of *bud32*Δ, *kae1*Δ, and *bud32*Δ::*BUD32^K54A^* strains. (A, B) The PCA (principal component analysis) (A) and heatmap plots (B) were illustrated using DeBrowser v1.22.4. Both PCA and heatmap analyses indicate that transcriptome profiles of wild type and *bud32*Δ::*BUD32^K54A^*(YSB5874) and those of *bud32*Δ (YSB1968) and the *kae1*Δ (YSB4863) were clustered. Download FIG S8, PDF file, 0.1 MB. Copyright © 2022 Choi et al. 2022 Choi et al. https://creativecommons.org/licenses/by/4.0/ This content is distributed under the terms of the Creative Commons Attribution 4.0 International license.

10.1128/mbio.02944-22.10 DATA SET S2 RNA-seq fold-change scores of FPKM. Download Data Set S2, XLSX file, 1.8 MB. Copyright © 2022 Choi et al. 2022 Choi et al. https://creativecommons.org/licenses/by/4.0/ This content is distributed under the terms of the Creative Commons Attribution 4.0 International license.

In agreement with our finding that the KEOPS complex is critical for the cryptococcal cell growth (Fig. 1B), differentially expressed gene (DEG) analysis revealed that many Bud32- and Kae1-dependent genes were related to carbon and nitrogen metabolism, amino acid metabolism, and transporter activity (Fig. 7E and F). Bud32 and Kae1 appeared to regulate proline and leucine metabolism genes, such as *PUT1*, *PUT5*, *LEU2*, and amidase gene (CNAG_01072). Correspondingly, the expression of several transporter genes appeared to be regulated by both Bud32 and Kae1, which might be a compensatory response to the altered nutrient utilization in the KEOPS mutants. Most notably, the deletion of *BUD32* or *KAE1* downregulated ergosterol biosynthesis-related genes, such as *ERG130*, *ERG1*, and *ERG6* (Data Set S2). The transcription factors regulated by Kae1 and Bud32 include *STB4* and *MLN2*. Supporting the role of the KEOPS complex in the sexual reproduction of C. neoformans, several pheromones and mating-related genes (CNAG_07406, CNAG_06845, and *ZIP1*) were commonly upregulated by Bud32 and Kae1. Taken together, the KEOPS complex serves as a major transcriptional regulator in C. neoformans and regulates genes involved in carbon and nitrogen metabolism, amino acid metabolism, sterol biosynthesis, pheromone response and mating, and transcription factor regulation.

### The role of cryptococcal KEOPS complex as a major regulator of sterol biosynthesis-related genes.

**FIG 8:**
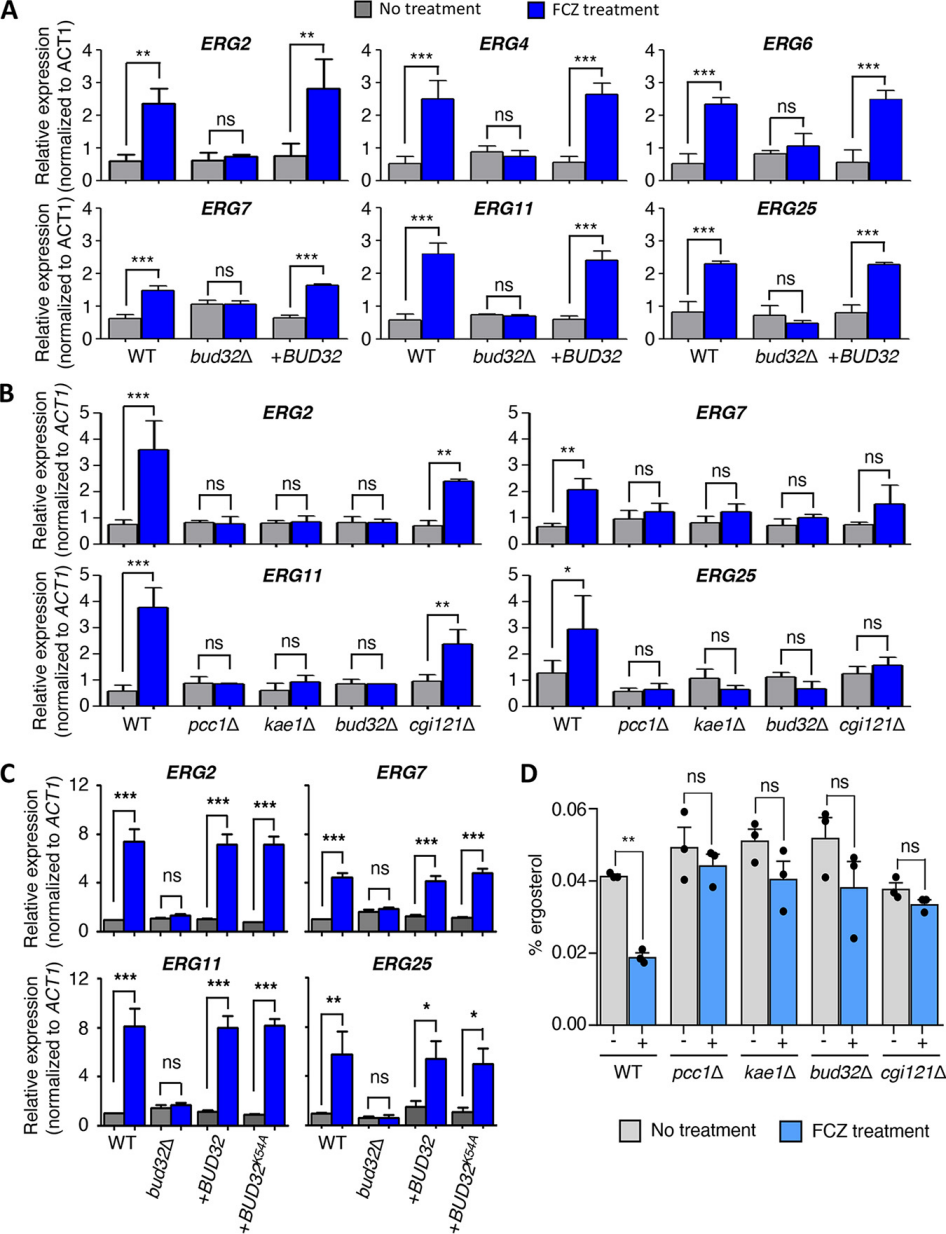
The role of the cryptococcal KEOPS in regulation of ergosterol biosynthesis-related genes. (A–C) Quantitative reverse transcription-PCR (qRT-PCR) analysis for monitoring expression levels of ergosterol biosynthesis-related genes (*ERG2*, *ERG4*, *ERG6*, *ERG7*, *ERG11*, or *ERG25*) after fluconazole treatment. Each strain (WT [H99], *bud32*Δ [YSB1968], *bud32*Δ::*BUD32* [YSB4662], *bud32*Δ::*BUD32^K54A^* [YSB5874], *pcc1*Δ [YSB4930], *kae1*Δ [YSB4863], or *cgi121*Δ [YSB5037]) grown until the midlogarithmic phage was treated with 10 μg/mL of fluconazole (FCZ) for 90 min. qRT-PCR was performed using total RNA isolated from strains under basal and fluconazole-treated conditions. Expression levels of each *ERG* gene were normalized using *ACT1* expression levels. Data were collected from three biological replicates with three technological replicates. (D) The cellular ergosterol contents of WT, *pcc1*Δ, *kae1*Δ, *bud32*Δ, and *cgi121*Δ strains upon fluconazole treatment. Each strain was treated with fluconazole (10 μg/mL) at 30°C for 24 h. Then, the ergosterol contents were measured as described in Materials and Methods. Statical significance in (A–D) was calculated using one-way ANOVA with Bonferroni’s correction (*, *P* = 0.01–0.05; **, *P* = 0.001–0.01; NS, not significant). Error bars represent standard error of the mean.

The transcriptome analysis revealed that several ergosterol biosynthesis-related genes were upregulated by Bud32 and Kae1, suggesting that the CnKEOPS complex plays a central role in ergosterol biosynthesis-related gene expression. Therefore, we investigated the regulatory role of the KEOPS complex in ergosterol biosynthesis-related gene expression in response to azole treatment. Fluconazole treatment generally increased the induction of ergosterol biosynthesis-related genes, such as *ERG2*, *ERG4*, *ERG6*, *ERG7*, *ERG11*, and *ERG25* in the wild-type strain but not in the *bud32*Δ mutant (Fig. 8A). Reintegration of *BUD32* restored the normal induction of the *ERG* genes in the *bud32*Δ mutant (Fig. 8A). Therefore, it is evident that Bud32 is critical for regulation of sterol biosynthesis-related genes. Similarly, deletion of *PCC1* and *KAE1* abolished the induction of these genes under sterol depletion by fluconazole treatment, whereas deletion of *CGI121* partially reduced their induction (Fig. 8B). Notably, the kinase-dead allele of *BUD32* (*BUD32^K54A^*) also restored the normal induction of the *ERG* genes in the *bud32*Δ mutant (Fig. 8C), indicating that the kinase activity of Bud32 is unessential for the regulation of sterol biosynthesis-related genes.

Despite the lack of azole-mediated induction of ergosterol biosynthesis-related genes in the KEOPS mutants, they showed increased resistance to fluconazole (Fig. 2A). Induction of ergosterol biosynthesis-related genes upon sterol depletion by fluconazole treatment is a compensatory mechanism to maintain intracellular sterol homeostasis. Therefore, we hypothesized that deletion of the KEOPS complex could inhibit sterol depletion by azole treatment, resulting in incomplete induction of ergosterol biosynthesis-related genes by fluconazole treatment. To address this possibility, we measured intracellular ergosterol contents in the wild type and KEOPS mutant strains with or without fluconazole treatment. Fluconazole treatment significantly reduced the ergosterol content in the wild-type strain but not in the KEOPS mutants, supporting our hypothesis (Fig. 8D). Taken together, the KEOPS complex is involved in the regulation of fungal sterol content and subsequent ergosterol biosynthesis-related gene expression.

## DISCUSSION

**FIG 9:**
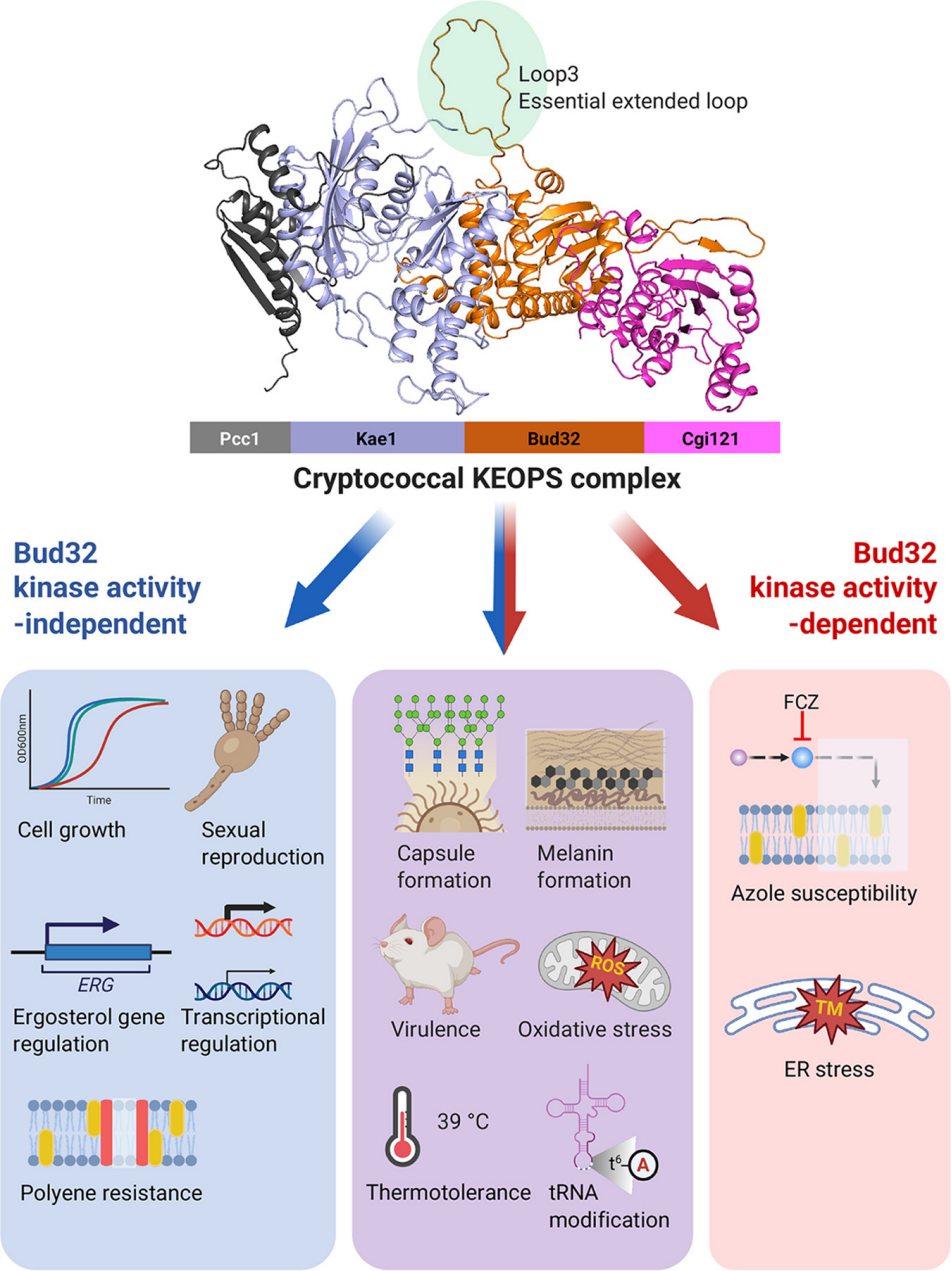
A summary of the biological functions of the cryptococcal KEOPS complex. The cryptococcal KEOPS complex appears to consist of a linear arrangement of Pcc1-Kae1-Bud32-Cgi121. The KEOPS complex plays diverse pathobiological functions in C. neoformans in both Bud32 kinase activity-dependent (red arrows) and -independent (blue arrows) manners. Notably, the unique extended loop structure in CnBud32 is absolutely required for all the functions of the cryptococcal KEOPS complex.

In this study, we identified and functionally characterized the fungal KEOPS complex, consisting of Pcc1, Kae1, Bud32, and Cgi121 in C. neoformans. This evolutionarily conserved protein complex has been structurally and functionally characterized in the model budding yeast S. cerevisiae but not in fungal pathogens. An ortholog of Gon7, an additional subunit of the yeast KEOPS complex, has not been identified in C. neoformans. Based on our bioinformatics analysis, several fungal species do not contain Gon7 orthologs, suggesting that Gon7 is not an evolutionarily conserved KEOPS component at least in fungi. In humans, C14ORF142 has been recently identified as the ortholog of yeast Gon7 (24). Here, we demonstrate that the KEOPS complex plays pivotal roles in growth and cell cycle control, differentiation, stress responses, tRNA modification, transcriptional regulation, and sterol biosynthesis in C. neoformans. Therefore, disruption of any one of KEOPS components significantly impacts the virulence of C. neoformans (summarized in Fig. 9).

A previous structural and functional analysis of the yeast KEOPS complex revealed that it consists of a linear arrangement of Gon7-Pcc1-Kae1-Bud32-Cgi121 (10, 22). Supporting this, we also observed physical interactions between each pair of the CnKEOPS components: Pcc1-Kae1 and Kae1-Bud32. The CnKEOPS complex structure predicted using AlphaFold2 is also like the yeast Pcc1-Kae1-Bud32-Cgi121 structure. Among these components, Pcc1, Kae1, and Bud32 appeared to be the central components of the CnKEOPS complex, whereas Cgi121 likely serves as an auxiliary component because the *pcc1*Δ, *kae1*Δ, and *bud32*Δ mutant phenotypes were indistinguishable, but the *cgi121*Δ mutant exhibited intermediate phenotypes. This is in good agreement with the previous finding that Pcc1, Kae1, and Bud32 are required for the archaeal KEOPS function, but Cgi121 only increases its efficiency (25). In several archaea, Kae1 and Bud32 are often fused as a single protein (26, 27). However, one notable difference we observed is the presence of the extended internal loop 3 of Bud32 in C. neoformans. We found that the extended internal loop 3 is critical for the function of Bud32. According to the predicted complex structure, the loop 3 region of C. neoformans is adjacent to the Kae1 protein, suggesting the possible interaction between them; however, the region would be flexible and has very low model confidence according to the structure prediction result (the IDDT score is less than 30). It was previously reported that Bud32 in S. cerevisiae shows kinase activity after forming a functional unit with Kae1, which has ATPase activity (22, 25). The fact that CnBud32 is a kinase with a phosphotransferase domain, and Kae1 has a substrate binding site and an ATPase domain supports the possibility that the two proteins act as a unit in C. neoformans.

The yeast KEOPS complex has three major biological functions: (i) tRNA modification, (ii) transcriptional regulation, and (iii) telomere regulation. We also found that the first two roles are conserved in the CnKEOPS complex. All four KEOPS components, Pcc1, Kae1, Bud32, and Cgi121, were involved in catalyzing t^6^A modification at position 37 of ANN codon-recognizing tRNAs, such as Ile AAU. We found that the kinase activity of Bud32 is required, but not essential, for the t^6^A modification in C. neoformans, consistent with the finding in S. cerevisiae where the kinase activity of Bud32 is partially required for the t^6^A modification (28). In addition, KEOPS evidently works as a transcriptional regulator in C. neoformans. Our RNA-seq-based transcriptomic analysis revealed that deletion of *BUD32* and *KAE1* led to altered expression of several hundreds of genes, particularly those involved in carbon and nitrogen metabolism, amino acid metabolism, pheromone response/mating, and transport. The previous DNA microarray-based transcriptome analysis in S. cerevisiae also reported that genes involved in carbohydrate and amino acid biosynthesis and pheromone response are regulated by the yeast KEOPS complex (3, 29), suggesting that these KEOPS functions are conserved in fungi.

Despite the conserved roles of the CnKEOPS complex in tRNA modification and transcriptional regulation, its role in telomere regulation remains unclear. In S. cerevisiae, the KEOPS complex regulates telomerase recruitment or telomerase activity at double-strand breaks (2). Deleting the KEOPS complex leads to short telomeres and prevents the addition of new telomeres to DNA double-strand breaks (2). However, when we monitored the subtelomeric regions of chromosomes 1, 4, and 5 of C. neoformans using Southern blot analysis, we observed no difference in telomere length between the wild type and KEOPS mutant strains (data not shown). The recent RNA-seq analysis revealed that the genes in the subtelomeric region are upregulated in the S. cerevisiae KEOPS mutants (30). However, based on our RNA-seq data, we could not find any significant changes in the expression level of genes in the subtelomeric region of KEOPS mutants. Therefore, the potential role of the CnKEOPS complex in telomere silencing should be further investigated.

Besides the conserved roles, this is the first study to report the role of the fungal KEOPS complex in the regulation of sterol biosynthesis-related genes. Fluconazole-mediated induction of *ERG2*, *ERG7*, *ERG11*, and *ERG25* expression was completely dependent on Pcc1, Kae1, and Bud32 but only partially on Cgi121. Notably, the kinase activity of Bud32 appeared to be dispensable for this function. However, our data implied that the CnKEOPS complex indirectly affects ergosterol biosynthesis-related gene expression by affecting cellular sterol content. If the KEOPS complex is directly involved in the induction of ergosterol biosynthesis-related genes, sterol contents upon azole treatment should be lower in the KEOPS mutants than in the wild-type strain. However, we found that cellular sterol contents in the KEOPS mutants were maintained even after fluconazole treatment, possibly explaining the greater fluconazole resistance of KEOPS mutants than the wild-type strain. KEOPS complex might regulate genes involved in azole uptake. Supporting this, our RNA-seq analysis showed that several transporter and transmembrane protein genes are regulated by the KEOPS complex. Whether such transporters are involved in fluconazole uptake remains to be investigated.

Our data clearly demonstrated that the fungal KEOPS complex is critical for the pathogenicity of C. neoformans, which is not surprising considering its critical role in the growth and virulence factor production of C. neoformans. Interestingly, the kinase-dead *BUD32^K54A^* strain exhibited normal growth and significantly restored virulence factor production but with highly attenuated virulence. These data suggest that the kinase activity of Bud32 is also required for fungal pathogenicity. Supporting this, Bud32 is known to phosphorylate and/or interact with other substrates apart from the KEOPS complex (7, 9, 31). In S. cerevisiae, Bud32 phosphorylates Ser134 of the monothiol glutaredoxin Grx4p and is itself regulated by the Sch9 kinase (32). Grx4 interacts with Cir1, a key regulator for iron uptake and virulence of C. neoformans and essential for its pathogenicity (33). However, the functional and mechanistic connection between Sch9, Bud32, and Grx4 is not clear in C. neoformans due to the following reasons. First, although the *grx4*Δ mutants exhibit severe defects in growth at 30°C and 37°C and the production of capsule and melanin (33), our result demonstrated that the kinase-dead *BUD32^K54A^* strain showed normal growth and significantly restored virulence factor production. Therefore, other Bud32 substrates could be responsible for the attenuated virulence of the kinase-dead *BUD32^K54A^* strain. Second, although the Ser258 residue of the yeast Bud32 is phosphorylated by Sch9 (32), the corresponding residue is not conserved in CnBud32. Therefore, the KEOPS-independent Bud32 substrates and the potential Sch9-Bud32-Grx3 signaling cascade in C. neoformans remain to be elucidated. However, we also cannot exclude the possibility that the *BUD32^K54A^* allele may confer some residual kinase activity at high concentrations of ATP, thus partly restoring certain phenotypes of the *bud32*Δ mutant in C. neoformans.

There is a compelling possibility that the KEOPS complex could be exploited as potential antifungal drug targets. The fungal KEOPS components could be targeted by inhibiting their enzymatic activity (e.g., the kinase activity of Bud32), structural integrity, or protein-protein interactions. In C. neoformans, for example, our data suggest that perturbation of the KEOPS complex could not only reduce the viability and virulence of the pathogen within the host, but also synergize with the antifungal activity of amphotericin B, which is widely used for the treatment of cryptococcal infection. However, it will be challenging to specifically target the fungal KEOPS complex, but not the human KEOPS complex, due to their structural and functional similarities. In the case of cryptococcal infections, the unique but functionally critical, extended loop of CnBud32 could be targeted for developing anticryptococcal-specific drugs. Based on these findings, protein structure-based chemical design and development targeting the fungal KEOPS complex is warranted in future studies.

## MATERIALS AND METHODS

### Ethics statement.

Animal care and all experiments were conducted in accordance with the ethical guidelines of the Institutional Animal Care and Use Committee (IACUC) of National Taiwan University (NTU). The NTU IACUC approved all the vertebrate studies (NTU107-EL-00111).

### Construction of KEOPS mutant strains.

The KEOPS complex mutant strains were constructed in the C. neoformans serotype A H99S strain background. Gene disruption cassettes containing the nourseothricin-resistance marker (nourseothricin acetyltransferase; *NAT*) with indicated signature-tagged sequences were generated using conventional overlap PCR or *NAT* split marker/double-joint (DJ) PCR strategies, as previously reported (34, 35). For gene deletion of *KAE1*, *PCC1*, and *CGI121*, *NAT* signature-tagged sequences (numbers 212, 43, and 184) were used in gene disruption cassettes, respectively. All primers used in this study are listed in Data Set S1. In the first round of PCR, the 5′- and 3′-flanking regions for the targeted genes (*KAE1*, *PCC1*, and *CGI121*) were amplified with primer pairs L1/L2 and R1/R2, respectively, using H99S genomic DNA as a template. For the overlap PCR, the whole *NAT* marker was amplified with the primers M13Fe (M13 forward extended) and M13Re (M13 reverse extended) using a pNAT-STM plasmid containing the *NAT* gene with each unique signature-tagged sequence. For the split marker/DJ-PCR, the split 5′- and 3′-regions of the *NAT* marker were amplified with primer pairs M13Fe/SM2 and M13Re/SM1, respectively, with the plasmid pNAT-STM numbers 212, 43, or 184. In the second round of overlap PCR, the gene disruption cassettes were amplified with primers L1 and R2 by combining the first round PCR products as the templates. In the second round of split marker/DJ-PCR, the 5′- and 3′-regions of *NAT-*split gene disruption cassettes were amplified with primer pairs L1/SM2 and R2/SM1, respectively, by combining the corresponding first-round PCR products as the templates. For biolistic transformation, the H99S strain was cultured overnight at 30°C in 50 mL yeast extract-peptone-dextrose (YPD) medium, pelleted, and resuspended in 5 mL of distilled water. Approximately 200 μL of the cell suspension was spread on YPD solid medium containing 1 M sorbitol and further incubated at 30°C for 3 h. The PCR-amplified gene disruption cassettes were coated onto 600 μg of 0.6 μm gold microcarrier beads (Bio-Rad) and biolistically introduced into the cells using a particle delivery system (PDS-100, Bio-Rad). The transformed cells were further incubated at 30°C to recover cell membrane integrity, scraped after 4 h, and transferred to the selection medium (YPD solid plate containing 100 μg mL^−1^ nourseothricin; YPD+NAT). Stable *NAT*-positive transformants were selected through more than two passages on the YPD+NAT plates. All *NAT*-positive strains were first screened using diagnostic PCR with each screening primer listed in Data Set S1. To verify accurate gene deletion and the absence of any ectopic integration of each gene disruption cassette, Southern blot analysis was performed for all the mutants (Fig. S1D–F). To validate a mutant phenotype and exclude any unlinked mutational effects, we constructed more than two independent deletion strains for each mutant.

10.1128/mbio.02944-22.9 DATA SET S1 C. neoformans strains and primers used in this study. Download Data Set S1, XLSX file, 0.01 MB. Copyright © 2022 Choi et al. 2022 Choi et al. https://creativecommons.org/licenses/by/4.0/ This content is distributed under the terms of the Creative Commons Attribution 4.0 International license.

### Construction of complemented and epitope/fluorescent protein-tagged strains.

To generate complemented strains for the *bud32*Δ mutant, a DNA fragment containing the promoter, terminator, and open reading frame (ORF) of *BUD32* was amplified using primer pair B7763/B7764, cloned into a pTOP-V2 vector to generate the plasmid pTOP-BUD32, and confirmed using DNA sequencing. For *bud32*Δ*::BUD32* complemented strains, the *BUD32* insert was subcloned into pJAF12 containing the neomycin/G418-resistant marker (*NEO*) to generate pJAF12-BUD32. For *bud32*Δ*::BUD32*-*4xFLAG* strains, the *BUD32* insert was subcloned into pJAF12 (NEO^r^) containing a *4xFLAG* sequence to produce pNEO-BUD32-4xFLAG (36). For *bud32*Δ*::BUD32-mCherry* strains, the *BUD32* insert was subcloned into pNEO plasmid containing mCherry to produce pNEO_BUD32-mCherryht. Each ClaI-digested linearized plasmid was biolistically introduced into the *bud32*Δ mutants (YSB1968). To construct other mCherry-tagged complemented strains, each ORF region without a stop codon and the respective promoter of *PCC1*, *KAE1*, and *CGI121* were amplified and cloned into the pNEO-mCherryht plasmid using Gibson Assembly master mix kit (New England BioLabs). The plasmids pNEO-PCC1-mCherryht, pNEO-KAE1-mCherryht, and pNEO-CGI121-mCherryht were linearized using restriction digestion with AfeI, BsmI, and AflII, respectively, and introduced in the *pcc1*Δ (YSB4930), *kae1*Δ (YSB4863), and *cgi121*Δ (YSB5037) through biolistic transformation. The targeted integration of each linearized plasmid into the corresponding native locus was confirmed with a diagnostic PCR.

To construct strains containing *KAE1-4xFLAG* or *PCC1-6xHA*, each cassette for C-terminal chromosomal tagging was generated using a split marker/double-joint PCR strategy, as previously reported (34, 35). The 3′-ORF regions, 3′-flanking regions of each gene, and plasmids containing the *4xFLAG* or *6xHA* sequence were separately amplified in the first round of PCR. Then the 3′-ORF region and *NAT-*spilt marker for *KAE1-4xFLAG* or the hygromycin B resistance gene (*HYG*; hygromycin B phosphotransferase) split marker for *PCC1-6xHA* were combined. Also, the 3′-flanking region and each dominant selectable split marker were combined in the second round of PCR. The tagging cassettes were delivered into the H99S strains or each tagged strain through biolistic transformation. The genotypes of tagged strains were confirmed using Southern blot.

### Construction of Bud32 kinase-dead mutant and Bud32 containing the short loop region of yeast Bud32.

To construct the kinase-dead *BUD32* (*BUD32^K54A^*) strain, site-directed mutagenesis was conducted as previously described (37). The plasmid containing the *BUD32* allele was amplified with mutagenic primers listed in Data Set S1. The products were digested with DpnI for 2 to 3 h at 37°C. After the reaction, the purified DNA was transformed into E. coli. The plasmid containing the *BUD32^K54A^* allele was confirmed through sequencing and subcloned into pNEO to generate pNEO_BUD32^K54A^. Then, the ClaI-digested linearized pNEO_BUD32^K54A^ was integrated into the *BUD32* native locus. To construct the *bud32*Δ::*BUD32^ScL3^* strain where the extended loop 3 region of CnBud32 was replaced with the short loop region of yeast Bud32, the DNA fragments spanning the promoter and loop3 upstream regions, the loop3 downstream and terminator regions, and the short loop region of yeast Bud32 were amplified using PCR with primers listed in Data Set S1. The first and second PCR products were combined and used as the templates for overlap PCR using primers B7763 and B9316. The second and third PCR products were combined and used as the templates for overlap PCR using primers B9313 and B7764. Finally, two overlap PCR products were combined and used for the third round of PCR with primers B9366 and B9367. The final DNA product was subcloned into pNEO plasmid (pNEO_BUD32^ScL3^), digested with ClaI, and introduced into the *bud32*Δ mutants (YSB1968).

### Imaging cellular localization of mCherry-tagged proteins.

The mCherry-tagged strains were incubated in YPD broth overnight at 30°C and subcultured in fresh YPD liquid media until their optical density at 600 nm (OD_600_) reached 0.8. The cells were fixed with 4% paraformaldehyde containing 3.4% sucrose for 15 min at room temperature. After incubation, the cells were pelleted, washed with 0.1 M potassium phosphate buffer (pH 7.5) containing 1.2 M sorbitol, and then stored in potassium phosphate buffer. For nuclear staining, the cells were incubated with 10 μg/mL of Hoechst 33342 (Thermo Fisher) in the dark for 30 min. After incubation, samples were observed and photographed using differential interference contrast (DIC) and fluorescence microscopy (Nikon Eclipse, Tokyo, Japan) equipped with a digital camera (DS-Qi2).

### Coimmunoprecipitation and Western blot.

4xFLAG- and/or 6xHA-tagged strains were incubated in YPD liquid medium overnight at 30°C, subcultured in fresh YPD broth, further incubated until OD_600_ = 0.8, pelleted, and frozen in liquid nitrogen. The frozen cell pellet was diluted with lysis buffer containing 50 mM Tris-Cl (pH 7.5), 1% sodium deoxycholate, 5 mM sodium pyrophosphate, 0.2 mM sodium orthovanadate, 50 mM NaF, 1% Triton X-100, 0.5 mM phenylmethylsulfonyl fluoride, and 1× protease inhibitor cocktail solution (Calbiochem, USA). The whole-cell lysates were obtained after bead beating. Anti-FLAG antibody (Sigma-Aldrich, F1804) was added to the lysates and incubated overnight at 4°C rotating. Then, Sepharose protein G beads (GE Healthcare Life Sciences) were added, and the mixture was incubated for 6 h at 4°C rotating. The collected Sepharose beads were washed thrice with lysis buffer containing 50 mM NaCl. The protein bound to the beads was eluted with SDS sample buffer (50 mM Tris-Cl, 2% SDS, 10% glycerol, and 0.01% β-mercaptoethanol) and detected with anti-FLAG (Sigma-Aldrich, F1804) and anti-HA (Santa Cruz Biotechnology, sc-7392) antibodies.

### Growth and chemical susceptibility test.

Each strain was grown in YPD broth for 16 h at 30°C, serially diluted 10-fold (dilution factor: 1 to 10^4^), and spotted onto YPD solid medium containing the indicated concentrations of chemical agents to induce environmental stresses. Cells were incubated at 30°C for 1 to 5 days and photographed daily. To analyze the growth rate of KEOPS mutants, the wild-type strain (H99S) and mutants were incubated at 30°C overnight and their cell concentrations were adjusted to OD_600_ = 0.2 in fresh YPD liquid medium. Cells were then incubated at 30°C in a multichannel bioreactor (Biosan Laboratories, Inc., Warren, MI, USA), and OD_600_ was automatically measured for 70 h.

### Mating.

To monitor unilateral mating efficiency, each *MAT*α mutant and wild-type (H99S) strain was mated with the *MAT***a** KN99**a** strain. Each cell was cultured in YPD broth for 16 h, pelleted, and washed twice with phosphate-buffered saline (PBS). The *MAT*α cells (10^7^ cells/mL) were mixed with the equal volume (10^7^ cells/mL) of *MAT***a** KN99**a** cells, spotted on V8 mating media (pH 5), and incubated in the dark for 10 days. Filamentous growth was observed and photographed using DIC microscopy (BX51, Olympus) equipped with a SPOT Insight digital camera (Diagnostic Instrument Inc.).

### Virulence factor production assay.

For the melanin production assay, cells cultured overnight were washed twice with PBS and spotted on Niger seed, dopamine, or epinephrine agar media (1 g l-asparagine, 3 g KH_2_PO_4_, 250 mg MgSO_4_, 1 mg thiamine, 5 μg biotin, and 100 mg L-DOPA or epinephrine hydrochloride per L) containing 0.1% or 0.2% glucose. The cells were incubated at 37°C and photographed for 1 to 3 days. The capsule production assay was performed using two types of capsule-inducing media: FBS (10% of fetal bovine serum and 90% of PBS) agar media and Littman’s agar media. The overnight cultured cells were washed twice with PBS, and 3 μL of cells were spotted on the capsule-inducing media and incubated at 37°C for 2 days. The cells were scraped, resuspended in distilled water, mixed with India ink for visualization, and observed using DIC microscopy. Fifty cells were randomly selected to measure capsule and cell diameter using SPOT Advanced v4.6 software. The capsule thickness was calculated as: total diameter − cell body diameter. Statistical significance was calculated using one-way ANOVA analysis with Bonferroni’s multiple-comparison test.

### Transcriptome analysis using RNA sequencing.

Cells cultured overnight were inoculated in fresh YPD broth at the OD_600_ of 0.2 and further incubated at 30°C until OD_600_ reached 0.8. The cells were pelleted, frozen in liquid nitrogen, and lyophilized overnight. Total RNA was extracted using Easy-BLUE (iNtRON) and purified with a RNeasy minikit (Qiagen). Total RNA was prepared from three independent cultures of each strain. The cDNA library was constructed with the 1 μg of total RNA for each sample using Illumina TruSeq mRNA library kit (Illumina) and sequenced through the Illumina platform. The adapter sequences were trimmed from the sequencing reads using Cutadpat v3.4 with Python v3.7.4. (38). The reference genome sequence of Cryptococcus neoformans H99S and annotation data were downloaded from the NCBI FTP server. The reads were aligned to the C. neoformans H99S genome sequence using Hisat2 v2.2.1 with the Hisat and Bowtie2 algorithm and processed as previously reported (39). Hisat2 was performed with the “-p 30” and “– dta -1” option and other parameters set as default. Aligned reads were converted and then sorted using Samtools v1.9 (40) with the “-Sb -@ 8” option for converting and “-@ 20 –m 2000000000” option for sorting with the other parameters set as default. Transcript assembly and abundance estimation were performed using Stringtie v2.1.1 using the “-p 12” and “-B” option to run the Ballgown analysis (41). The assembled transcripts were merged in a single GTF file, and the relative transcript abundances were calculated via fragments per kilobase of exon per million fragments mapped (FPKM). The FPKM and read count matrix were generated using the R package “isoformswitchanalyzerR” and analyzed using DESeq2. Differentially expressed genes (DEG) analysis was performed using DESeq2 v1.24.0 with default sets with the Ballgown. The volcano plot was illustrated using R v4.1.0, with the cutoff: >2-fold changes with *P* < 0.05. The RNA-seq data were deposited in the Gene Expression Omnibus (GEO) database (accession no. GSE208333), and the differentially expressed genes are listed in Data Set S2.

### Flow cytometry analysis.

Wild type and KEOPS mutants were cultured to an OD_600_ of 0.8, harvested, and washed with PBS. For ethanol fixation, 10^6^ cells in 300 μL of PBS were slowly mixed with 700 μL of 100% EtOH to a final concentration of 70% and incubated for 14 to 16 h at 4°C. Fixed cells were pelleted and serially washed with PBS containing 1% and 0.5% BSA. Then the cells were treated with 200 mg/mL RNase (Thermo Scientific, cat. no. EN0531) for 30 min at 37°C. After centrifugation, the cells were stained with propidium iodide staining buffer (100 μg/mL propidium iodide, 100 mM Tris [pH 7.4], 150 mM NaCl, 1 mM CaCl_2_, 0.5 mM MgCl_2_, 0.1% Nonidet P-40) for 2 h at room temperature in the dark. The cells were washed with PBS and filtered through a strainer. Fluorescence was measured with a BD FACS Aria III. We analyzed 30,000 events for each sample.

### Primer extension assay.

Wild-type (H99S) and KEOPS mutant (*bud32*Δ [YSB1968], *bud32*Δ::*BUD32* [YSB4662], and *bud32*Δ::*BUD32^K54A^* [YSB5874], *pcc1*Δ [YSB4930], *kae1*Δ [YSB4863], and *cgi121*Δ [YSB5037]) cells grown overnight in YPD at 30°C were inoculated into 25 mL fresh YPD medium to an OD_600_ of 0.3 and further grown for 3 h. The cell culture was washed thrice with distilled water and collected into a screw-cap tube after centrifugation. All cell pellets were frozen at −80°C until further use. To isolate total RNA, the TRIzol reagent (Invitrogen) was used following the manufacturer’s instructions. In brief, the frozen cell pellets were resuspended in 1 mL of TRIzol reagent, and 200 μL of chloroform and acidic glass beads were added. Then the cells were disrupted by shaking and incubated at room temperature for 5 min. Following centrifugation, 500 μL of the aqueous phase was collected into a new tube, and 50 μL of sodium acetate (3 M, pH 5.3) was added. RNA was precipitated with 500 μL of ice-cold isopropanol, washed with ice-cold 70% ethanol, and air-dried. The RNA pellet was dissolved in DEPC-treated water. The concentration and purity of total RNA samples were measured using NanoDrop spectrophotometers (NanoDrop 2000). Following this, 3 μg of total RNA was used in primer extension reactions. The Cn_Ile-AAT-R primer (5′-ACGGGATCGAACCGCCGACC -3′) and Cn_Val-AAC-R primer (5′-CGGGCGGGATCGAACCGCCGACC -3′) were labeled at the 5′-end with [γ-^32^P] ATP (6000 Ci/mmol, 10 Ci/L) (PerkinElmer) and T4 polynucleotide kinase (New England Biolabs, Ipswich, MA, USA). RNA and the labeled primers were denatured at 65°C for 5 min and then annealed by cooling to 37°C for 90 min. They were then extended at 42°C for 1 h with 5 units (U) of avian myeloblastosis virus reverse transcriptase (AMV RTase) (New England Biolabs). The products were separated on 15% polyacrylamide gel containing 8 M urea. Sequencing ladders were generated using 5 μg of the PCR product amplified from the cDNA of tRNA^Ile(AAU)^ and tRNA^Val(AAC)^. Images were analyzed in a Bio-Rad phosphorimager using Quantity One software (Bio-Rad Laboratories).

### Quantitative reverse transcription-PCR analysis.

To measure the expression level of ergosterol biosynthesis-related genes (*ERG2*, *ERG4*, *ERG6*, *ERG7*, *ERG11*, and *ERG25*), the wild-type H99S, *bud32*Δ mutant (YSB1968), *bud32*Δ::*BUD32* complemented (YSB5302), *kae1*Δ mutant (YSB4863), *pcc1*Δ mutant (YSB4930), and *cgi121*Δ mutant (YSB5037) strains were incubated in liquid YPD medium at 30°C for 16 h and subcultured into fresh liquid YPD medium. When the cells reached the early logarithmic phase (OD_600_ = 0.8), the culture was divided into two samples: one was treated with fluconazole (FCZ) for 90 min while the other was not. The cell pellets were immediately frozen with liquid nitrogen and then lyophilized. Total RNA was extracted for each strain, and its cDNA was synthesized using RTase (Thermo Scientific). *ERG2*, *ERG4*, *ERG6*, *ERG7*, *ERG11*, *ERG25*, and *ACT1*-specific primer pairs (Data Set S1) were used for qRT-PCR analysis.

### Ergosterol assay.

Ergosterol content was measured as previously described (42). Overnight cultured cells were subcultured with fresh YPD broth with OD_600_ = 0.2. After OD_600_ reached 0.8, the cells were divided into two groups (treated or untreated with 10 μg/mL fluconazole) and further incubated for 24 h. Then the cell pellet was washed with distilled water, frozen in liquid nitrogen, and lyophilized overnight. The dried cell weight was recorded for normalization of ergosterol content. The pellet was added with 5 mL of 25% alcoholic potassium hydroxide (25 g of KOH and 35 mL of sterile distilled water made up to 100 mL with 100% ethanol) and vortexed for 1 min. The sample was transferred to a sterile borosilicate glass screw-cap tube and incubated at 80°C for 1 h. After the sample cooled down at room temperature, 1 mL of sterile water and 3 mL of heptane were added, mixed, and vortexed for 3 min. The 200 μL of the top layer (heptane layer) was harvested and mixed with 800 μL of 100% ethanol. Each 1 mL sample was measured at OD_281.5_ and OD_230_. Ergosterol content was calculated as follows: % ergosterol = ([OD281.5/290] *× F)*/pellet weight − ([OD230nm/518] × *F*)/pellet weight, where *F* is the ethanol dilution factor.

### Cloning, protein expression, and purification of recombinant Bud32.

The full-length *BUD32* gene was amplified using PCR from synthesized DNA (Cosmogene tech.) and cloned into the pVFT3S vector (Korean patent 10-0690230), which contains a tobacco etch virus (TEV) protease recognition site (ENLYFQG) between the N-terminal 6×His-thioredoxin (Trx) and multicloning site. The pVFT3S-BUD32 plasmid was inserted into BL21(DE3) via transformation and grown in a high-salt LB medium until OD_600_ reached 0.6 to 0.8 at 37°C. The temperature was then lowered to 17°C, and overexpression was induced using 0.3 mM 1-thio-β-d-galactopyranoside (IPTG) for 16 h. The 6×His-Trx-Bud32 recombinant protein was purified via metal-affinity chromatography using a Ni-NTA column (GE Healthcare) and digested with TEV protease for 16 h at 4°C. After buffer change through dialysis to remove imidazole, the separated Bud32 and 6×His-Trx were reloaded into the Ni-NTA column, and the flow-through fractions were stored.

### Autophosphorylation assay.

To verify autophosphorylation of recombinant Bud32 protein, recombinant Bud32 (0.2, 0.5, 1, 2, and 4 μg) was assayed by incubation at 37°C for 30 min in a final volume of 20 μL in the presence of 50 mM Tris-HCl, pH 7.5, 10 mM MnCl_2_, and ATP (50 μM or 100 μM). The reaction was stopped by adding SDS-PAGE loading buffer, and samples were subjected to 12% SDS-PAGE at 50 V for 35 min followed by 100 V for 80 min. To visualize the autophosphorylated proteins, the protein gel was stained with Pro-Q Diamond phosphoprotein gel stain (Invitrogen, P33300) and imaged using Chemi-Doc (Bio-Rad).

### *In vitro* kinase assay.

The ATPase/kinase activities of recombinant Bud32 and kinase-dead Bud32 (Bud32^K54A^) proteins were measured using the ADP-Glo kinase assay kit (Promega) according to the instructions provided by the company. The kinase reactions of 4 μg rBud32 or rBud32^K54A^ were performed with (kinase activity) or without 2 μg of α-casein (ATPase activity), and 100 μM ATP in kinase reaction buffer (50 mM Tris-HCl, pH 7.5, 10 mM MnCl_2_) for 30 min at 37°C. Then the kinase reactions were added with 25 μL of ADP-Glo reagent and further incubated for 40 min to stop the kinase reactions and eliminate remaining ATP. Subsequently, 50 μL of kinase detection reagent was added and incubated for 30 min to convert ADP to ATP and introduce luciferase and luciferin to detect ATP. To record luminescence signal, VICTOR X5 multilabel plate reader (PerkinElmer) was used.

### Insect-killing assay.

The virulence assay using an insect host was performed as previously described (43). Galleria mellonella caterpillars (Vanderhorst Wholesale, Inc., Saint Marys, OH, USA) from final larvae weighing 200 to 300 mg were used within 7 d of arrival for the virulence assay. Each C. neoformans strain was incubated in YPD broth at 30°C overnight, washed thrice with PBS, and adjusted to the final concentration of 10^6^ cells/mL in PBS. Four thousand C. neoformans cells were injected into the larvae using repeating dispenser (PB600-1, Hamilton Company, Reno, NV, USA). PBS was injected into larvae as noninfection control. Infected larvae were placed in petri dishes and incubated at 37°C in a humid chamber. The larvae condition was monitored, and their numbers were counted daily. Larvae that turned black and were motionless were considered dead. Pupae during the experiment were censored for statistical analysis.

### Murine infectivity assay.

Seven- to 8-week-old female C57BL/6 mice purchased from BioLasco Inc. (Taiwan) were used for the murine intranasal infection model (*n* = 7 for each group) to test the virulence of the wild-type (H99S), *bud32*Δ (YSB1968), *bud32*Δ::*BUD32* (YS B4662), and *bud32*Δ::*BUD32^K54A^* (YSB5874) strains. Each strain was grown overnight in YPD broth at 30°C and washed twice with PBS. Afterward, cells were resuspended in PBS and diluted to 10^6^ cells/mL. CFU and cell viability were verified by incubating cells on YPD agar at 30°C for 48 h. Mice were anesthetized with isoflurane (Panion & BF Biotech Inc., Taiwan) and infected via intranasal instillation of 50 μL cell suspension (5 × 10^4^ cells). Mice survival was monitored twice daily, and moribund mice (mice that were unable to eat or drink, had a 20% reduction in body weight, or were hunched) were sacrificed. The survival data were plotted using Kaplan-Meier curves and statistically analyzed through log-rank (Mantel-Cox) test using Graph Pad Prism v5.03.

### Data availability.

We will provide any strain and materials used in this study upon request. RNA-seq-based transcriptome profiling data for wild type, and *bud32*Δ, *bud32*Δ::*BUD32^K54A^*, and *kae1*Δ mutants were submitted to Gene Expression Omnibus (accession no. GSE208333).
