# Supplementary figures and images for: Unraveling the Pathobiological Role of the Fungal KEOPS Complex in Cryptococcus neoformans
Source: mBio. 2022 Nov 15;13(6):e02944-22. doi: 10.1128/mbio.02944-22 (PMC9765431; doi:10.1128/mbio.02944-22)

Figure S1

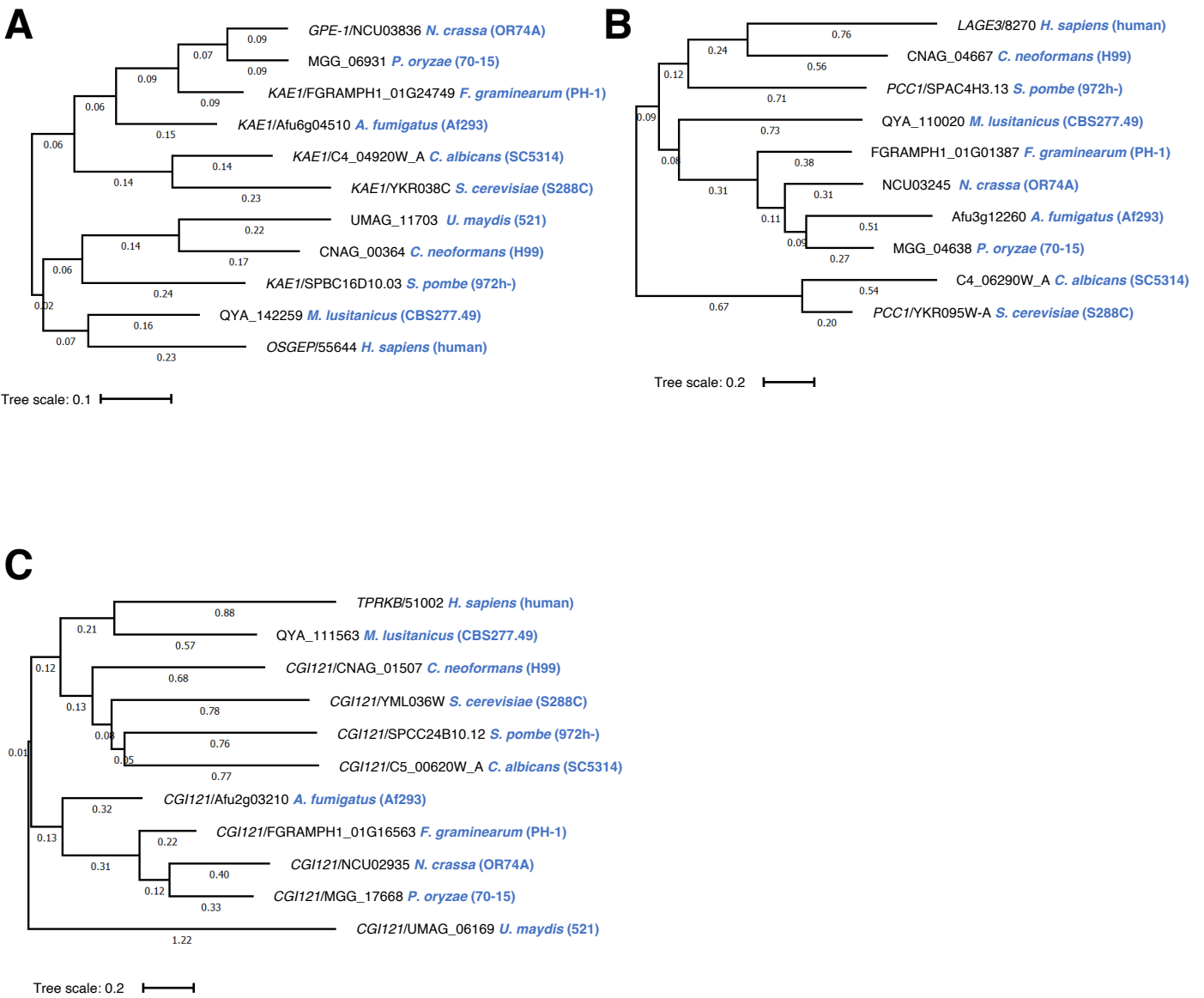

Figure S1

D

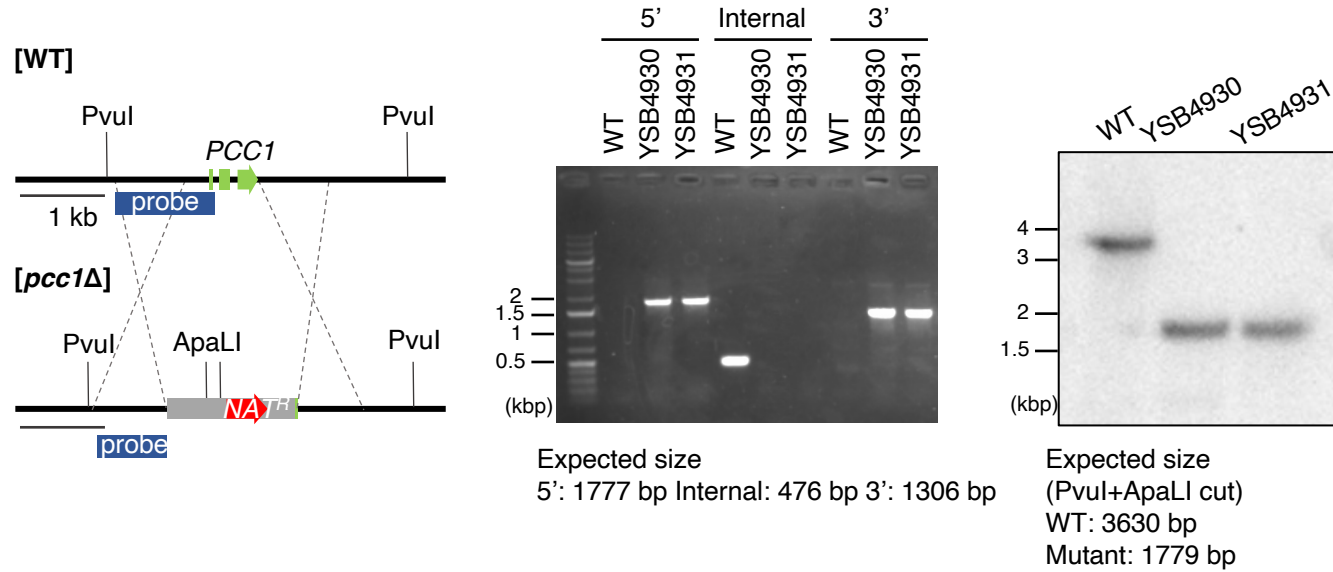

E

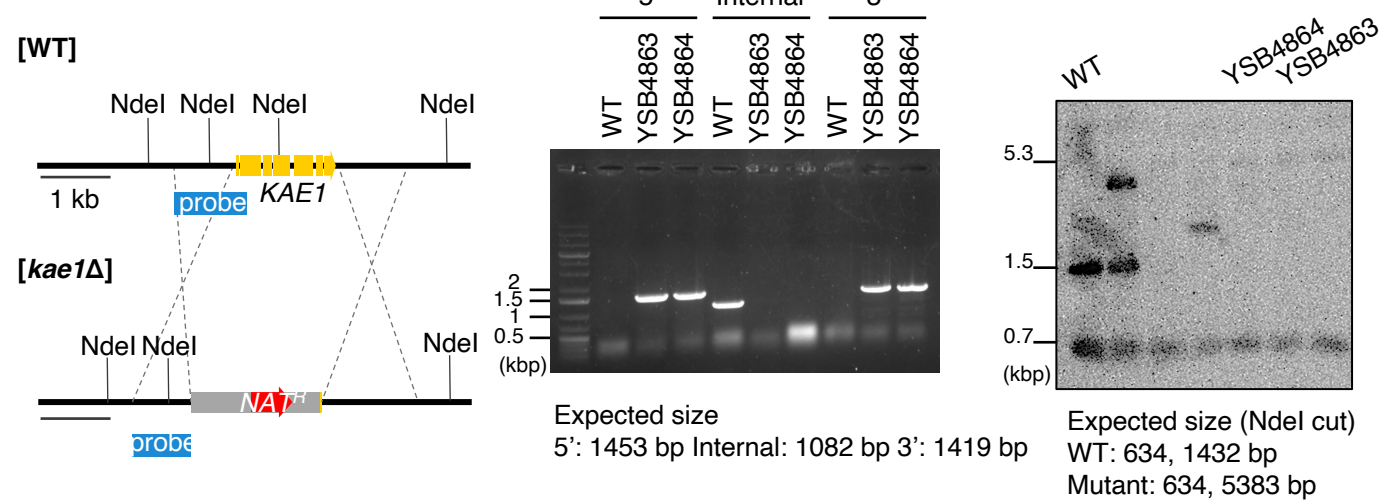

F

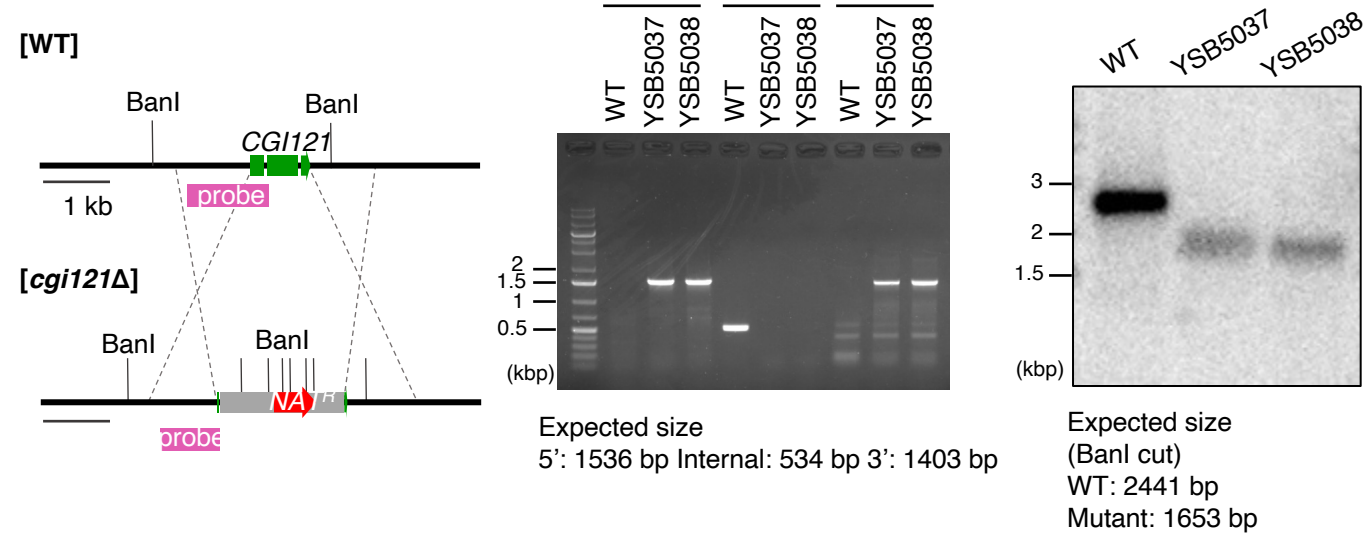

Supplement: FIG S1 [file mbio.02944-22-sf001.pdf]

Figure S2

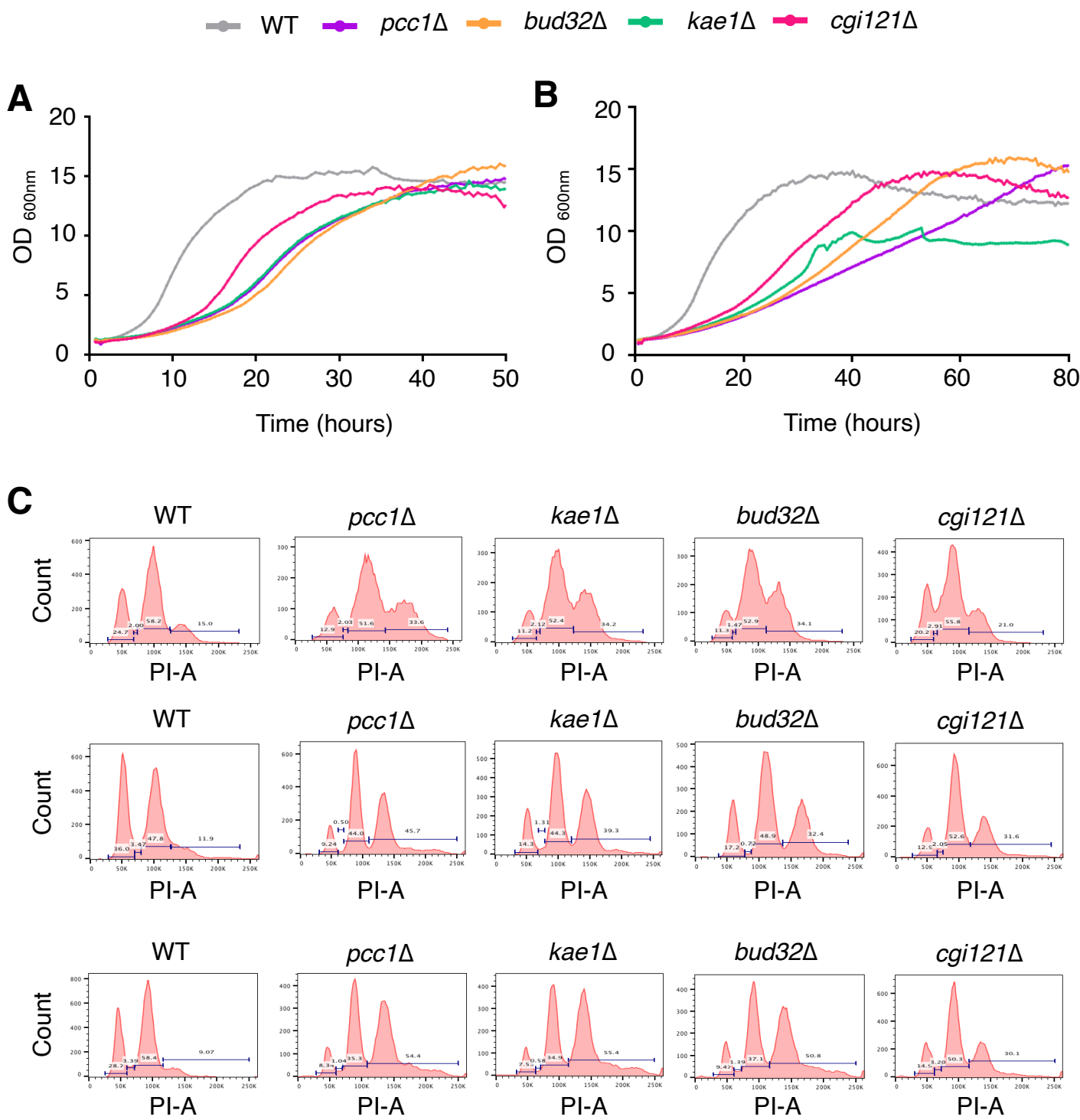

Figure S2

D

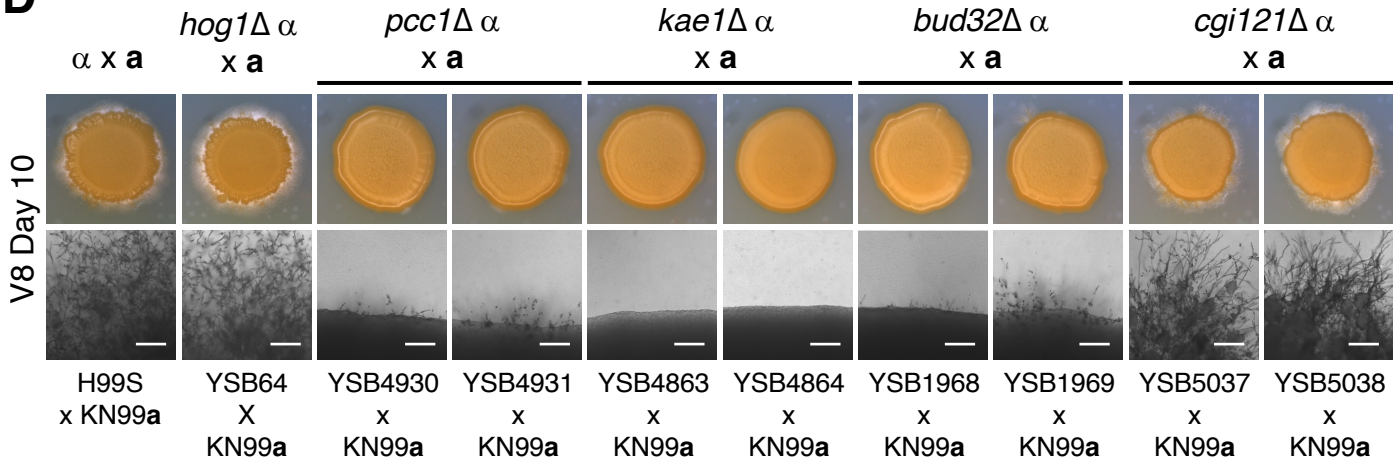

E

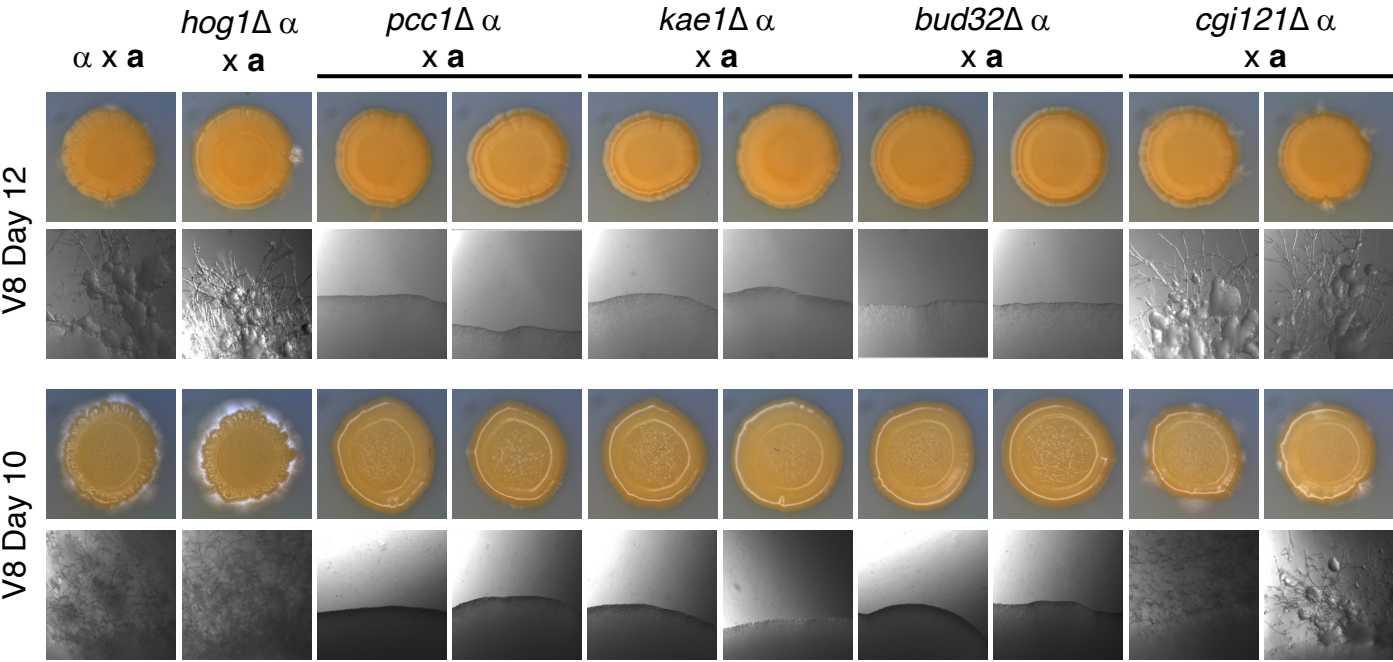

Supplement: FIG S2 [file mbio.02944-22-sf002.pdf]

## Figure S3

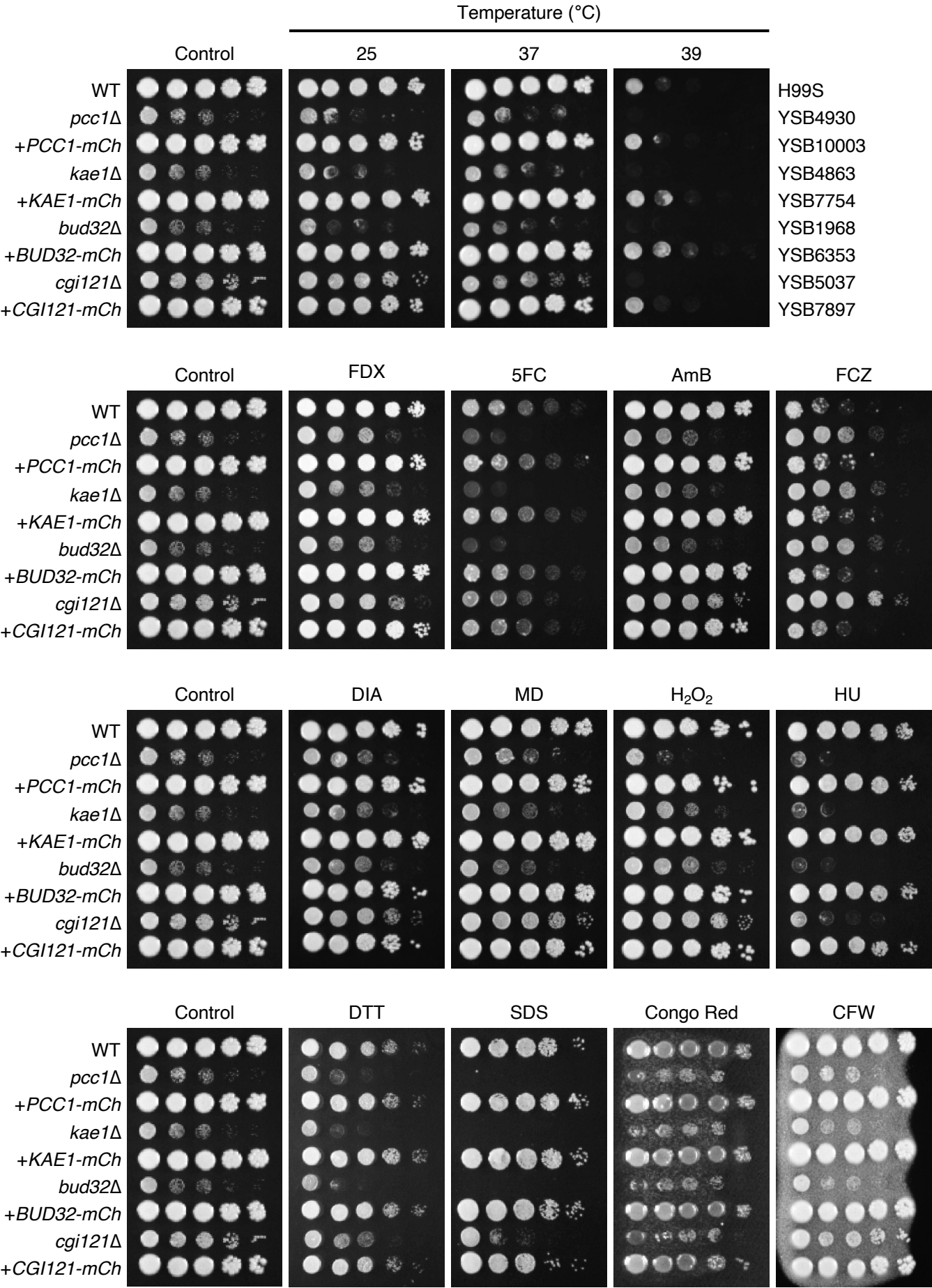

Figure S3

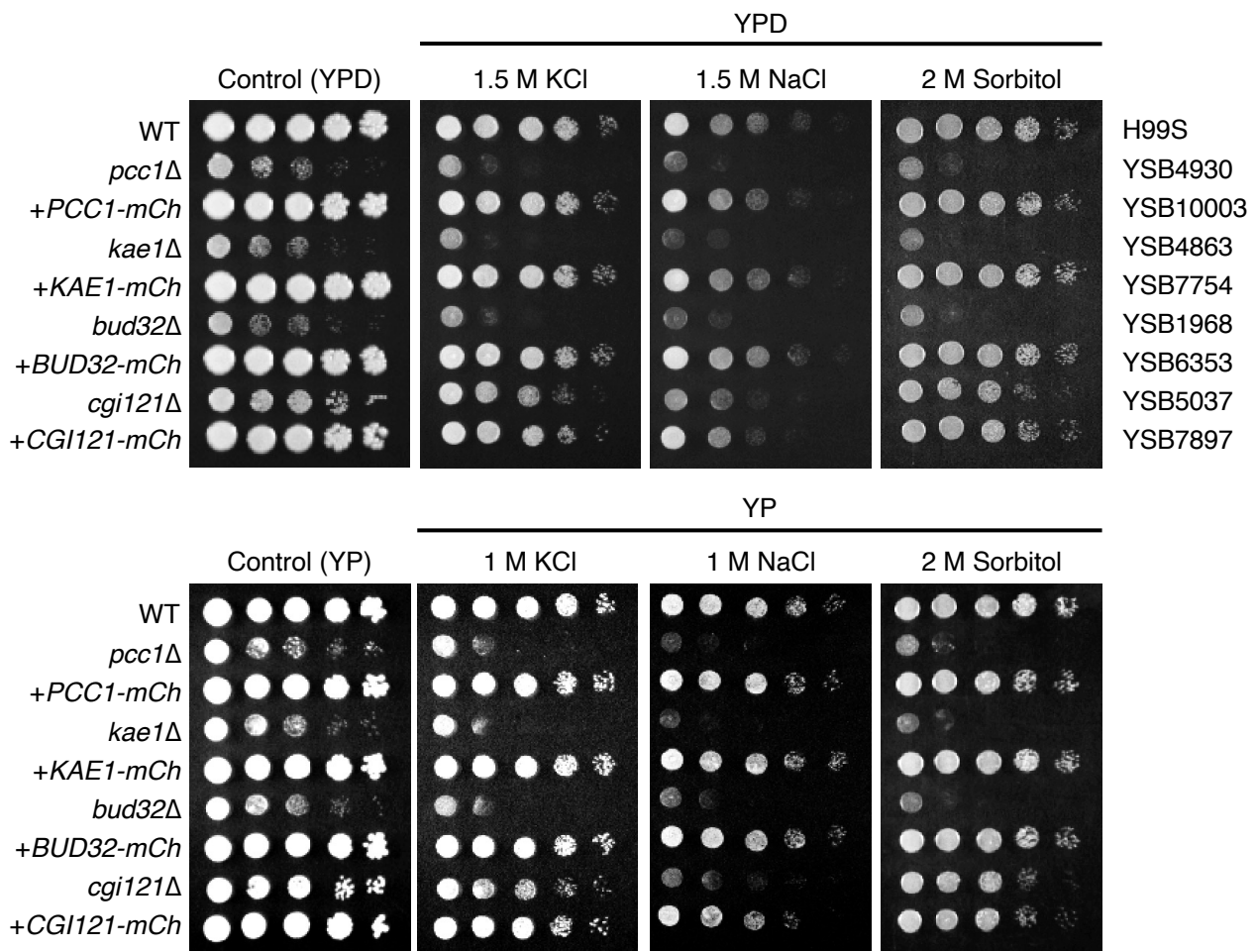

Supplement: FIG S3 [file mbio.02944-22-sf003.pdf]

Figure S4

A

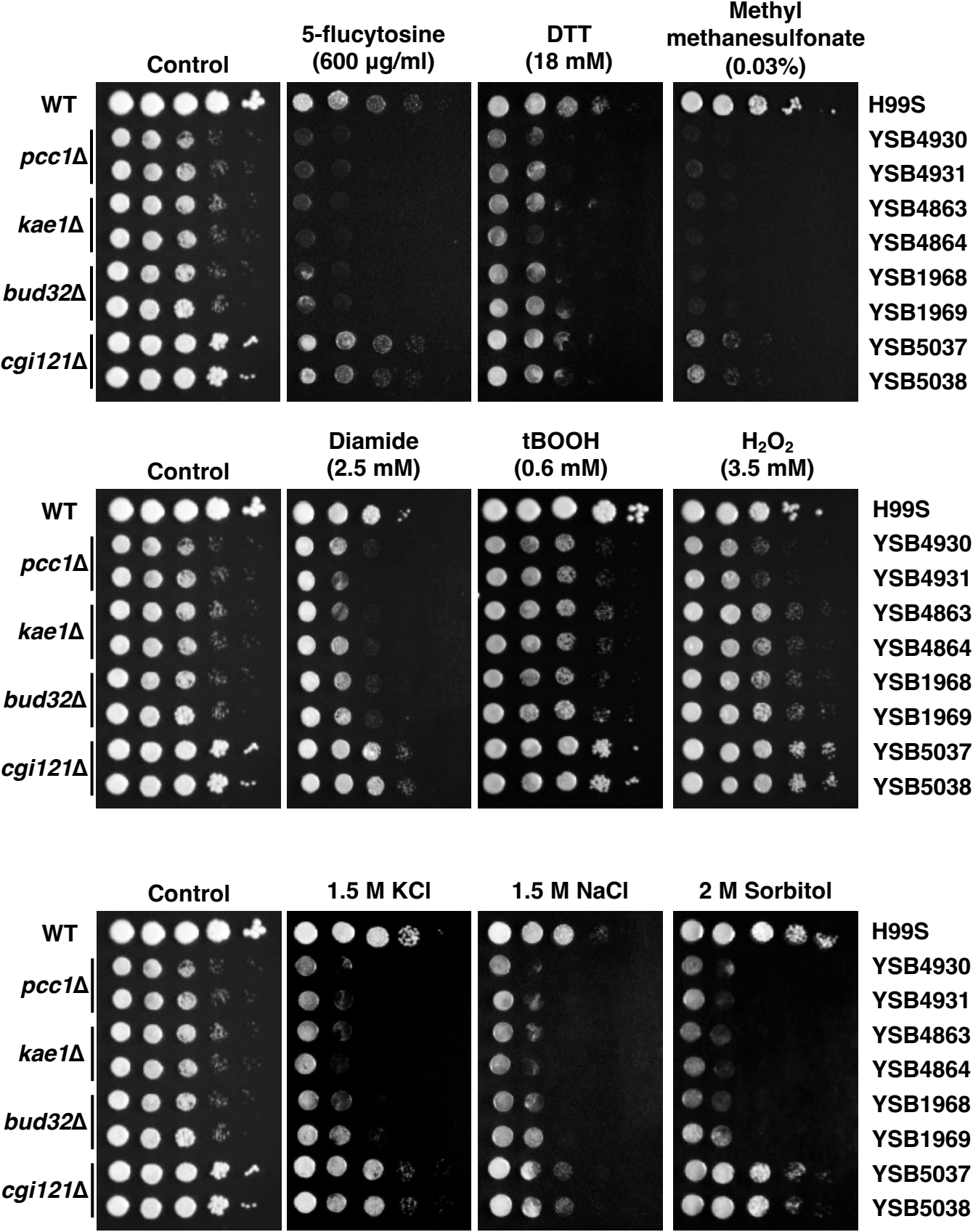

Figure S4

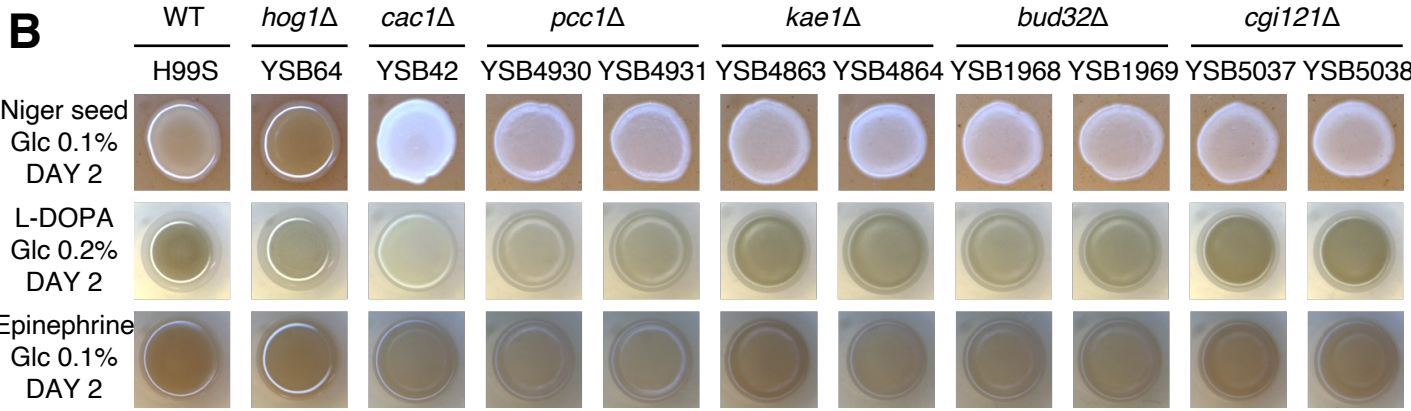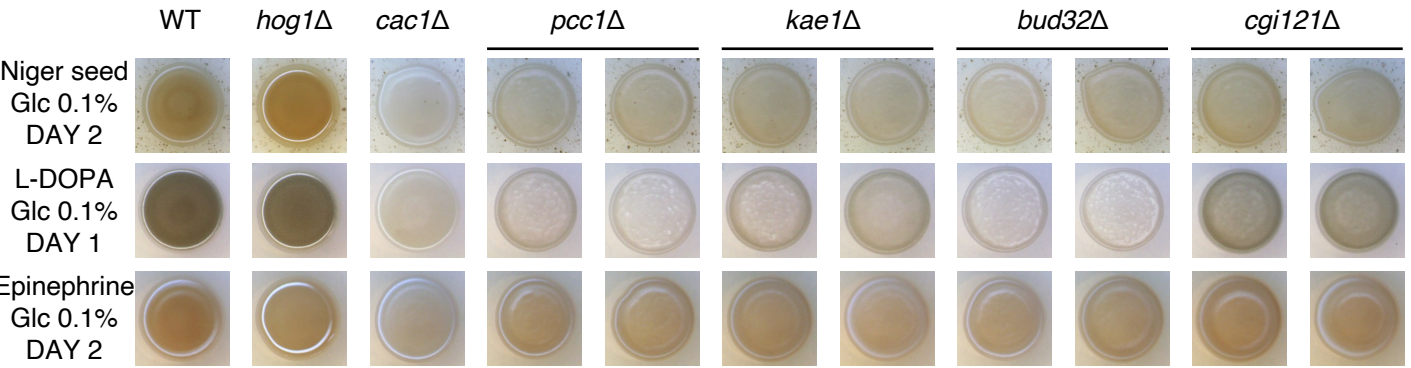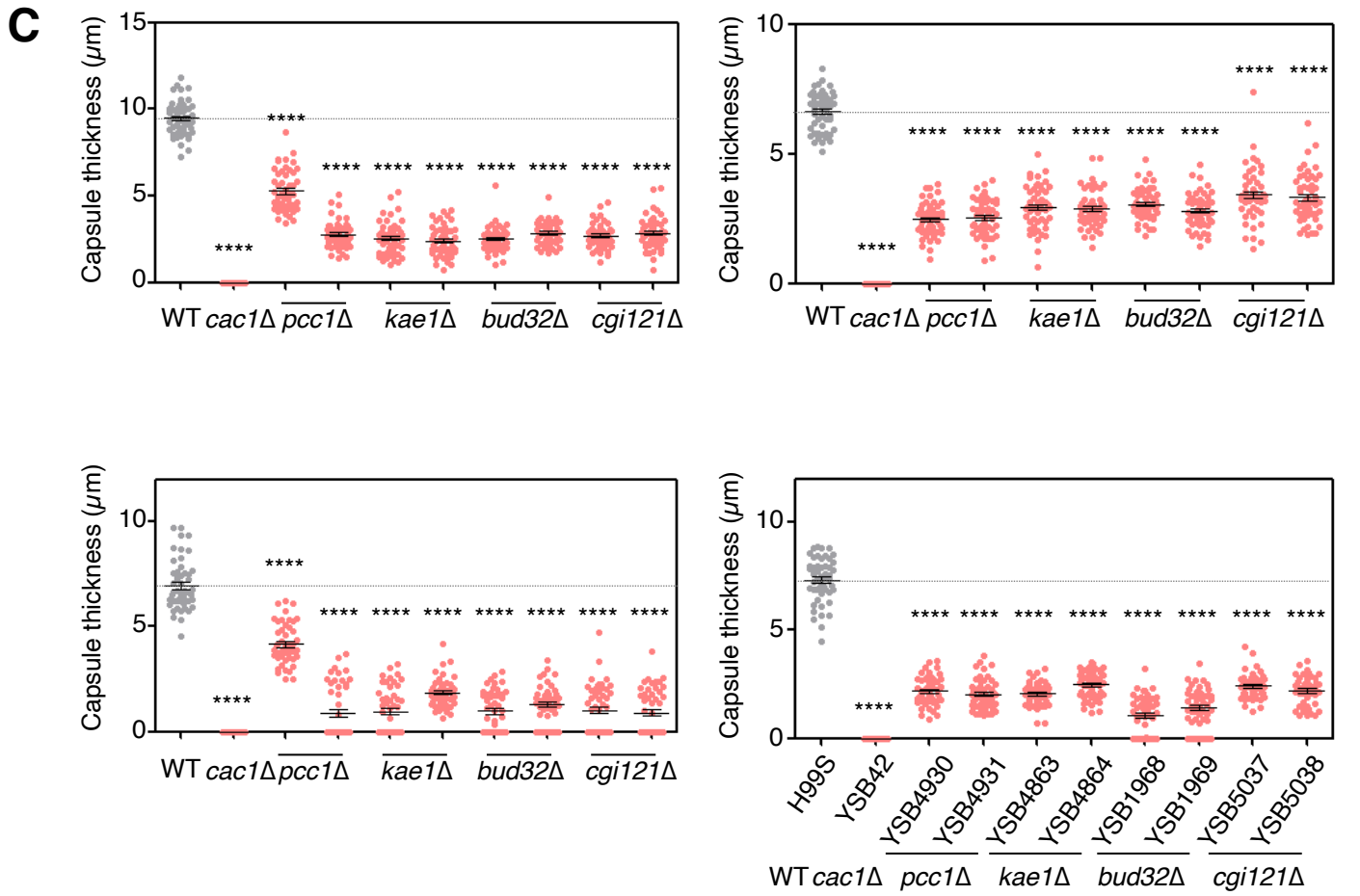

Supplement: FIG S4 [file mbio.02944-22-sf004.pdf]

Figure S6

A

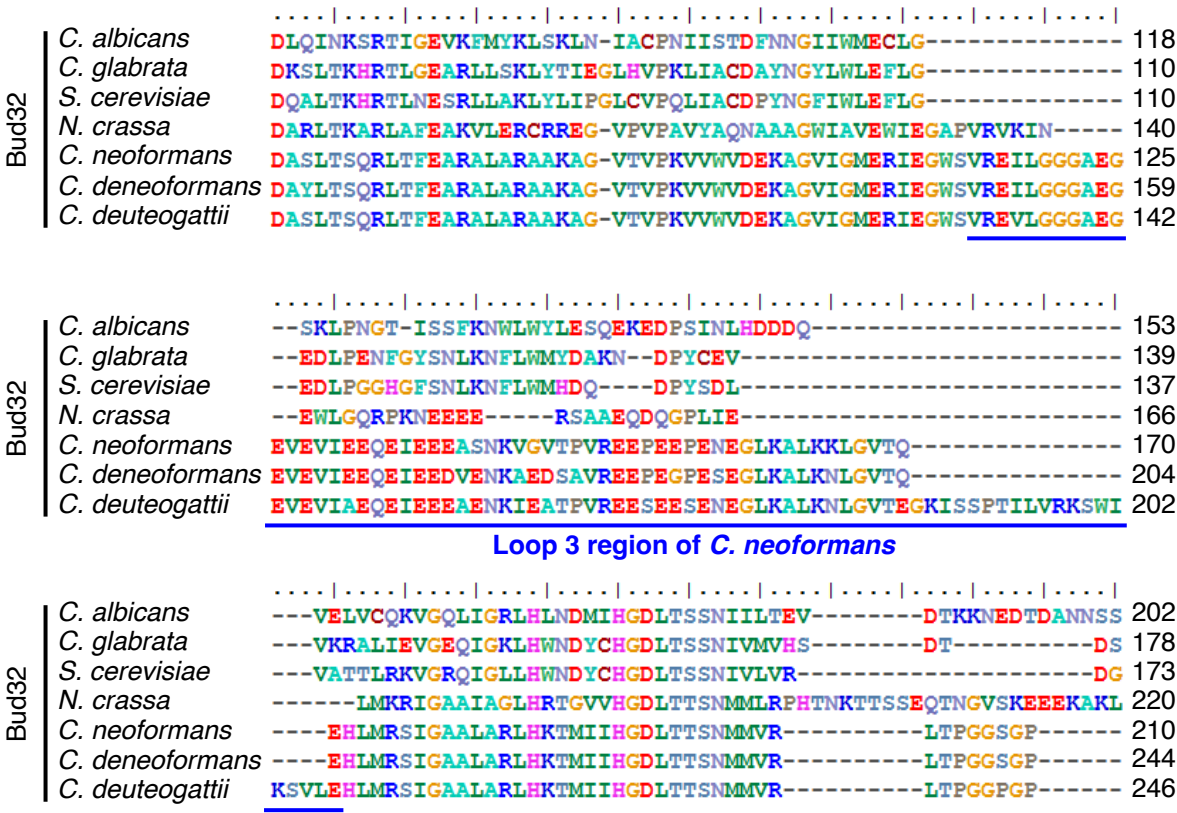

B

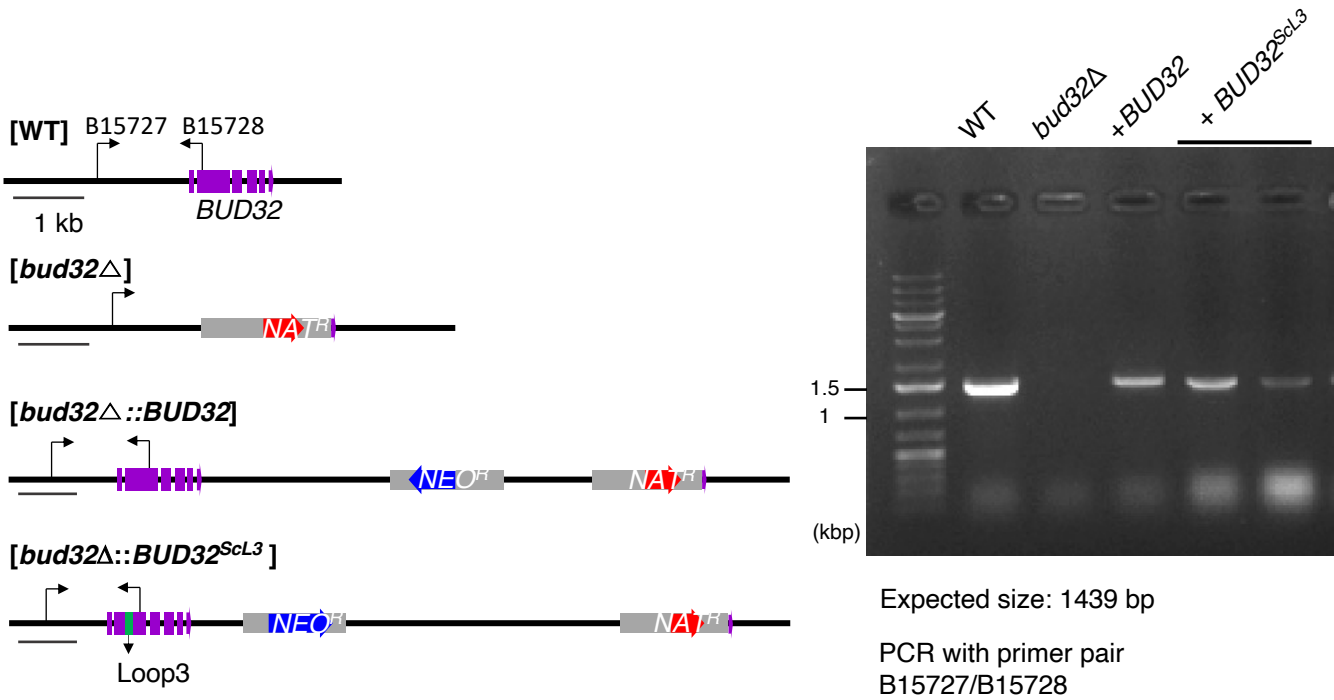

C

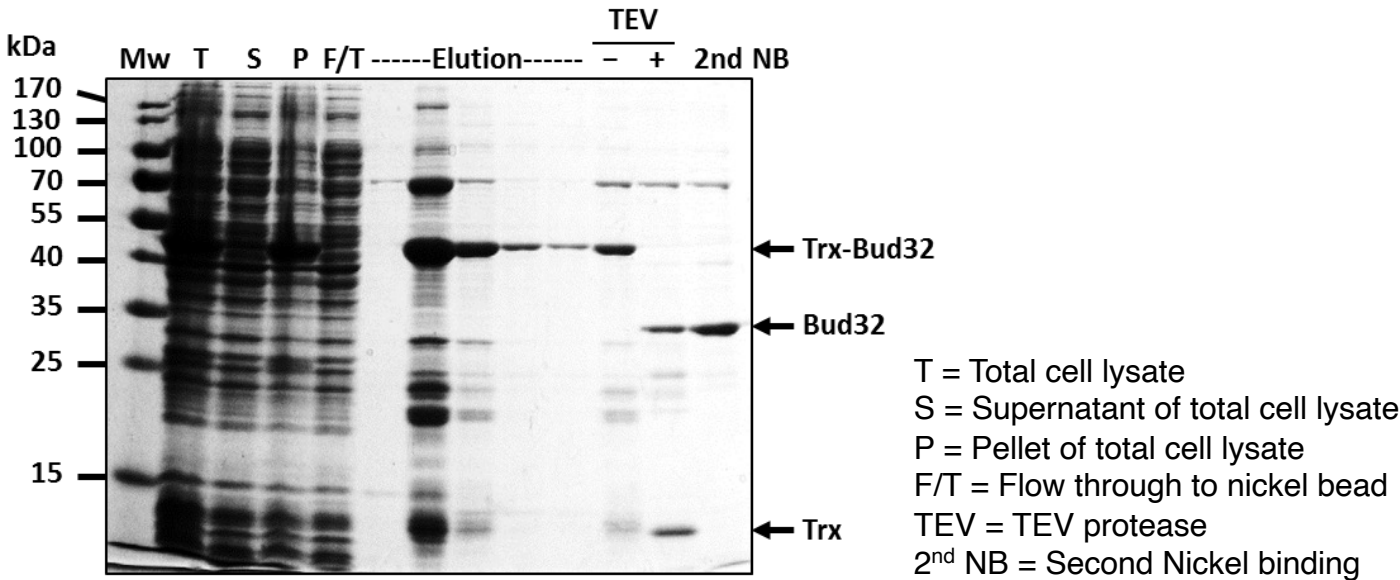

Figure S6

D

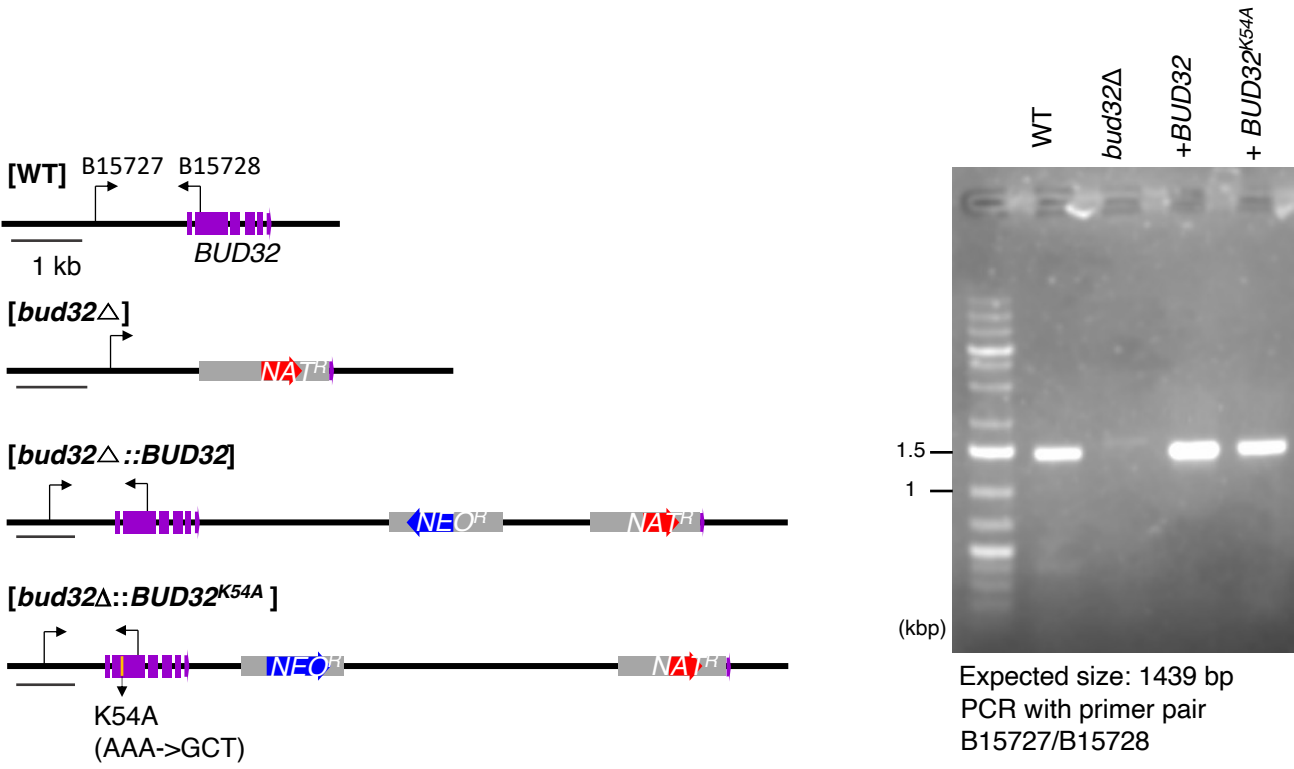

E

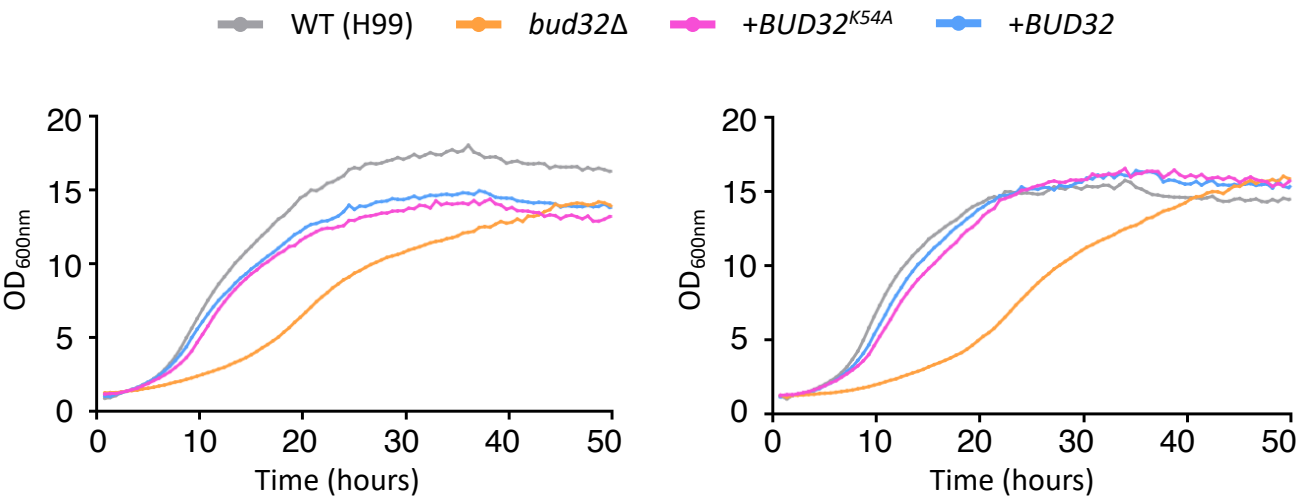

F

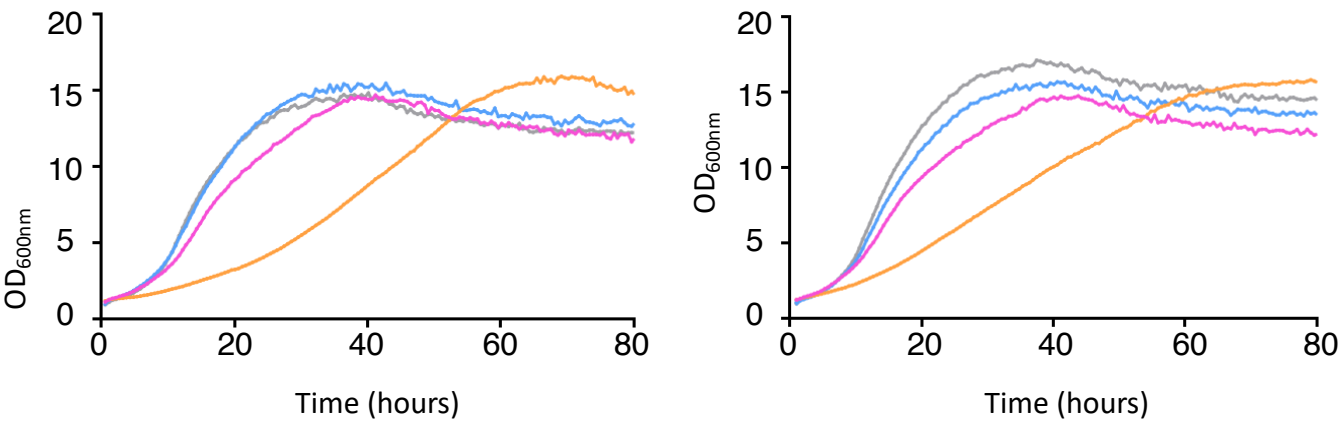

Figure S6

G

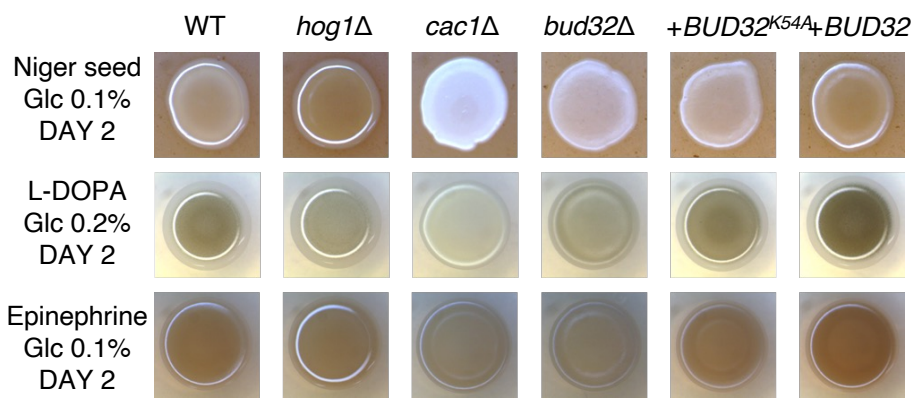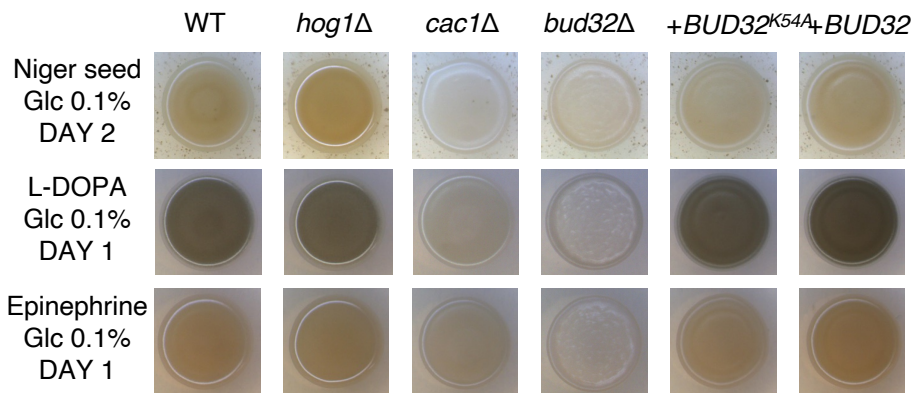

H

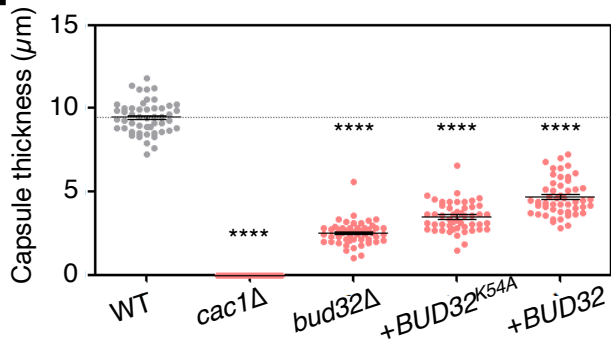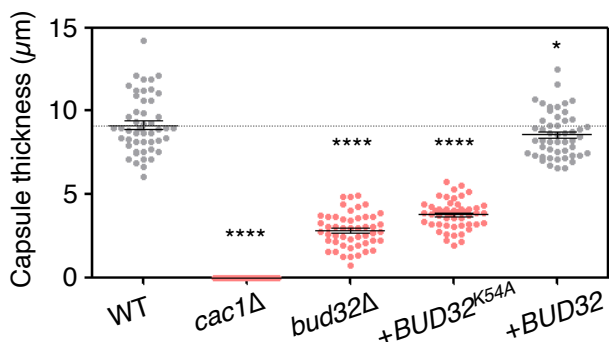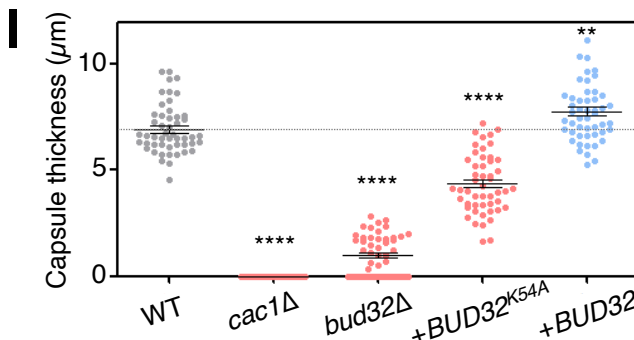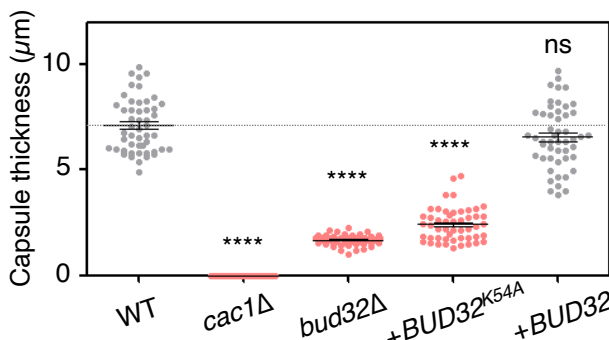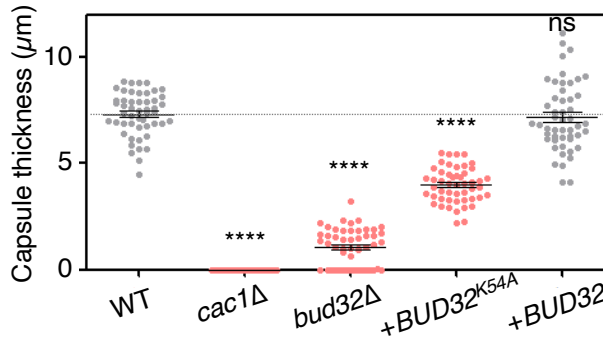

J

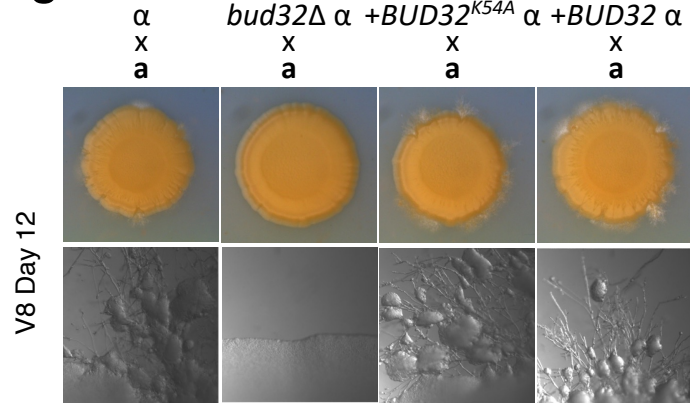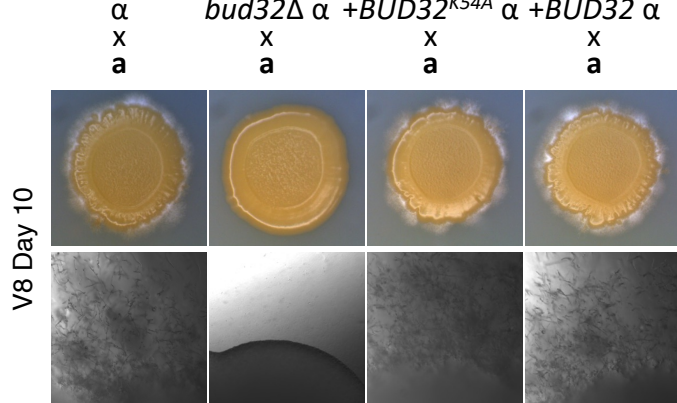

Supplement: FIG S6 [file mbio.02944-22-sf006.pdf]

Figure S7

A

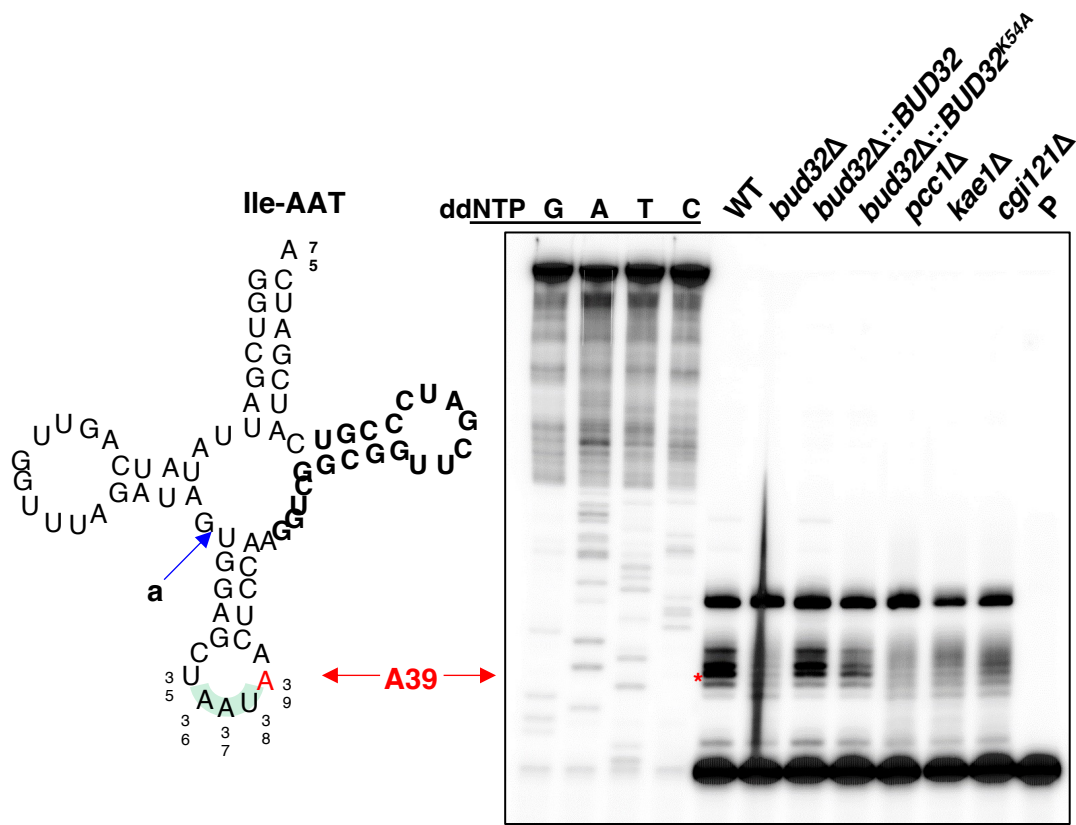

Supplement: FIG S7 [file mbio.02944-22-sf007.pdf]

Figure S8

A

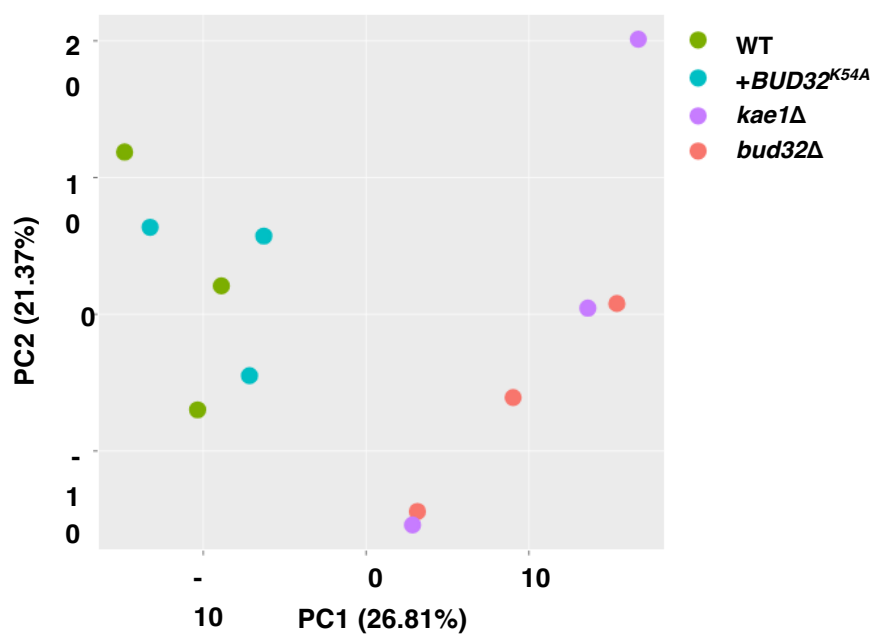

B

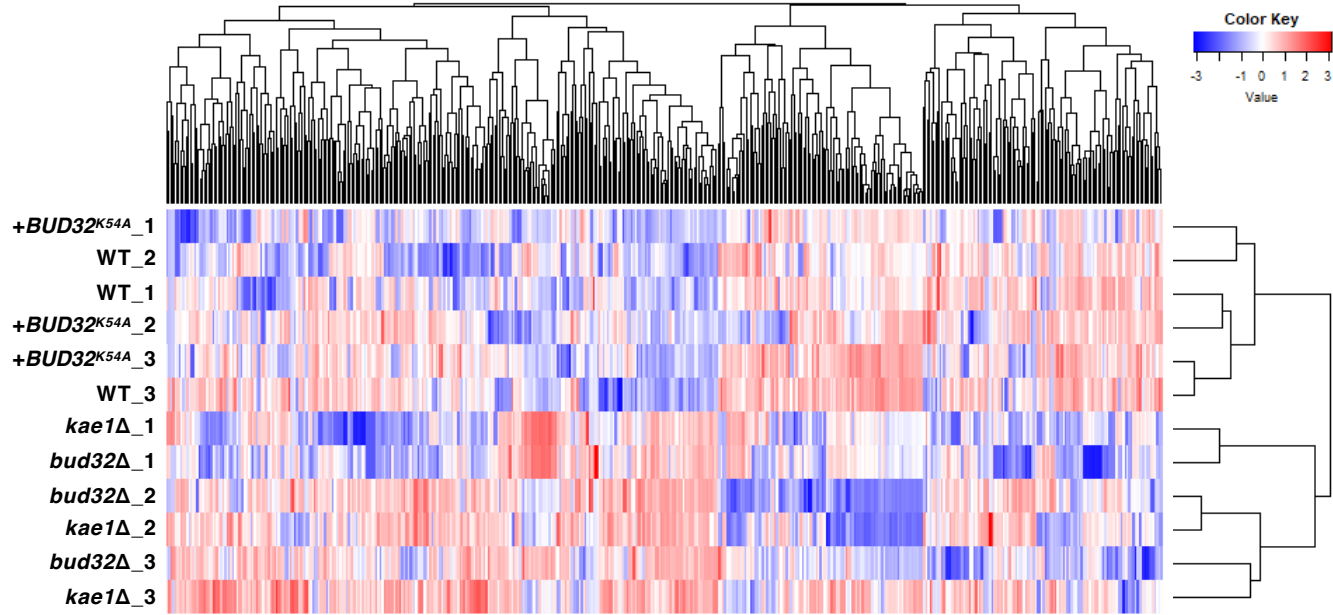

Supplement: FIG S8 [file mbio.02944-22-sf008.pdf]
