# Supplementary material for: Unraveling the Pathobiological Role of the Fungal KEOPS Complex in Cryptococcus neoformans
Source: mBio. 2022 Nov 15;13(6):e02944-22. doi: 10.1128/mbio.02944-22 (PMC9765431; doi:10.1128/mbio.02944-22)
Supplement: FIG S5 [file mbio.02944-22-sf005.pdf]

Figure S5

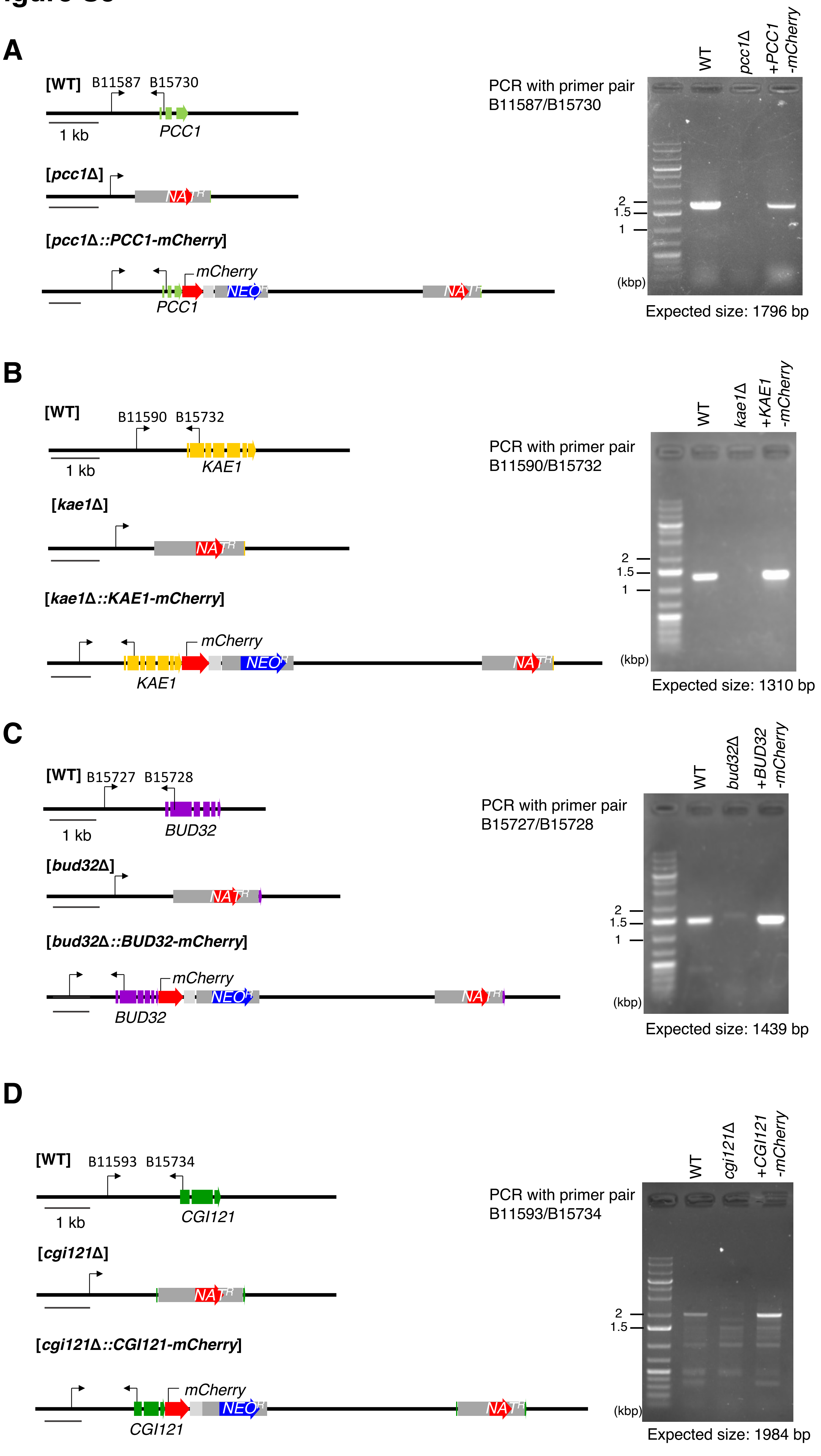

A
[WT]
B11587
B15730
1 kb
PCC1
[pcc1Δ]
NAT^R
[pcc1Δ::PCC1-mCherry]
mCherry
NEO^R
NAT^R
PCC1
PCR with primer pair
B11587/B15730
WT
pcc1Δ
+PCC1
-mCherry
2
1.5
1
(kbp)
Expected size: 1796 bp
B
[WT]
B11590
B15732
1 kb
KAE1
[kae1Δ]
NAT^R
[kae1Δ::KAE1-mCherry]
mCherry
NEO^R
NAT^R
KAE1
PCR with primer pair
B11590/B15732
WT
kae1Δ
+KAE1
-mCherry
2
1.5
1
(kbp)
Expected size: 1310 bp
C
[WT]
B15727
B15728
1 kb
BUD32
[bud32Δ]
NAT^R
[bud32Δ::BUD32-mCherry]
mCherry
NEO^R
NAT^R
BUD32
PCR with primer pair
B15727/B15728
WT
bud32Δ
+BUD32
-mCherry
2
1.5
1
(kbp)
Expected size: 1439 bp
D
[WT]
B11593
B15734
1 kb
CGI121
[cgi121Δ]
NAT^R
[cgi121Δ::CGI121-mCherry]
mCherry
NEO^R
NAT^R
CGI121
PCR with primer pair
B11593/B15734
WT
cgi121Δ
+CGI121
-mCherry
2
1.5
(kbp)
Expected size: 1984 bp

Figure S5

E

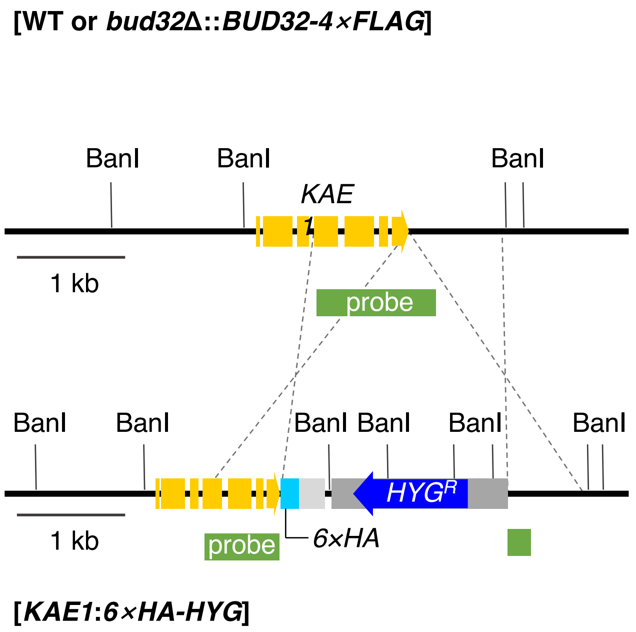

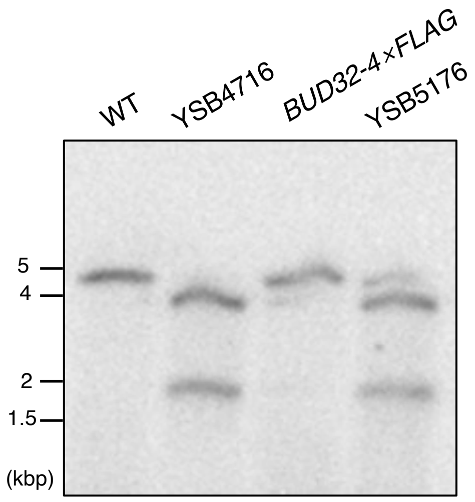

Expected size (AfeI digestion)
WT or *+BUD32-4×FLAG*: 4761 bp
Mutant: 1764 and 4553 bp

F

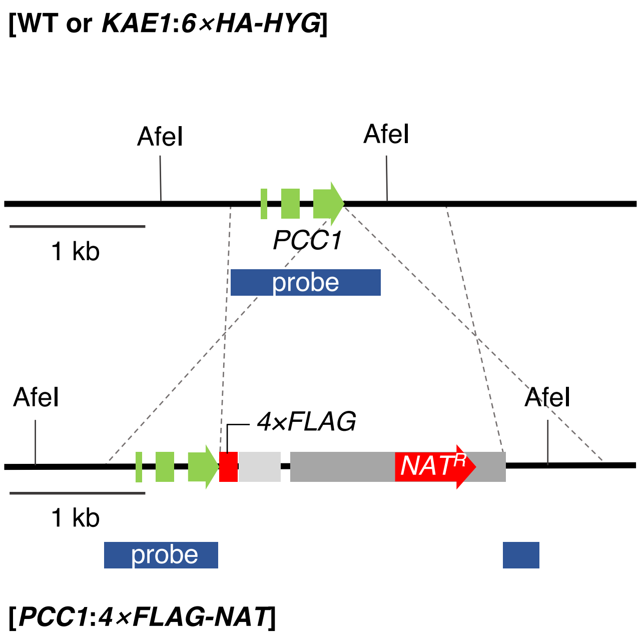

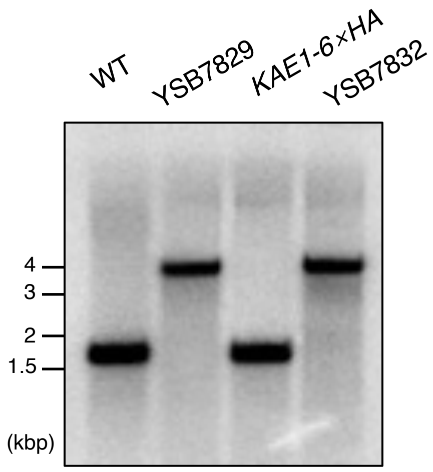

Expected size (AfeI digestion)
WT or *KAE1-6×HA*: 1678 bp
Mutant: 3754 bp

Figure S5

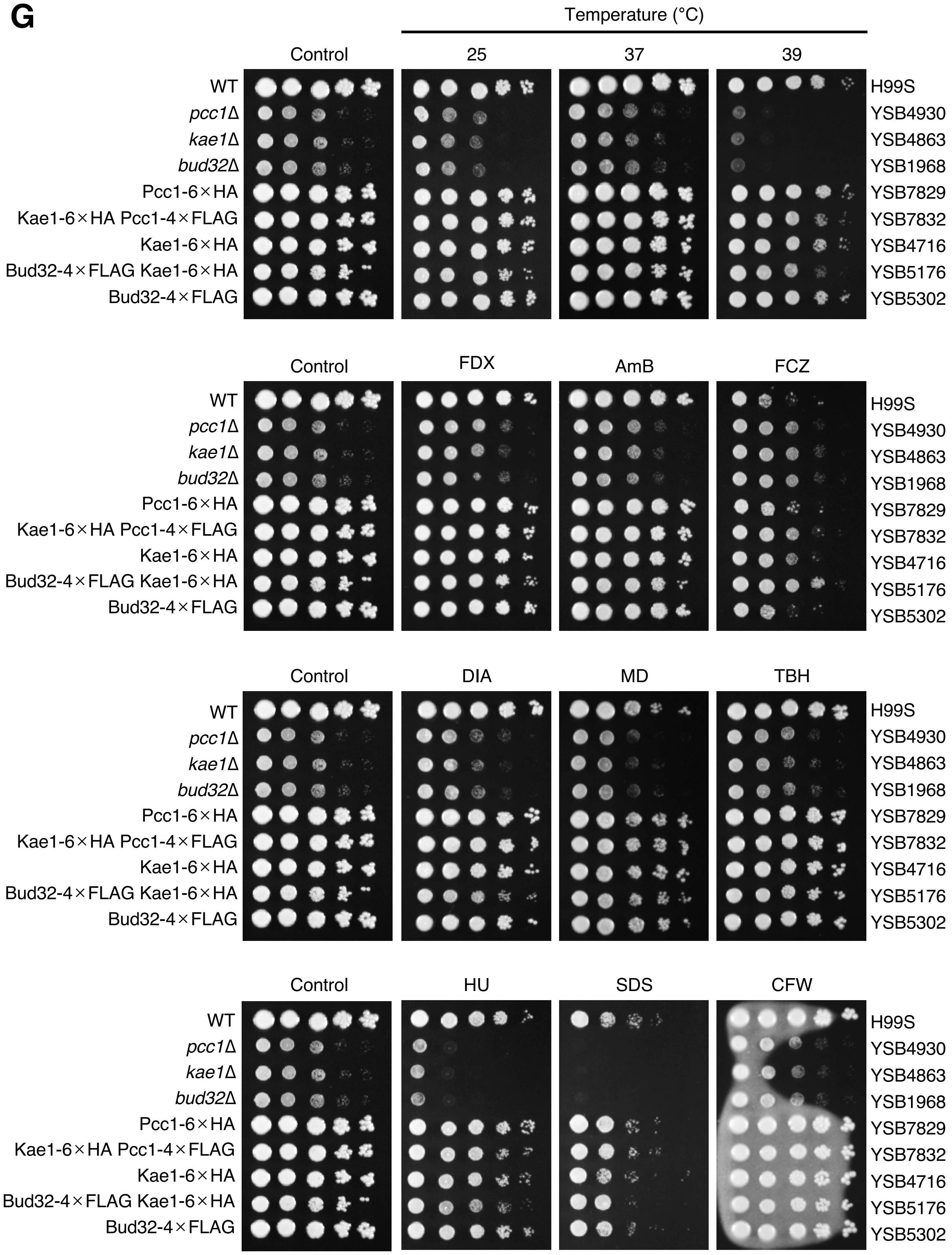
G
Temperature (°C)
Control
25
37
39
FDX
AmB
FCZ
DIA
MD
TBH
HU
SDS
CFW
WT
pcc1Δ
kae1Δ
bud32Δ
Pcc1-6×HA
Kae1-6×HA Pcc1-4×FLAG
Kae1-6×HA
Bud32-4×FLAG Kae1-6×HA
Bud32-4×FLAG
H99S
YSB4930
YSB4863
YSB1968
YSB7829
YSB7832
YSB4716
YSB5176
YSB5302

Figure S5

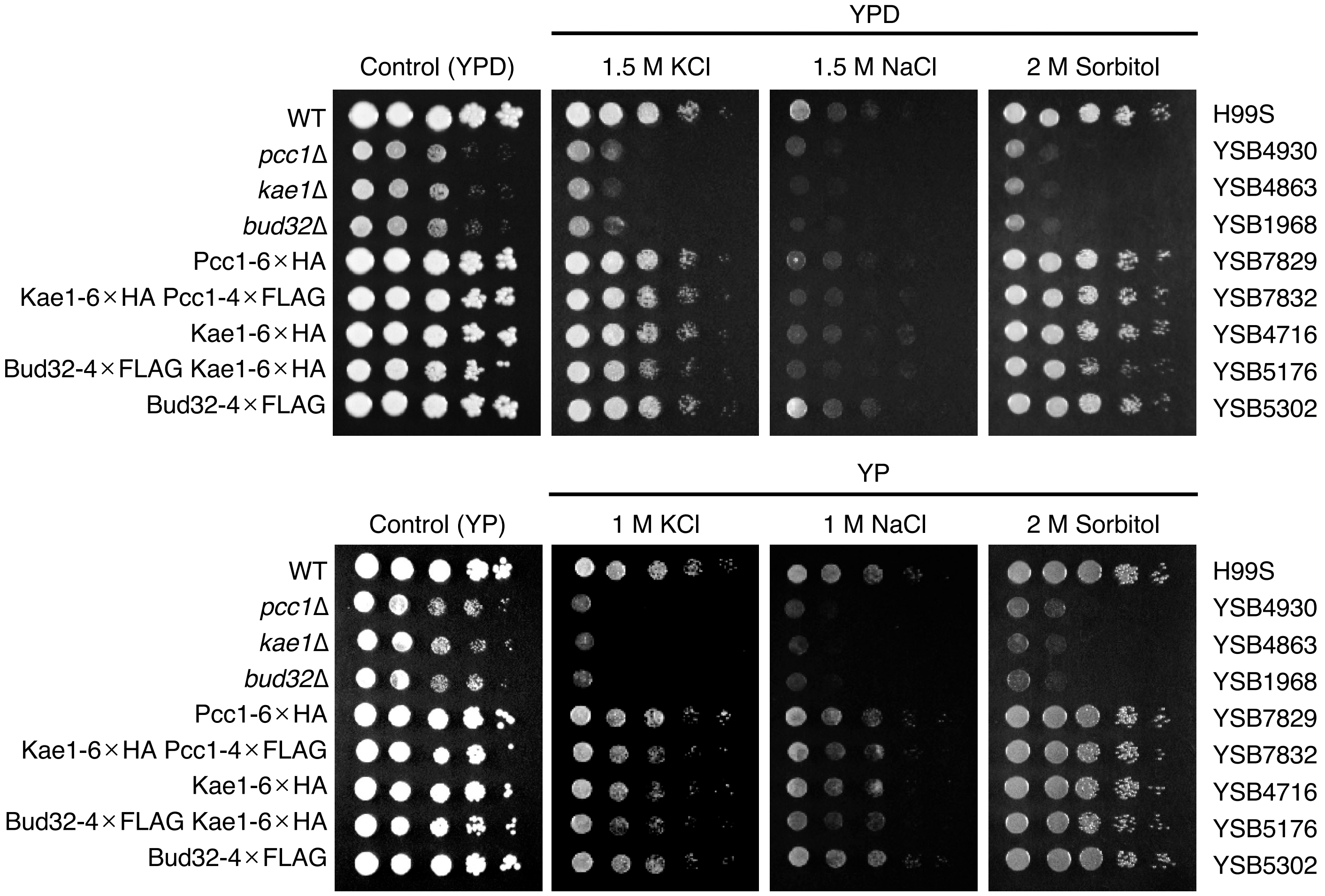

YPD
Control (YPD)
1.5 M KCl
1.5 M NaCl
2 M Sorbitol
WT
pcc1Δ
kae1Δ
bud32Δ
Pcc1-6×HA
Kae1-6×HA Pcc1-4×FLAG
Kae1-6×HA
Bud32-4×FLAG Kae1-6×HA
Bud32-4×FLAG
H99S
YSB4930
YSB4863
YSB1968
YSB7829
YSB7832
YSB4716
YSB5176
YSB5302
YP
Control (YP)
1 M KCl
1 M NaCl
2 M Sorbitol
WT
pcc1Δ
kae1Δ
bud32Δ
Pcc1-6×HA
Kae1-6×HA Pcc1-4×FLAG
Kae1-6×HA
Bud32-4×FLAG Kae1-6×HA
Bud32-4×FLAG
H99S
YSB4930
YSB4863
YSB1968
YSB7829
YSB7832
YSB4716
YSB5176
YSB5302

Figure S5

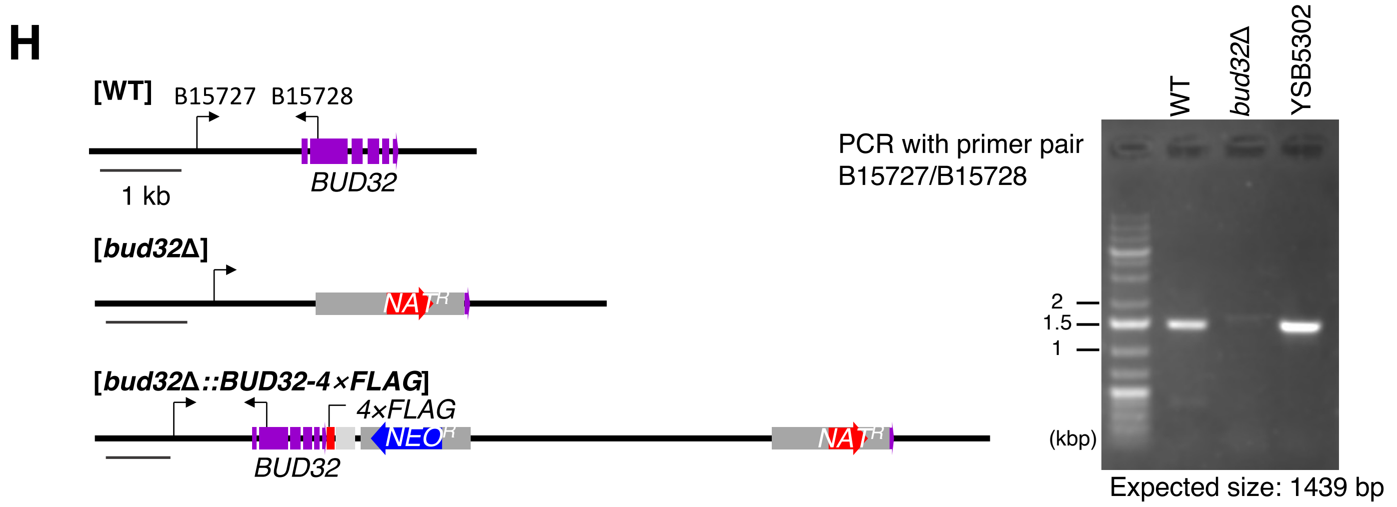
H
[WT]
B15727 B15728
1 kb
BUD32
[bud32Δ]
NAT^R
[bud32Δ::BUD32-4×FLAG]
4×FLAG
NEO^R
BUD32
NAT^R
PCR with primer pair B15727/B15728
WT
bud32Δ
YSB5302
2
1.5
1
(kbp)
Expected size: 1439 bp

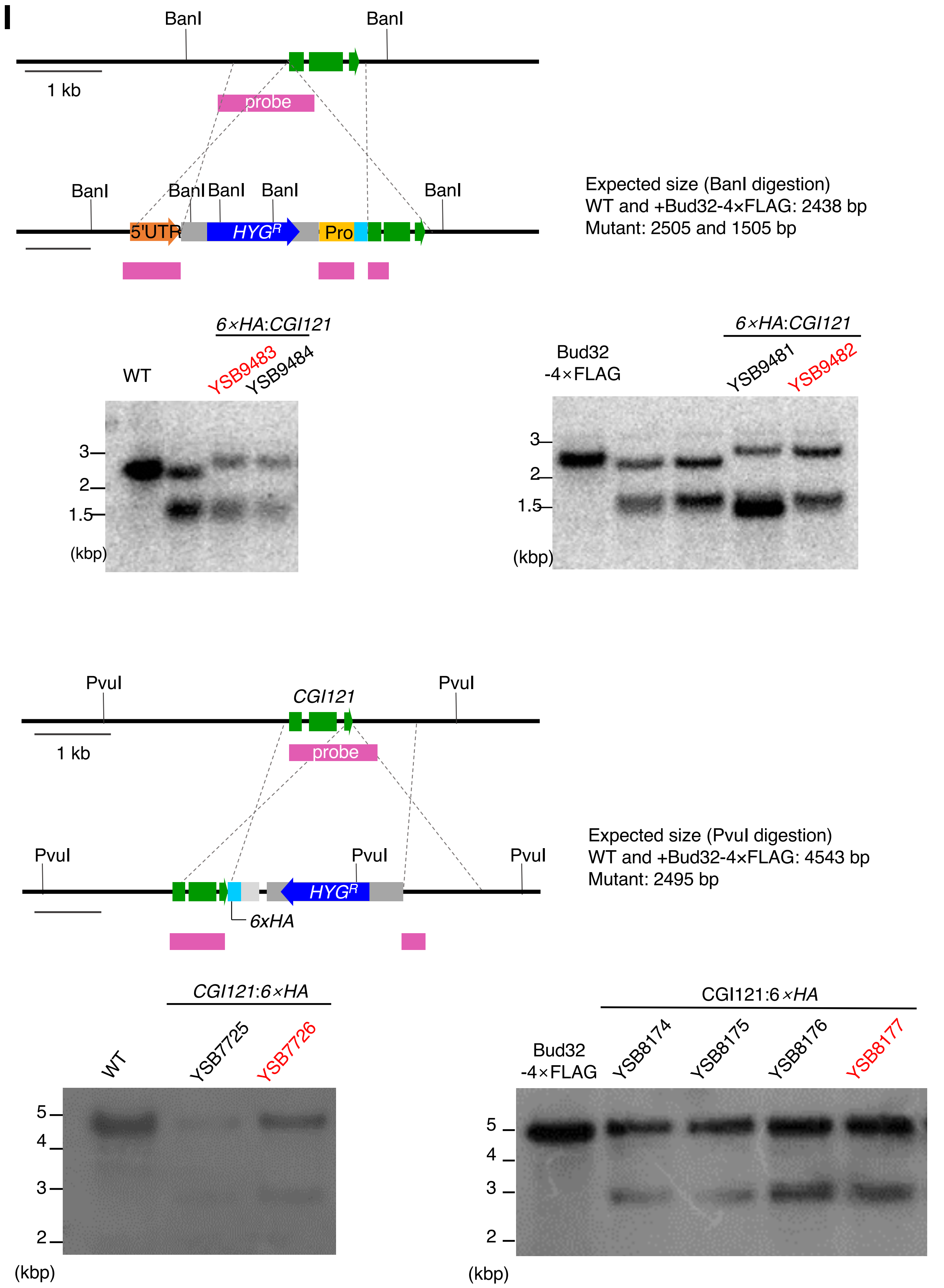
I
BanI
BanI
1 kb
probe
BanI
BanI BanI
BanI
BanI
5'UTR
HYG^R
Pro
Expected size (BanI digestion)
WT and +Bud32-4×FLAG: 2438 bp
Mutant: 2505 and 1505 bp
6×HA:CGI121
WT
YSB9483
YSB9484
3
2
1.5
(kbp)
Bud32 -4×FLAG
6×HA:CGI121
YSB9481
YSB9482
3
2
1.5
(kbp)
PvuI
CGI121
PvuI
1 kb
probe
PvuI
PvuI
PvuI
HYG^R
6xHA
Expected size (PvuI digestion)
WT and +Bud32-4×FLAG: 4543 bp
Mutant: 2495 bp
CGI121:6×HA
WT
YSB7725
YSB7726
5
4
3
2
(kbp)
CGI121:6×HA
Bud32 -4×FLAG
YSB8174
YSB8175
YSB8176
YSB8177
5
4
3
2
(kbp)

Figure S5

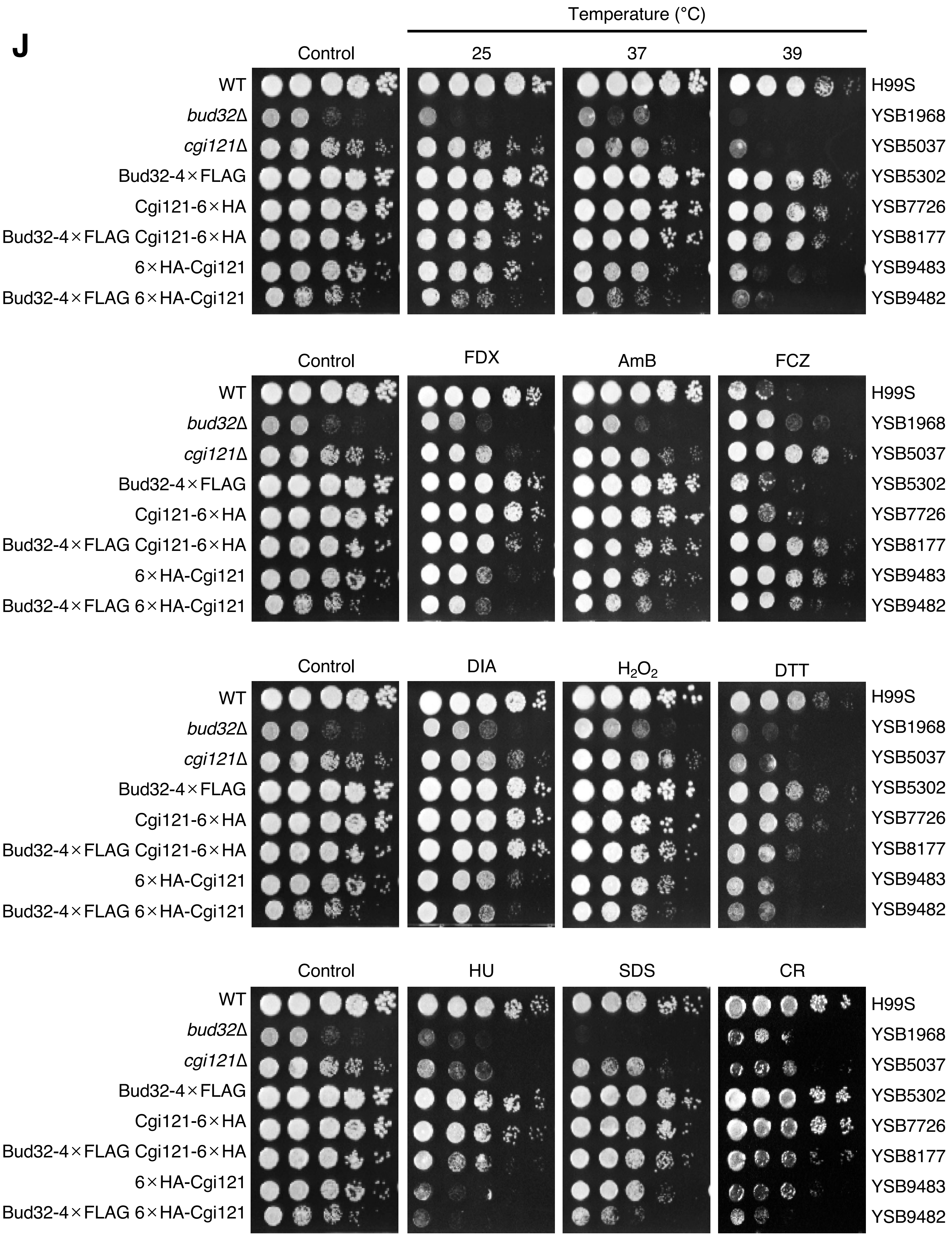
J
Temperature (°C)
Control
25
37
39
FDX
AmB
FCZ
DIA
H2O2
DTT
HU
SDS
CR
WT
bud32Δ
cgi121Δ
Bud32-4×FLAG
Cgi121-6×HA
Bud32-4×FLAG Cgi121-6×HA
6×HA-Cgi121
Bud32-4×FLAG 6×HA-Cgi121
H99S
YSB1968
YSB5037
YSB5302
YSB7726
YSB8177
YSB9483
YSB9482

Figure S5

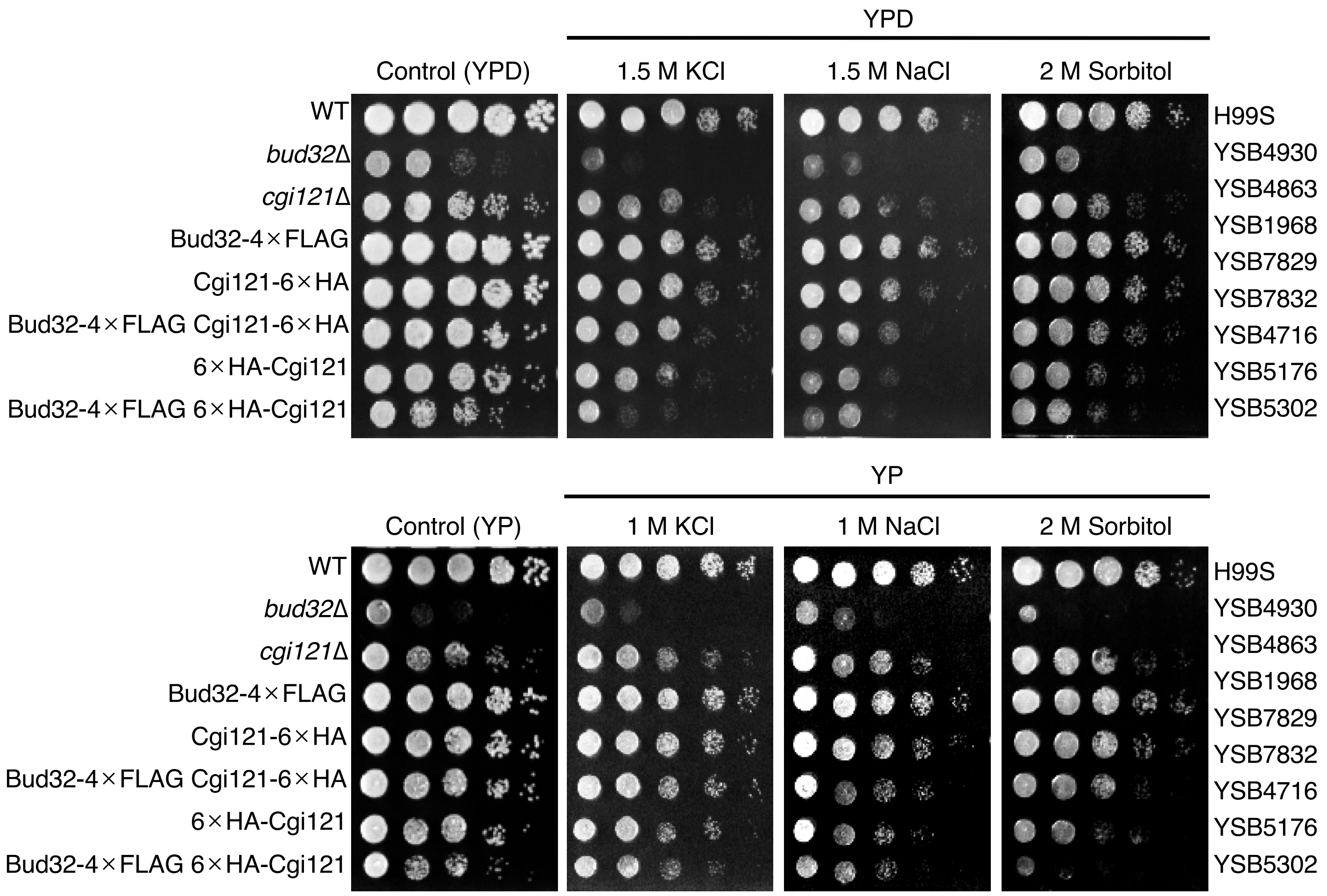

YPD
Control (YPD)
1.5 M KCl
1.5 M NaCl
2 M Sorbitol
WT
bud32Δ
cgi121Δ
Bud32-4×FLAG
Cgi121-6×HA
Bud32-4×FLAG Cgi121-6×HA
6×HA-Cgi121
Bud32-4×FLAG 6×HA-Cgi121
H99S
YSB4930
YSB4863
YSB1968
YSB7829
YSB7832
YSB4716
YSB5176
YSB5302
YP
Control (YP)
1 M KCl
1 M NaCl
2 M Sorbitol
WT
bud32Δ
cgi121Δ
Bud32-4×FLAG
Cgi121-6×HA
Bud32-4×FLAG Cgi121-6×HA
6×HA-Cgi121
Bud32-4×FLAG 6×HA-Cgi121
H99S
YSB4930
YSB4863
YSB1968
YSB7829
YSB7832
YSB4716
YSB5176
YSB5302

Figure S5

K

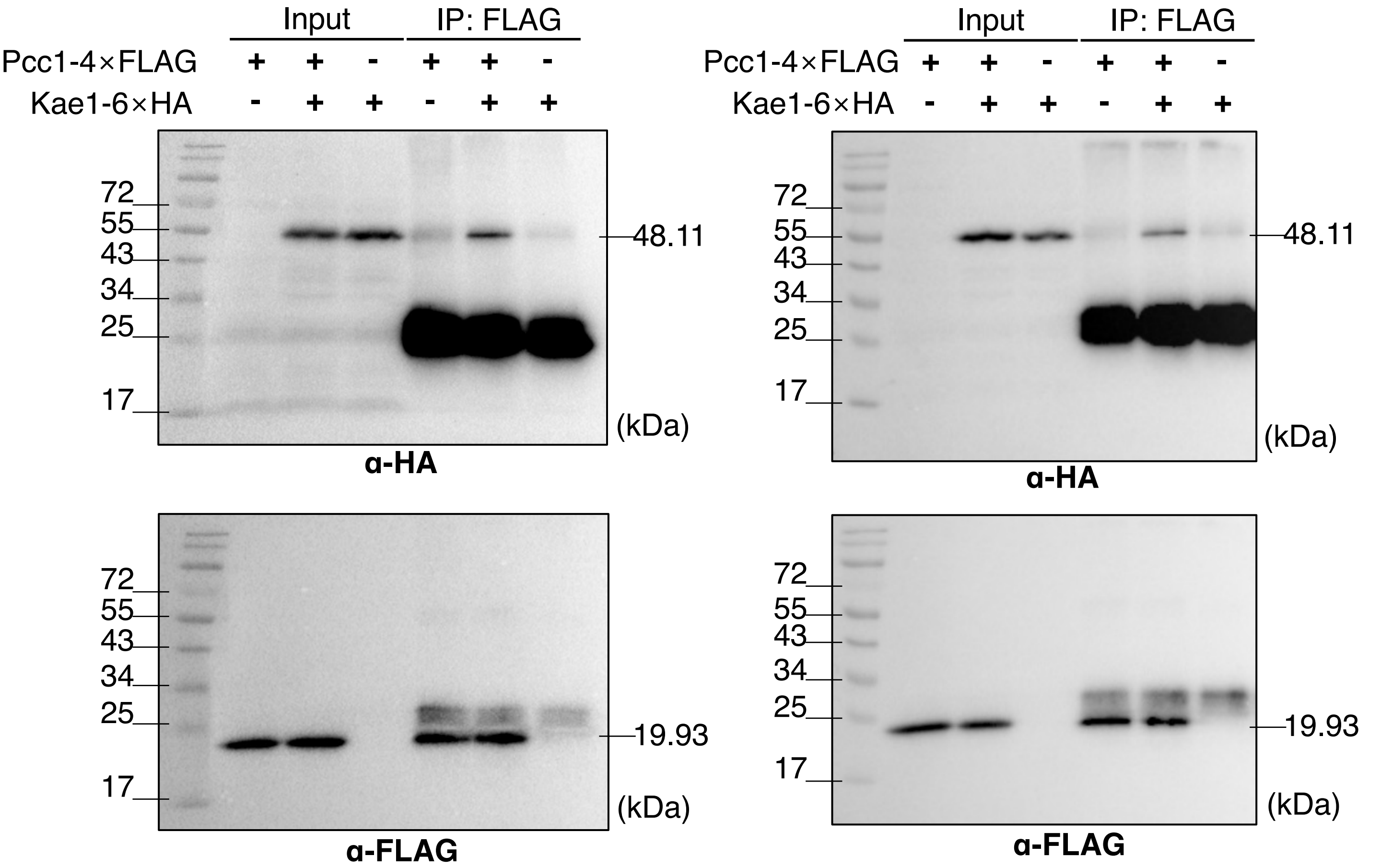
Input
IP: FLAG
Pcc1-4×FLAG + + - + + -
Kae1-6×HA - + + - + +
72
55
43
34
25
17
48.11
19.93
(kDa)
α-HA
α-FLAG

L

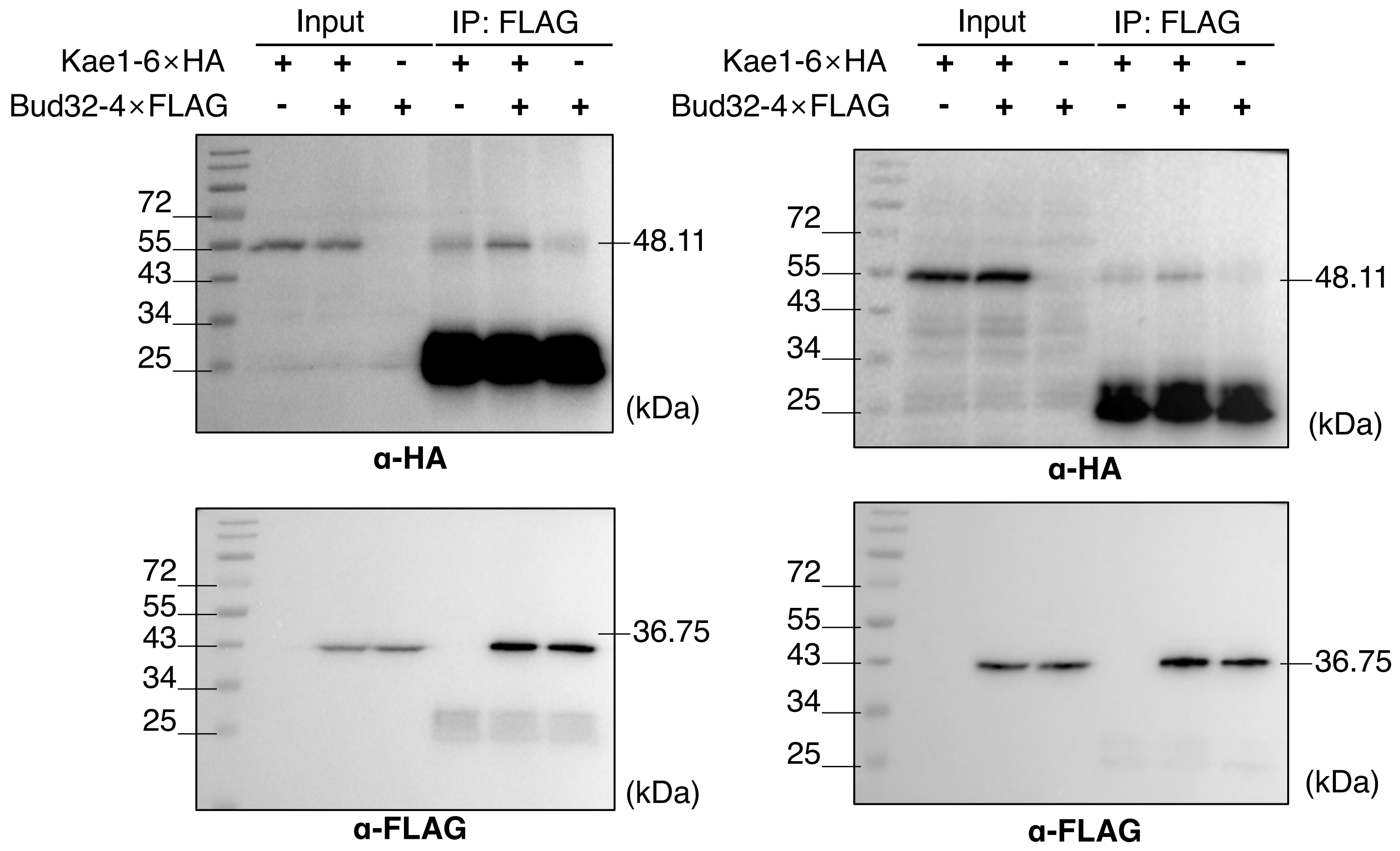
Input
IP: FLAG
Kae1-6×HA + + - + + -
Bud32-4×FLAG - + + - + +
72
55
43
34
25
48.11
36.75
(kDa)
α-HA
α-FLAG
